# Genomic Footprints of Multiple Host Lineages in the Mitochondrial and Nuclear Genomes of the Holoparasite *Prosopanche americana*

**DOI:** 10.3390/plants15071121

**Published:** 2026-04-07

**Authors:** Laura E. Garcia, Maria Emilia Roulet, Lucía A. Garay, M. Virginia Sanchez-Puerta

**Affiliations:** 1 Instituto de Biología Agrícola de Mendoza, Facultad de Ciencias Agrarias, CONICET, Universidad Nacional de Cuyo, Almirante Brown 500, Chacras de Coria, Mendoza M5528AHB, Argentina; 2 Facultad de Ciencias Exactas y Naturales, Universidad Nacional de Cuyo, Padre Jorge Contreras 1300, Mendoza M5502JMA, Argentina

**Keywords:** holoparasitic plants, mitochondrial DNA, nuclear DNA

## Abstract

Horizontal Gene Transfer (HGT) is a hallmark of the evolution of parasitic plants, facilitated by the haustorial connection. While mitochondrial HGT is widespread, the extent of nuclear HGT and the long-term retention of foreign genetic material in holoparasitic lineages remain poorly understood. This study explores the genomic architecture of *Prosopanche americana* (Hydnoraceae), a non-photosynthetic holoparasite currently specialized on Fabaceae. Through a comparative phylogenomic approach integrating draft mitochondrial genomes (mtDNA) and nuclear transcriptomes of *P. americana*, we identified a multi-layered landscape of foreign DNA. The mtDNA of *P. americana* contains 18 foreign regions (>500 bp) primarily derived from Solanales, Malvales, and Fabales. Notably, 13 of these regions are shared with *P. panguanensis*, indicating they were acquired in their common ancestor before speciation and ecological shift. In the nuclear genome, we identified 303 horizontally acquired transcripts (99 orthogroups) with high confidence. Functional analysis revealed an enrichment of foreign genes involved in metabolic pathways and plastid functions (e.g., photosystems and thylakoids) exclusively derived from the ancestral host order Solanales. Our results demonstrate that the genome of *P. americana* acts as a “molecular fossil,” preserving evidence of past ecological interactions with diverse host lineages. The disparity in HGT footprints between the current host (Fabaceae) and ancestral hosts suggests a period of high genomic plasticity followed by host specialization, providing new insights into the timing and dynamics of horizontal gene flow in holoparasitic Piperales.

## 1. Introduction

Parasitic plants represent one of the most intriguing ecological guilds, having evolved at least 12 times independently throughout the evolutionary history of flowering plants [1]. The transition to parasitism entails profound morphological and physiological modifications, the most critical being the development of the haustorium. This specialized organ not only functions as a physiological bridge for water and nutrient extraction but also establishes an intimate, cell-to-cell physical connection between the parasite and its host [2].

This physical intimacy has unexpected genomic consequences: the haustorium facilitates the exchange of macromolecules, including RNA and DNA, promoting Horizontal Gene Transfer (HGT) at rates significantly higher than in free-living plants [3,4,5,6,7]. Although HGT occurs across various eukaryotic lineages, this haustorial connection may drive a disproportionate accumulation of foreign genetic material in parasitic plants. This accumulation is particularly striking in non-photosynthetic holoparasites, which depend entirely on their host for survival [8,9]. HGT is especially prevalent in the mitochondrial genome (mtDNA) of parasitic plants. In certain lineages (e.g., Rafflesiaceae, Orobanchaceae, *Cynomorium*, *Lophophytum*), the mtDNA can incorporate genes from multiple historical and current hosts [4,5,10,11].

The dynamics of these horizontal acquisitions and the evolutionary fate vary considerably among holoparasitic plants. While mtDNAs in some lineages retain large, often functional foreign fragments [12,13,14], close relatives may exhibit virtually no mitochondrial HGT [15]. In addition, evidence from the holoparasitic *Cuscuta* and Orobanchaceae reveals that significant nuclear HGT can occur independently of mitochondrial dynamics. In these lineages, the nuclear genome has acquired multiple functional nuclear genes, despite the absence of major mitochondrial HGT [8,16]. A comprehensive understanding of the full extent and diversity of HGT in holoparasites requires the exploration of remaining key lineages, including the holoparasitic Hydnoraceae (Piperales), which comprise the genera *Hydnora* and *Prosopanche*. Despite the loss of photosynthetic capacity and the reduction in their plastome [15], little is known about the full extent of horizontal gene flow that impacts their mitochondrial and nuclear genomes, as well as the timing of these evolutionary events.

The genus *Prosopanche* (comprising seven species) exhibits a documented host range spanning 11 diverse angiosperm families, including Solanaceae, Malvaceae, Fabaceae, and Euphorbiaceae, among others [17]. However, the host range diversity is not uniformly distributed across species of *Prosopanche*. While *P. bonacinae* is a generalist associated with all 11 reported host families, the remaining species are specialists, each restricted to a single host family. This is the case for *P. americana* and *P. panguanensis*, which have been exclusively documented to parasitize Fabaceae [18,19,20,21,22,23]. The draft mtDNA of *P. americana* exhibits limited signals of HGT in the coding regions, namely short patches within the genes *cox1* and *atp8* [24]. While the specific donor for the *atp8* sequence remains unidentified, the foreign region in *cox1* has been traced to specific angiosperm lineages (including members of Solanales and Euphorbiaceae) that correspond to the known host families of *Prosopanche*. However, the intergenic regions, which comprise ~90% of the mtDNA, remain unexplored, and the nuclear genome has likewise not been examined.

In this study, we present a comprehensive analysis of nuclear and mitochondrial HGT in *Prosopanche americana*. Using a comparative phylogenomic approach that integrates mitochondrial genomes and nuclear transcriptomes, we investigate the origin and timing of foreign DNA acquisitions. We leverage available data from sister species, *P. panguanensis* and *P. bonacinae*, to distinguish between recent and ancestral events. Our results uncover a multi-layered landscape wherein *P. americana* possesses genetic material from donors belonging to at least five distinct orders (including Solanales, Malvales, and Fabales), providing new insights into the extensive history of host interactions that have shaped this genomic architecture over time.

## 2. Materials and Methods

### 2.1. Mitochondrial Genome Assemblies

To detect and characterize mitochondrial HGT in *Prosopanche americana*, we analyzed the published draft mitochondrial DNA (mtDNA) sequence (Table S1). This genome was assembled into 43 contigs totaling 426,953 bp and contains 39 native protein-coding genes, two of which show evidence of chimerism due to partial recombination with a foreign homolog [24].

Draft mitochondrial genomes from *P. panguanensis* and *P. bonacinae* were assembled de novo from Illumina paired-end reads downloaded in SRA, NCBI (accessions SRR31419645 and SRR31419646, respectively) using GetOrganelle v.1.7.5 [25] with the embryophyte mitochondrial database (-F embplant_mt). To optimize graph resolution, we utilized specific k-mer lengths of 21, 85, and 105, with a maximum of 2 extension rounds. Mitochondrial contigs were identified via BLASTn (v.2.16.0) searches in Bandage with default parameters [26] using the *P. americana* mtDNA as query. This process recovered 168 mitochondrial contigs for *P. bonacinae* (total length: 132 kb) and 357 contigs for *P. panguanensis* (total length: 444 kb). Using the same BLASTn-based approach, protein-coding regions were identified in the assembled contigs to include in the phylogenetic analyses.

### 2.2. Identification of High-Confidence Mitochondrial HGT Candidate Intergenic Regions

The phylogenetic origin of the intergenic regions in the draft mtDNAs of *P. americana* and *P. panguanensis* was initially assessed by BLASTn searches against a suite of custom databases designed around the host spectrum of *Prosopanche* [17]. To account for ancestral HGT events from donors other than the actual hosts, these databases were constructed at the order level, including (i) reported hosts, incorporating NCBI sequences from Solanales (2 families, 120 species), Fabales (3 families, 121 species), Rosales (7 families, 143 species), Aquifoliales (1 family, 6 species), Apiales (2 families, 57 species), Sapindales (7 families, 43 species), Malvales (3 families, 47 species), Caryophyllales (10 families, 78 species), Malpighiales (15 families, 96 species), and Poales (15 families, 125 species); (ii) the native order, Piperales (3 families, 106 species); and (iii) the rest of the angiosperms (37 orders, 123 families, 781 species, excluding parasitic species to avoid confounding HGT signals). The database sequence retrieval was automated using the rentrez R v.1.2.4 package [27]. All available records up to August 2025 were downloaded, filtering for mitochondrial sequences with a length greater than 4000 bp (Table S2). BLASThits were plotted using the SUSHI R package v.1.20.0 [28].

To further evaluate the phylogenetic origin of the intergenic mitochondrial regions in the draft mtDNAs of *P. americana* and *P. panguanensis*, we implemented a pipeline integrating BLASTn filtering, interval collapsing, and taxon-aware conflict resolution. Initial BLASTn alignments were filtered to retain only high-confidence HGT hits (hits from reported hosts with percent identity ≥ 90%, alignment length ≥ 200 bp), and overlapping matches derived from the same donor lineage were subsequently merged into contiguous blocks. To resolve phylogenetic assignment conflicts where blocks from different lineages overlapped, we prioritized the taxon exhibiting the highest percent identity, utilizing bit score and alignment length as secondary tie-breakers. Finally, a greedy interval-selection algorithm was applied to generate a non-overlapping genomic tiling (“no-gap” strategy), ensuring that each genomic position was assigned to the strongest available evidence of foreign origin. This was visualized as a distribution of best organellar BLASTn hits across the mitochondrial contigs (see Results), where colors other than grey correspond to foreign mitochondrial DNA tracts from different donors (e.g., Solanaceae, Fabaceae). To confirm their foreign origin, phylogenetic trees were reconstructed for regions with BLASTn hits in at least two different lineages, provided they met a >60% coverage threshold. Homologous DNA sequences were aligned using AliView v.1.30 [29]. Model selection was performed using ModelFinder [30] (-m MFP), which identified GTR + G as the best-fit nucleotide substitution model according to the Bayesian Information Criterion (BIC). Trees were reconstructed using RAxML v.8 with 1000 bootstrap replicates under the GTRGAMMA model. The resulting topologies were visualized in iTOL [31].

To assess the timing of the HGT events, the foreign regions identified in *P. americana* were BLASTnearched against the mitochondrial contigs of *P. bonacinae* and *P. panguanensis*. This allowed us to evaluate the conservation of HGT events across the genus.

### 2.3. Sequencing and Assembly of the Prosopanche americana Transcriptome

A flower from an individual of *Prosopanche americana* (Hydnoraceae) parasitizing *Neltuma flexuosa* (Fabaceae) was collected in January 2023 in Phillips, Junín Department, Mendoza Province, Argentina (33°12′28.008′′ S 68°21′47.916′′ W). Total RNA was extracted using a protocol suitable for highly viscous samples rich in polysaccharides [32] and purified with the RNAqueous kit (Invitrogen, Waltham, MA, USA), which employs columns with glass fiber filters. Library construction and rRNA depletion were performed by the sequencing provider and sequenced on the Illumina Novaseq S6000 platform at Shanghai Neo-Biotechnology Co., Ltd., Shanghai, China. The obtained RNA-seq data were analyzed with FastQC v.0.12.1 [33]. The FastQC report showed that all parameters assessed were acceptable except for the ‘per base sequence content’ parameter, which displayed a deviation in the first 12 nucleotides of the reads. These nucleotides were trimmed using Trimmomatic v.0.40 [34] (parameter HEADCROP:12), resulting in a final read length of 138 bp for downstream analyses. The RNA was extracted from flowers of the holoparasitic plants for several reasons: (i) no leaves are present in these plants, (ii) flower tissues are not contaminated with host tissue, and (iii) success with inflorescences has been reported previously [5,14,35].

Reads quality-filtered with Trimmomatic were assembled de novo using Trinity v.2.8.4 [36], with the following parameters: SS_lib_type RF–min_contig_length 100 on the SARTOI computational cluster at the IBAM UNCuyo-CONICET. Open Reading Frames (ORFs) were extracted from the transcriptome assembly using TransDecoder v.5.7.0 (https://github.com/TransDecoder/TransDecoder accessed on 1 November 2025), which yielded an initial set of 120,164 ORFs exceeding 100 bp in length. The transcriptome integrity was assessed using BUSCO [37], which revealed complete gene recovery for approximately 91%, 52%, and 47% of single-copy and duplicated orthologs representing conserved gene families within Eukaryota (*n* = 303), Embryophyta (n = 1440), and Eudicots (n = 2121), respectively (Table S3). These recovery rates are consistent with expectations for the relatively complete transcriptome of a holoparasitic plant, given the likely widespread evolutionary loss of genes associated with photosynthetic functions and vegetative structures [13,38,39,40].

### 2.4. Orthogroups Inference and Identification of Foreign Nuclear Genes

A custom multi-stage bioinformatics pipeline was designed and implemented to identify genes acquired from its potential hosts into the *P. americana* nuclear genome. The workflow (Figure S1) encompassed (1) data preparation and filtering, (2) orthogroup (OG) identification, (3) candidate selection and phylogenetic inference, and (4) validation and curation of HGT events.

The initial set of ORFs underwent a rigorous sequential filtering process. First, 8893 sequences corresponding to transposable elements (TEs) were identified and removed using HMMER v.3.3.2 [41] with profiles from the Dfam database (e-value < 0.05). Subsequently, to eliminate biological contaminants (e.g., fungi, metazoans), the remaining 111,271 sequences were taxonomically annotated with eggNOG-mapper v.5.0 [42]. All sequences without an assignment within Viridiplantae (n = 15,812) were discarded, resulting in a high-confidence final transcriptome for *Prosopanche* composed of 95,459 sequences. Gene family identification was performed using OrthoFinder v.2.5.4 [43]. The analysis included the filtered *Prosopanche* translated ORFs along with the proteomes from 45 reference species, encompassing host lineages from the orders Solanales, Fabales, and Malvales, which were identified as donors in the *Prosopanche* mitochondrial genome, as well as pertinent outgroups (see Table S4 for the complete species list). This analysis generated 101,329 OGs. To restrict the phylogenetic analysis to high-probability HGT candidates, a two-step filtering strategy was applied:

- Similarity Filter (BLASTp)*:* We selected OGs where at least one *Prosopanche* sequence had its best hit (BLASTp, e-value < 1 × 10^−5^) against a species from the host families.
- Phylogenetic Informativeness: OGs with a minimum of four taxa were retained to ensure robust topological inference.

### 2.5. Phylogenetic Reconstruction of Nuclear Foreign Candidate Transcripts

The amino acid sequences for each candidate OG were aligned using MAFFT v.7 [44] (L-INS-i algorithm). To optimize the signal-to-noise ratio, the alignments were processed with ClipKIT v.2 [45] in gappy mode (-m gappy). The Q.plant substitution model was selected to account for plant-specific amino acid substitution biases. Model evaluation and parameter estimation were conducted using ModelFinder [30] implemented in IQ-TREE v.2.2 [46]. Phylogenetic reconstruction was conducted using maximum likelihood (ML) in IQ-TREE v.2.2, employing the Q.plant substitution model and 1000 replicates of Ultrafast Bootstrap (UFBoot).

### 2.6. Nuclear HGT Event Detection and Curation

HGT identification in phylogenetic analyses was automated via a custom R script using the ape package [47]. HGT events were considered when (i) the *Prosopanche* sequence was nested within a host family clade or positioned as a sister to the host family, and (ii) the basal node of the clade presented robust support (UFBoot > 95%).

The resulting OG containing foreign transcripts (n = 142) underwent exhaustive cleaning. To eliminate redundancy due to isoforms, CD-HIT (-c 0.95) was applied, followed by a manual selection of the longest isoform. Functional annotation (Mercator [48], InterProScan [49], eggNOG [42]) enabled us to rule out cryptic contaminants that were undetected in previous steps. Finally, to distinguish functional genes from potential transposons, sequences with domains or descriptions associated with TE machinery (e.g., reverse transcriptase, transposase) were excluded. The final high-confidence dataset consisted of 303 horizontally transferred transcripts.

### 2.7. Functional Annotation of Foreign Nuclear Genes

Gene Ontology (GO) enrichment analysis was performed using the PANTHER Classification System (v.19.0, release 7 August 2024; http://www.pantherdb.org accessed on 31 December 2025). We utilized the Statistical Overrepresentation Test to compare the input gene list against the *Arabidopsis thaliana* reference genome. Significance was determined using Fisher’s exact test with a False Discovery Rate (FDR) correction for multiple testing. Terms with an adjusted *p*-value (FDR) < 0.05 were considered significantly enriched.

## 3. Results

### 3.1. Characterization and Origin of Mitochondrial HGTs in Prosopanche americana

BLASTn analysis against custom databases, including the host orders of the genus *Prosopanche* [17], the order Piperales (to which *P. americana* belongs), and all other angiosperms (updated as of August 2025), revealed that 55% of the *P. americana* mtDNA is covered by hits from all angiosperm mtDNAs combined (Figure S2). This coverage includes 92,812 bp from Piperales, 40,384 bp from host orders, and 105,338 bp from other angiosperms (Figure S2).

**Figure 1:**
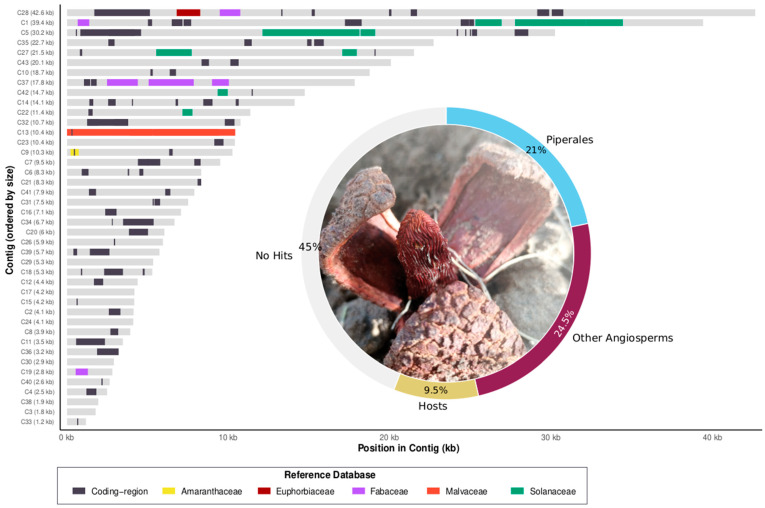
Horizontal Gene Transfer (HGT) landscape in *Prosopanche americana.*

A comprehensive inspection of the 43 contigs in the draft mtDNA of *P. americana* identified 18 distinct foreign regions exceeding 500 bp in length. The origin of these foreign regions was confirmed by phylogenetic analyses whenever possible; in other cases, donor assignment relied on the exclusive presence of the fragment in specific host lineages (Figure S3). The most heavily represented host orders as potential donors were Solanales (20.2 kb), Malvales (10.4 kb), and Fabales (8.5 kb) (Figure 1). Additionally, smaller fragments attributed to Euphorbiaceae (1460 bp) and Amaranthaceae (500 bp) were detected (Figure 1 and Figure S2; Table S5).

The graph displays the mitochondrial contigs, ordered by length (*Y*-axis). Each horizontal bar represents a single contig, with the *X*-axis showing the position in kilobases (kb). Colored segments represent regions identified as high-confidence HGT fragments, where the *P. americana* sequence showed its best BLAST hit against a specific host family. These hits met the filtering criteria: p-identity > 90 and length > 200 bp. The color key below the graph indicates the specific host family that provided the best hit for the HGT region (e.g., Fabaceae, Malvaceae, Solanaceae). Dark gray segments indicate the mitochondrial coding regions (genes) of *P. americana*. Light gray segments represent regions with no significant BLAST hits against any of the databases used. The donut chart (surrounding the photograph) illustrates the breakdown by origin of the total 426,953 bp assembly. The genomic origin is categorized into vertical inheritance (Piperales; 92,812 bp), HGT from host orders (40,384 bp), and conserved sequences from other angiosperms (105,338 bp). The remaining fraction (no hits) represents divergent sequences or those with no significant BLAST hits. The photograph shows a mature flower of *P. americana*.

To assess the timing of these acquisitions, we screened for these foreign regions in the draft mtDNAs of *P. panguanensis* and *P. bonacinae*. Notably, 13 of the 18 identified regions were present in *P. panguanensis*, while none were detected in *P. bonacinae*. It should be noted that draft assemblies are incomplete relative to the gene content of *P. americana* (*P. panguanensis* exhibits approximately 75% completeness, and *P. bonacinae* 65%, based on the protein gene content relative to *P. americana*). Consequently, the apparent absence of foreign sequences in the *P. bonacinae*—and potentially the missing regions in *P. panguanensis*—may be an artifact due to missing data. In all phylogenetic trees where *P. panguanensis* sequences were available, *P. americana* and *P. panguanensis* formed a monophyletic group, suggesting that these HGT events occurred in their common ancestor (Figure S3). These ancestral HGT events were identified as foreign DNA donated by all five host orders.

To further explore the evolutionary dynamics of HGT in the genus, we inspected the *P. panguanensis* draft mtDNA (198 contigs) and identified 58 distinct foreign regions exceeding 500 bp, distributed across 44 contigs (Figure S4). Interestingly, while *P. americana* retains Solanales as the predominant donor, the HGT landscape in *P. panguanensis* is dominated by Fabales (44 kb), followed by Solanales (13 kb), Malpighiales (6 kb), Malvales (4.5 kb), and Caryophyllales (4 kb). Additionally, smaller fragments attributed to Poales (800 bp) and Sapindales (700 bp) were detected (Figure S4), revealing a distinct HGT profile characterized by a marked enrichment of sequences from the current host lineage compared to *P. americana***.**

### 3.2. HGT Impact on Mitochondrial Coding Regions

Phylogenetic analyses of protein-coding genes in the three species of *Prosopanche* revealed a predominant native origin of all genes, in agreement with Yu et al. [24] (Figure S5). The exceptions were *cox1* and *atp8*, where the chimerism previously reported for *P. americana* [24] was also identified in *P. panguanensis* and *P. bonacinae* upon alignment inspection, in which short foreign regions were detected.

**Figure 2:**
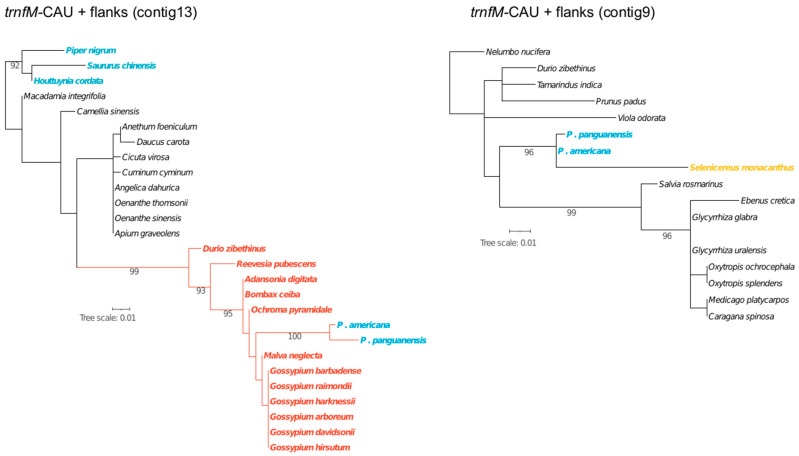
Evolutionary origin of foreign trnfM-CAU copies in the *Prosopanche americana* draft mitochondrial genome. Maximum likelihood phylogenetic analysis was based on alignments including the complete tRNA sequence and 100 bp of flanking regions of the gene copies located on contigs 13 and 9. Trees were reconstructed using RAxML under the GTRGAMMA substitution model with 1000 bootstrap replicates; nodal support values are indicated. Piperales are highlighted in blue, Malvales are highlighted in orange and Caryophyllales in yellow, demonstrating the independent horizontal acquisition of these loci in the common ancestor of *P. americana* and *P. panguanensis*.

Gene mapping within the HGT blocks revealed the presence of two foreign *trnfM*. One copy is located on contig 9 embedded within a genomic block derived from Caryophyllales, while the other resides on contig 13 within a Malvaceae-derived region. Phylogenetic analyses of the *trnfM* and 100 bp flanking regions confirmed these affinities (Figure 2).

### 3.3. HGT from Different Hosts in the Nuclear Genome of Prosopanche americana

**Figure 3:**
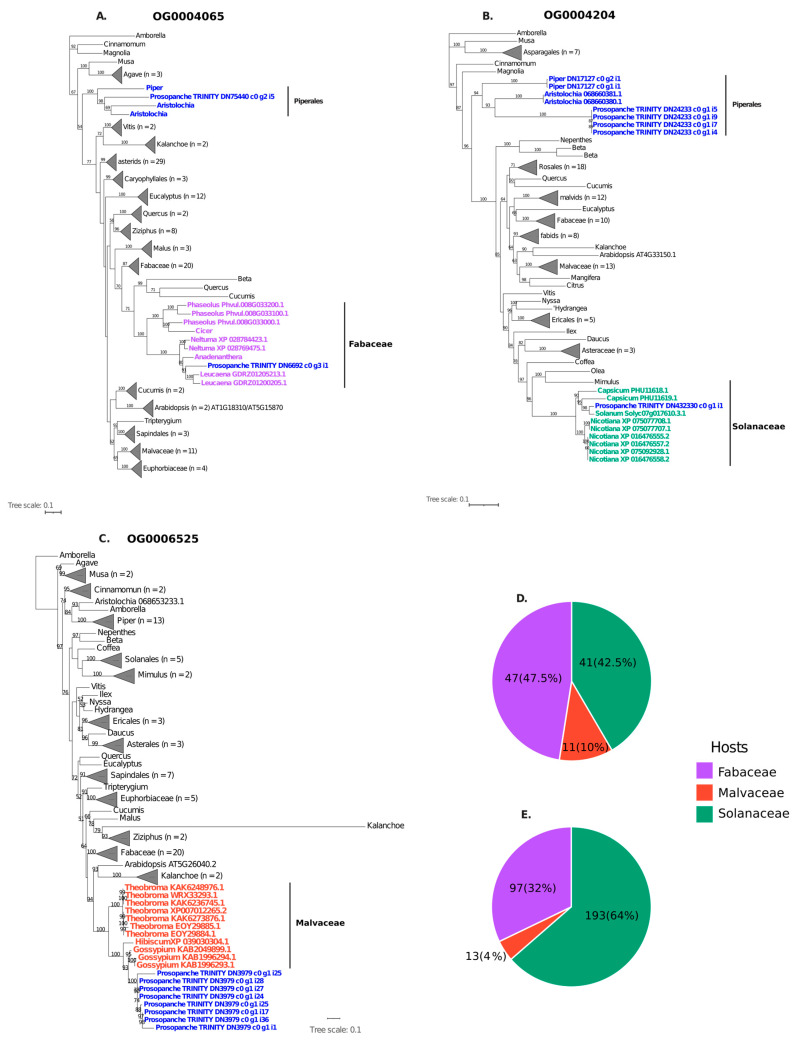
Nuclear HGT: (**A**–**C**) Maximum likelihood phylogenetic trees for three OG demonstrating evidence of Horizontal Gene Transfer (HGT) in *Prosopanche americana* from host lineages belonging to Fabaceae, Solanaceae, and Malvaceae, respectively. Trees were reconstructed using IQ-TREE under the Q.plant substitution model based on amino acid sequences. In each tree, Piperales (the order to which *Prosopanche* belongs) is shown in blue, while donor orders follow a consistent color scheme: lilac for Fabaceae, green for Solanaceae, and orange for Malvaceae. Bootstrap support values are indicated on the branches. (**D**,**E**) are pie charts summarizing the proportion of HGT-derived sequences corresponding to the three most represented host orders: (**D**) shows the percentage of OG containing at least one foreign sequence, and (**E**) shows the percentage of total foreign sequences.

To identify potential HGT candidates, the curated *Prosopanche* transcriptome was analyzed in a comparative framework alongside 45 Viridiplantae species (Table S4), leading to the inference of 101,329 OGs. We identified candidate OGs in which *Prosopanche* sequences exhibited a BLAST ‘best-hit’ to species within Fabaceae (651 OGs), Malvaceae (676 OGs), or Solanaceae (370 OGs). These groups defined the primary search space for subsequent phylogenetic validation. Following rigorous phylogenetic analyses (see Materials and Methods), we ultimately identified 99 OG exhibiting robust evidence of HGT from the three analyzed donor lineages, comprising a total of 303 horizontally acquired transcripts with bootstrap support higher than 95% (Table S6). Fabaceae was the most represented donor lineage in terms of OG (46 OGs), followed by Solanaceae (41 OGs) and Malvaceae (11 OGs) (Figure 3). However, when accounting for the total number of individual sequences, Solanaceae exhibited a marked increase in relative numerical contribution, potentially due to lineage-specific duplications following the HGT event. This indicates that Solanaceae-derived OGs tend to contain a higher number of sequences with affinity to Solanaceae in *Prosopanche* compared to those from other donors (Figure 3).

### 3.4. Functional Analyses of Foreign Nuclear Transcripts

Of the 303 foreign genes detected in the *P. americana* transcriptome, 111 were successfully annotated using one of the four methods employed, namely String, EggNOG, Mercator, or the *Arabidopsis* TAIR code (Table S6). Within the annotated fraction, Fisher’s exact test identified significantly overrepresented Gene Ontology (GO) terms (FDR < 0.05) across various categories (Table S7). Regarding Biological Processes (BP), the highest fold enrichment values (>40) were observed for terms related to specific metabolic pathways, including “L-serine metabolic process” and “one-carbon metabolic process”. Terms associated with “photosynthesis, light harvesting,” and “organic acid biosynthetic process” were also significantly overrepresented.

In terms of Cellular Components (CC), the analysis showed a significant enrichment for categories associated with intercellular connections, specifically “plasmodesma” and “cell–cell junction.” Additionally, terms related to plastid structure, such as “plastid envelope,” were identified as significantly enriched in the HGT gene set.

Functional classification via Mercator revealed a striking correlation between the function of transferred genes and their phylogenetic origin (Figure S6). We observed a distinct segregation in the “Photosynthesis” category (Mercator Bin 1). Remarkably, all sequences associated with photosynthetic light reactions (e.g., Photosystems, LHCs) were exclusively derived from Solanales, an ancestral host lineage (Figure S6, Bin 1). Conversely, the current host lineage (Fabales) contributed no genes to this category. The phylogenetic affinity of photosynthesis-related sequences (Mercator Bin 1) with Solanales—an ancestral host lineage—and not with *Neltuma* (Fabaceae)—the actual host—provides strong evidence against host tissue contamination during RNA extraction. The complete absence of Fabales-derived photosynthetic sequences, in contrast to the robust presence of Solanales-derived sequences, confirms that these are endogenous genomic insertions acquired before the lineage’s host shift.

## 4. Discussion

### 4.1. Molecular Fossils of an Ancestral Generalist in the mtDNA and in the Nucleus of Prosopanche

The HGT landscape of *P. americana* presents a paradox: while it is currently a strict specialist of Fabaceae [17], its genome acts as a reservoir of historical ecological interactions. We identified a complex mosaic of foreign sequences from Solanales, Malvales, Malpighiales, and Caryophyllales—lineages absent from its modern host range. The presence of 13 shared mitochondrial regions between *P. americana* and *P. panguanensis*, recovered as sister lineages in our phylogenies, confirms that these are not recent events or artifacts. Regarding the specific contig showing exclusive affinity to Malvaceae, we rule out contamination based on three lines of evidence: (i) ecological: Malvaceae is not a host, providing no plausible source for sample contamination; (ii) evolutionary: the ~95% sequence identity and structural recombination indicate significant divergence, inconsistent with raw environmental contamination; and (iii) phylogenetic: we identified a homologous portion of this recombined fragment in a sister species (*P. panguanensis)*, confirming an ancestral integration event rather than a sample-specific artifact. Instead, they represent ancestral HGT events that predated their speciation.

Our results suggest that the *Prosopanche* ancestor was a generalist, similar to the extant *P. bonacinae*, and that many of those hosts donated both mitochondrial and nuclear DNA. The use of HGT to identify previously unknown host relationships has been documented in other holoparasites [10,35,50]. In the mtDNA of the holoparasite *Ombrophytum subterraneum* (Balanophoraceae, Santalales), HGT analysis unveiled historical interactions with hosts (e.g., Lamiaceae, Apocynaceae) not currently parasitized by the species [35]. Interestingly, the largest foreign inserts in our study originate from Solanales and Malvales, suggesting that these now-lost interactions were once central to the parasite’s biology, before the lineage narrowed its host range toward Fabaceae.

The foreign DNA was captured before the speciation event and the subsequent ecological shift toward host specialization in the *P. americana* lineage. Current interactions with Fabaceae have left surprisingly small mitochondrial genomic footprints (~8.5 kb in *P. americana*) compared to the larger tracts retained from ancestral hosts. The presence of foreign regions from different donors and the minimal impact of HGT from the current Fabaceae donor in *P. americana* suggest two scenarios. First, the transition from a generalist to a specialist (the “ecological shift”) might be a relatively recent event in the *Prosopanche* radiation. Alternatively, the lineage may have experienced a peak of genomic plasticity followed by a cessation of horizontal acquisitions after the speciation event and host specialization. Comparing mitochondrial HGT across *Prosopanche* species with varying host ranges (generalists vs. specialists) will help determine if host specialization limits foreign DNA acquisition.

### 4.2. Comparative HGT Dynamics and Functional Landscape

Our results reinforce the idea that the transition to holoparasitism does not uniformly entail the massive acquisition of foreign DNA into the mtDNA. While some Balanophoraceae, like *Lophophytum,* act as a reservoir of host genes [5,51,52], its relative *Rhopalocnemis* exhibits virtually no organellar HGT [15]. In *Prosopanche*, we observe a distinctive scenario. Despite its ancient Cretaceous origin—which theoretically offered ample time for accumulation—the mitochondrial genome exhibits remarkably low levels of HGT. This disparity suggests that lineage-specific intrinsic factors differ even among closely related *Prosopanche* species. While *P. americana* shows a restricted uptake or retention of mitochondrial DNA from its current host (Fabales, ~8.5 kb), its sister species *P. panguanensis* displays a comparatively higher retention of Fabales-derived sequences (~44 kb).

### 4.3. Functional Bias in Nuclear Retained Sequences

Regarding the functional role of these foreign genes, although functional characterization was limited by annotation availability (~40% of transferred genes), the retained foreign genes do not reflect metabolic adaptation. The enrichment in plastid-related terms (e.g., thylakoids, photosystems) is intriguing in light of the extensive convergent loss of photosynthetic genes documented in other holoparasitic plants [13,53,54]. In a non-photosynthetic parasite, this does not imply a functional “rescue” of metabolic activity. In contrast to findings in other holoparasitic lineages where HGT has been linked to the acquisition of adaptive traits [8,55], our results likely reflect the donor’s genomic architecture. Since these genes are often found in high-copy numbers in the donor’s organellar or nuclear genomes, they may simply be more likely to be captured. The retention of these sequences might be a byproduct of neutral genomic integration rather than an adaptive requirement for the holoparasitic lifestyle [56]. However, given that these genes could potentially be functionally co-opted, this hypothesis remains to be tested. Future analyses of molecular evolutionary rates and pseudogenization signatures, coupled with expression assays, are required to test whether these foreign inserts are undergoing neutral genomic decay or have been functionally repurposed.

## Data Availability

The genomic data generated for this study have been deposited in NCBI: Illumina DNAseq (SRR37801949).

## Supplementary Materials

The following supporting information can be downloaded at: https://www.mdpi.com/article/10.3390/plants15071121/s1, Table S1. GenBank accession numbers of the *Prosopanche* spp. data sets. Table S2. List of NCBI mitochondrial sequences (>4 kb) included in the custom reference databases for BLASTn searches. Table S3. BUSCO completeness assessment of the *Prosopanche* transcriptome assembly based on assembled transcripts (A) and predicted proteins (B). Table S4. Species included in the OrthoFinder analyses. Table S5. Inventory of mitochondrial foreign sequences in *Prosopanche americana* and assessment of their presence in the sister species *P. panguanensis*. Table S6. Functional annotation and classification of *Prosopanche* transcripts associated with different host plant families. Table S7. Gene Ontology (GO) enrichment analysis of horizontally transferred transcripts identified in the *Prosopanche americana* transcriptome. Figure S1. Bioinformatic workflow for the identification and validation of Horizontal Gene Transfer (HGT) events in the nuclear transcriptome of *Prosopanche americana*. The pipeline follows four sequential phases: Phase 1 (Quality Control): The predicted proteome was filtered to remove transposable elements (TEs) using HMMER and non-Viridiplantae contaminants via eggNOG taxonomic annotation. Phase 2 (Orthology): OG data were inferred using OrthoFinder, including *Prosopanche*, potential host lineages (Fabaceae, Solanaceae, and Malvaceae), and outgroups. Preliminary candidates were selected based on BLASTp best-hit criteria against host families. Phase 3 (Phylogenomics): Candidates underwent rigorous phylogenetic validation. Sequences were aligned (MAFFT) and trimmed (ClipKIT) before maximum likelihood tree reconstruction (IQ-TREE). Trees were topologically filtered to retain only those supporting a robust parasite–host clustering. Phase 4 (Curation): The final set of HGT candidates was curated by removing redundancy (CD-HIT) and discarding remaining sequences functionally annotated as TE machinery. Numbers in parentheses indicate the count of sequences or candidates retained at key steps. Figure S2. Visualization of BLASTn homology searches for the *Prosopanche americana* mitochondrial contigs. BLASTn hits between *Prosopanche* and a custom angiosperm mitochondrial database are organized by taxonomic order, as described in Hatt et al. (2025) [17]. The *Y*-axis represents different angiosperm lineages, including potential hosts (e.g., Solanales, Fabales, Malvales) and other reference groups. Colors indicate percentage identity (pident), ranging from blue (lower identity) to red (high identity). Gray arrows below the tracks indicate the position and transcriptional direction of annotated mitochondrial genes. Note that vertical “stacks” of hits across multiple orders typically represent conserved coding regions (e.g., *atp1*, *ccmB*), while high-identity hits restricted to specific lineages suggest Horizontal Gene Transfer (HGT) events. Figure S3. Maximum likelihood (ML) phylogenetic analyses of mitochondrial foreign regions shared by taxa beyond the parasite and the host. Phylogenetic trees were reconstructed from nucleotide alignments under the GTR + G substitution model. Numbers at nodes indicate bootstrap support values. Clades corresponding to Solanales, Malpighiales, and Fabales are highlighted in green, pink, and purple, respectively. The tree identifiers correspond to the specific mitochondrial contigs as designated in Yu et al. [24], including their respective genomic coordinates. Figure S4. Horizontal Gene Transfer (HGT) landscape in the *Prosopanche panguanensis* mtDNA. Each horizontal bar represents a single contig, with the *X*-axis showing the position in kilobases (kb). The BLAST analysis was conducted against custom databases, including host orders, the order Piperales, and all other angiosperms. Colored segments represent regions identified as high-confidence HGT candidates, where the *P. panguanensis* sequence showed its best BLAST hit against a specific host family (see legend). These hits met the filtering criteria: p-identity > 90 and length > 200 bp. The color key below the graph indicates the specific host family that provided the best hit for the HGT region. The light gray background of the contigs represents regions that did not meet the HGT criteria. This includes sequences that did not yield a significant BLAST hit, hits broadly against all angiosperms (e.g., “Other Angiosperms” in the full database), and hits only against the native order Piperales. Note that only contigs containing identified HGT regions are displayed; entirely native contigs are excluded from the figure. Figure S5. Maximum likelihood (ML) phylogenetic analyses of mitochondrial genes identified in the draft mitochondrial assemblies of *P. bonacinae* and *P. panguanensis*. Phylogenetic trees were reconstructed from nucleotide alignments under the GTR + G substitution model. For ease of identification, species belonging to the order Piperales (including the genus *Prosopanche*) are highlighted in red. For genes where only partial sequences were recovered, this status is indicated following the taxon name. These trees illustrate the phylogenetic placement of mitochondrial loci identified within the genomic assemblies of three holoparasitic species. Figure S6. Functional landscape of horizontally transferred genes in the *Prosopanche americana* nuclear genome. HGT candidates were annotated using the Mercator pipeline and grouped by biological pathway (*Y*-axis) across the three main donor lineages (*X*-axis). The size of each circle is proportional to the number of transcripts assigned to each category. Note the specific enrichment of photosynthesis-related genes (e.g., photosystem subunits, light harvesting) derived exclusively from Solanaceae, in contrast to their absence in the current host lineage (Fabaceae).
