# Supplementary material for: Genomic Footprints of Multiple Host Lineages in the Mitochondrial and Nuclear Genomes of the Holoparasite Prosopanche americana
Source: Plants (Basel). 2026 Apr 7;15(7):1121. doi: 10.3390/plants15071121 (PMC13074556; doi:10.3390/plants15071121)

Figure S1. Bioinformatic workflow for the identification and validation of Horizontal Gene Transfer (HGT) events in the nuclear transcriptome of *Prosopanche americana*. The pipeline follows four sequential phases. Phase 1 (Quality Control): The predicted proteome was filtered to remove transposable elements (TEs) using HMMER and non-Viridiplantae contaminants via eggNOG taxonomic annotation. Phase 2 (Orthology): OG were inferred using OrthoFinder, including Prosopanche, potential host lineages (Fabaceae, Solanaceae, Malvaceae), and outgroups. Preliminary candidates were selected based on BLASTP best-hit criteria against host families. Phase 3 (Phylogenomics): Candidates underwent rigorous phylogenetic validation. Sequences were aligned (MAFFT) and trimmed (ClipKIT) before Maximum Likelihood tree reconstruction (IQ-TREE). Trees were topologically filtered to retain only those supporting a robust Parasite-Host clustering. Phase 4 (Curation): The final set of HGT candidates was curated by removing redundancy (CD-HIT) and discarding remaining sequences functionally annotated as TE machinery. Numbers in parentheses indicate the count of sequences or candidates retained at key steps.

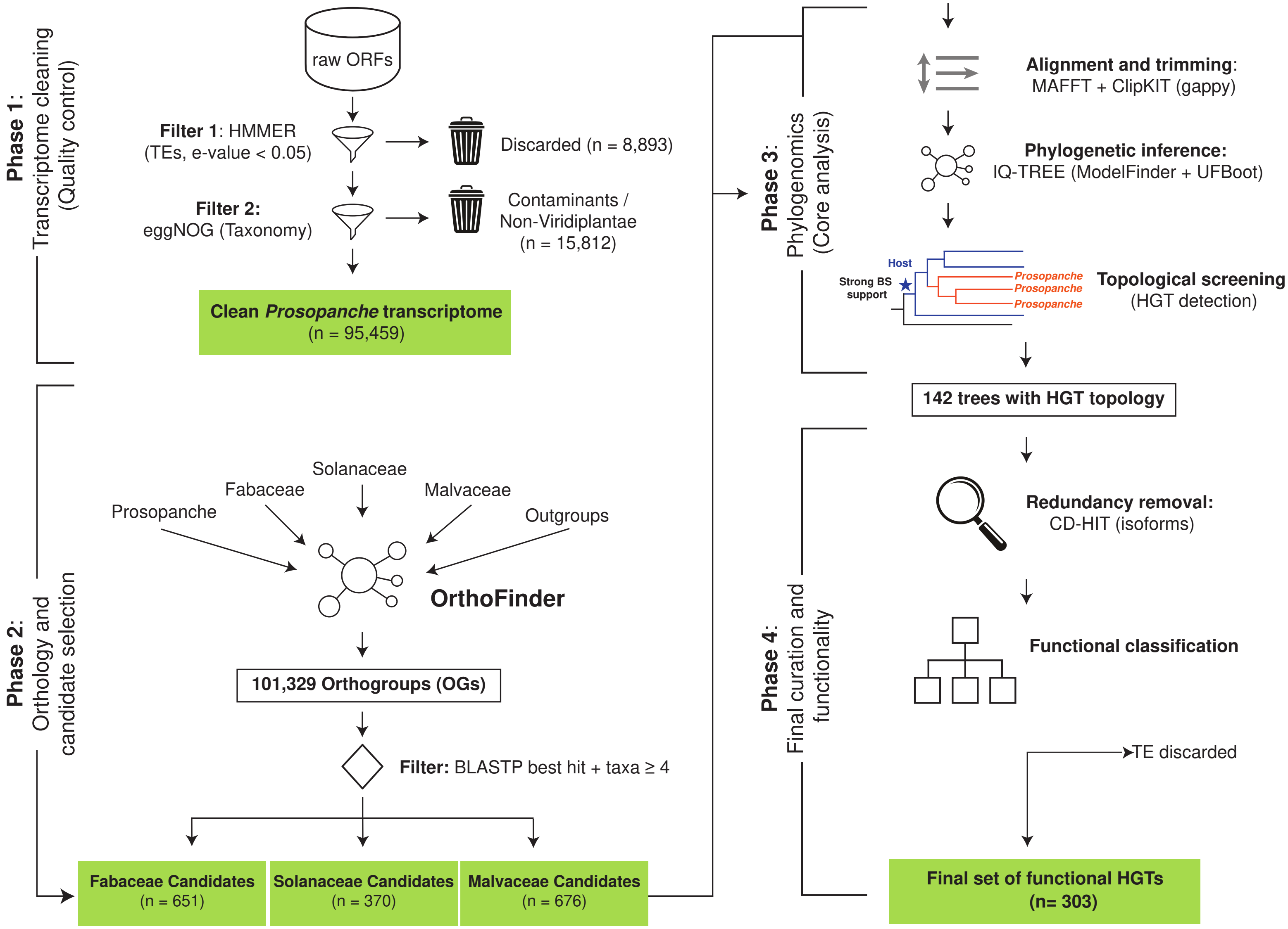

Supplement: Supplementary file 1 [file plants-15-01121-s001.zip › FigureS1.pdf]
