# Supplementary material for: Genomic Footprints of Multiple Host Lineages in the Mitochondrial and Nuclear Genomes of the Holoparasite Prosopanche americana
Source: Plants (Basel). 2026 Apr 7;15(7):1121. doi: 10.3390/plants15071121 (PMC13074556; doi:10.3390/plants15071121)
Supplement: Supplementary file 1 [file plants-15-01121-s001.zip › FigureS2.pdf]

Figure S2. Visualization of BLASTn homology searches for the *Prosopanche americana* mitochondrial contigs. BLASTn hits between *Prosopanche* and a custom angiosperm mitochondrial database are organized by taxonomic order, as described in Hatt et al. (2025). The Y-axis represents different angiosperm lineages, including potential hosts (e.g., Solanales, Fabales, Malvales) and other reference groups. Colors indicate percentage identity (pident), ranging from blue (lower identity) to red (high identity). Gray arrows below the tracks indicate the position and transcriptional direction of annotated mitochondrial genes. Note that vertical "stacks" of hits across multiple orders typically represent conserved coding regions (e.g., *atp1*, *ccmB*), while high-identity hits restricted to specific lineages suggest Horizontal Gene Transfer (HGT) events.

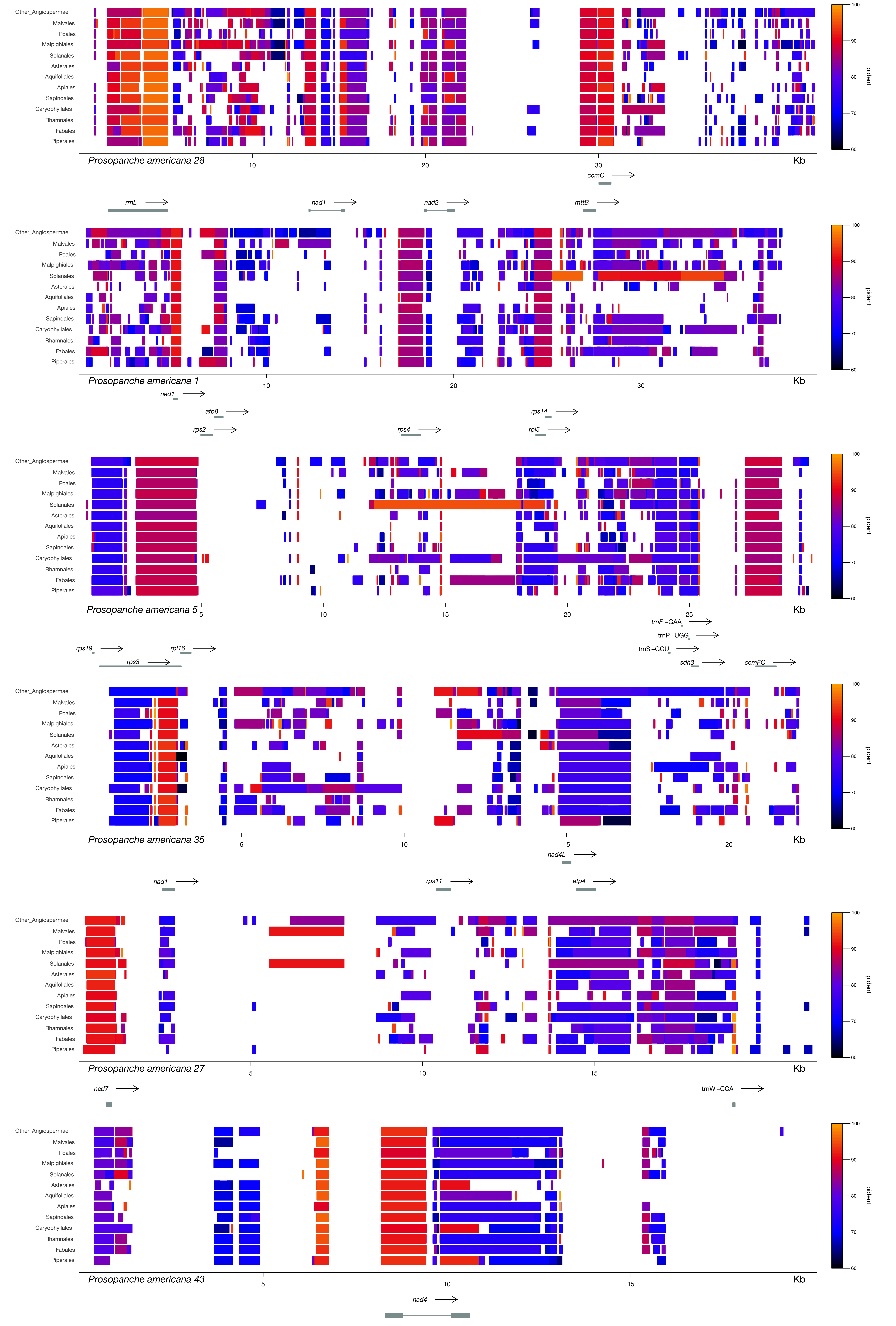

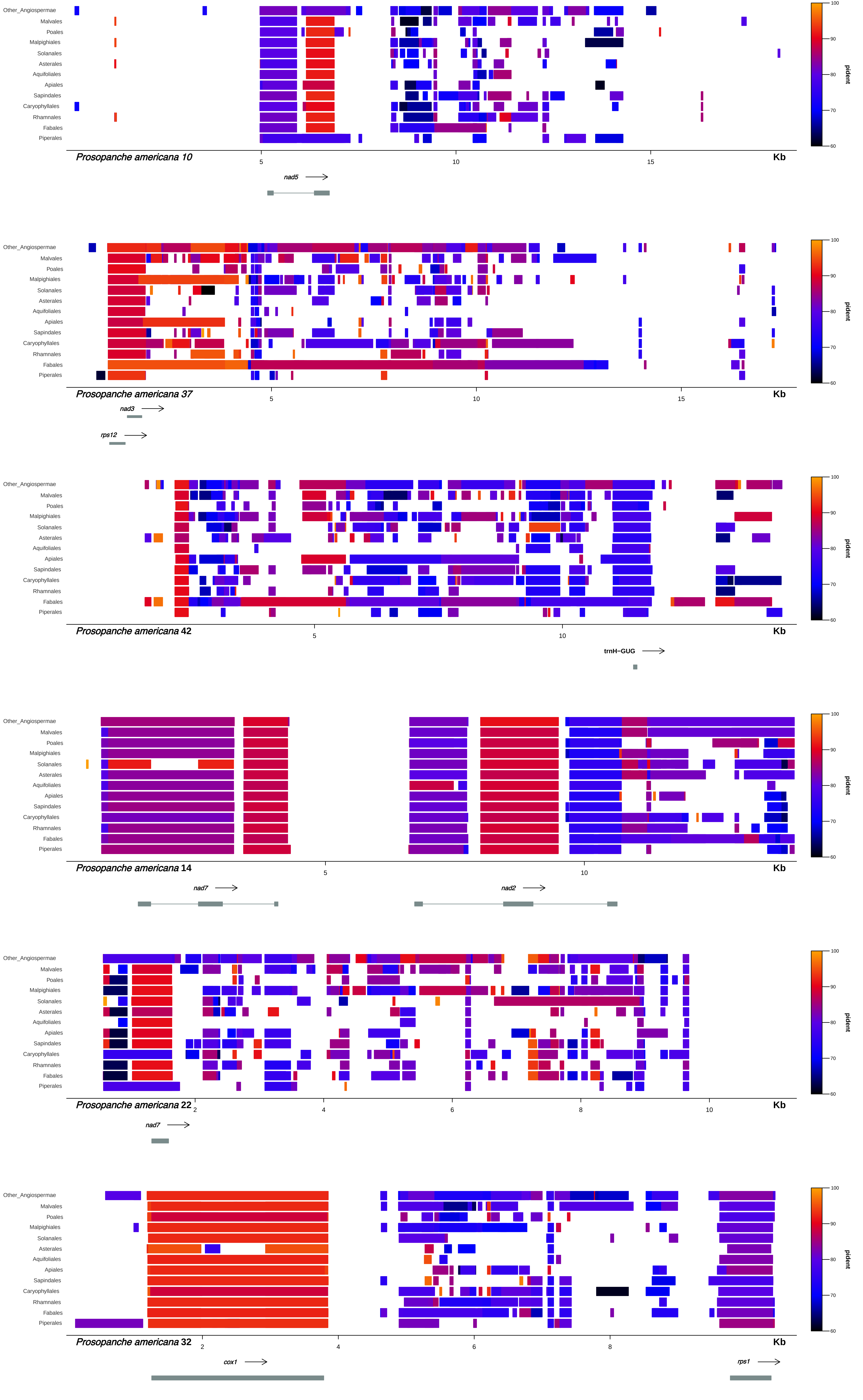
Other_Angiospermae
Malvales
Poales
Malpighiales
Solanales
Asterales
Aquifoliales
Apiales
Sapindales
Caryophyllales
Rhamnales
Fabales
Piperales
Prosopanche americana 10
5
10
15
Kb
nad5
pident
60
70
80
90
100
Prosopanche americana 37
nad3
rps12
Prosopanche americana 42
trnH−GUG
Prosopanche americana 14
nad7
nad2
Prosopanche americana 22
2
4
6
8
Prosopanche americana 32
cox1
rps1

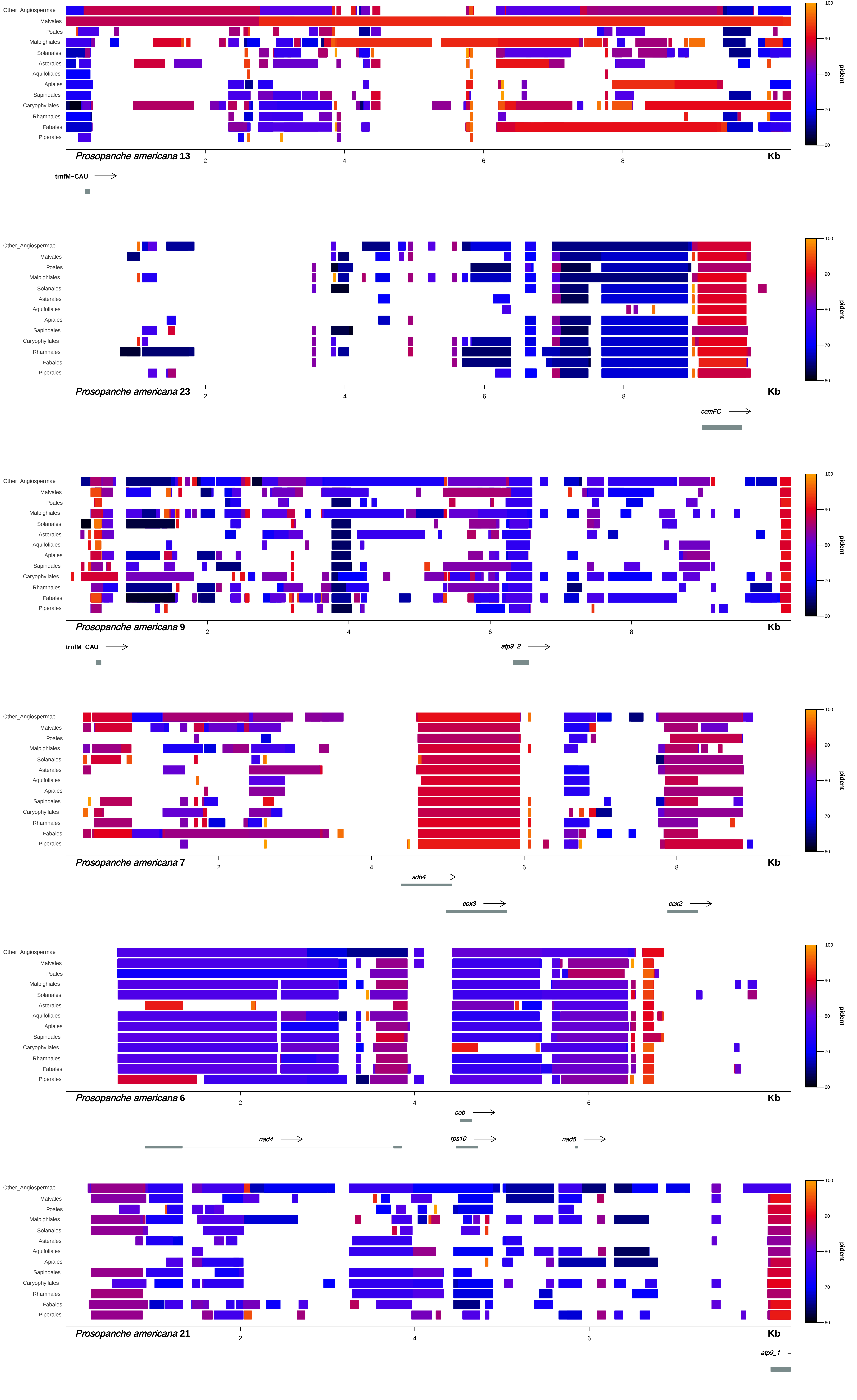

Other_Angiospermae
Malvales
Poales
Malpighiales
Solanales
Asterales
Aquifoliales
Apiales
Sapindales
Caryophyllales
Rhamnales
Fabales
Piperales
Prosopanche americana 13
2
4
6
8
Kb
trnfM−CAU
pident
100
90
80
70
60
Prosopanche americana 23
ccmFC
Prosopanche americana 9
trnfM−CAU
atp9_2
Prosopanche americana 7
sdh4
cox3
cox2
Prosopanche americana 6
cob
nad4
rps10
nad5
Prosopanche americana 21
atp9_1

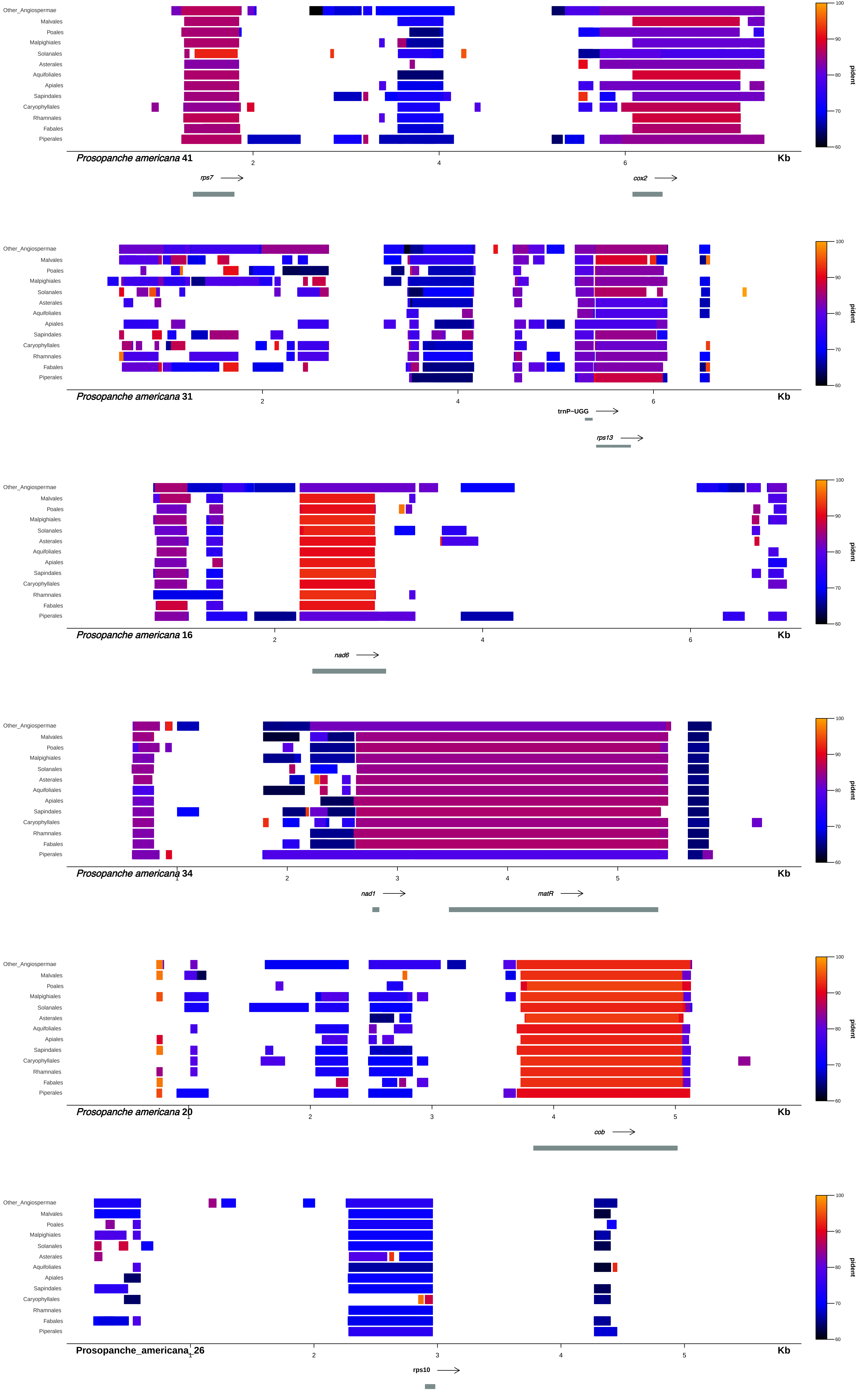
Other_Angiospermae
Malvales
Poales
Malpighiales
Solanales
Asterales
Aquifoliales
Apiales
Sapindales
Caryophyllales
Rhamnales
Fabales
Piperales
Prosopanche americana 41
2
4
6
Kb
rps7
cox2
pident
60
70
80
90
100
Prosopanche americana 31
trnP−UGG
rps13
Prosopanche americana 16
nad6
Prosopanche americana 34
1
2
3
4
5
nad1
matR
Prosopanche americana 20
cob
Prosopanche_americana_26
rps10

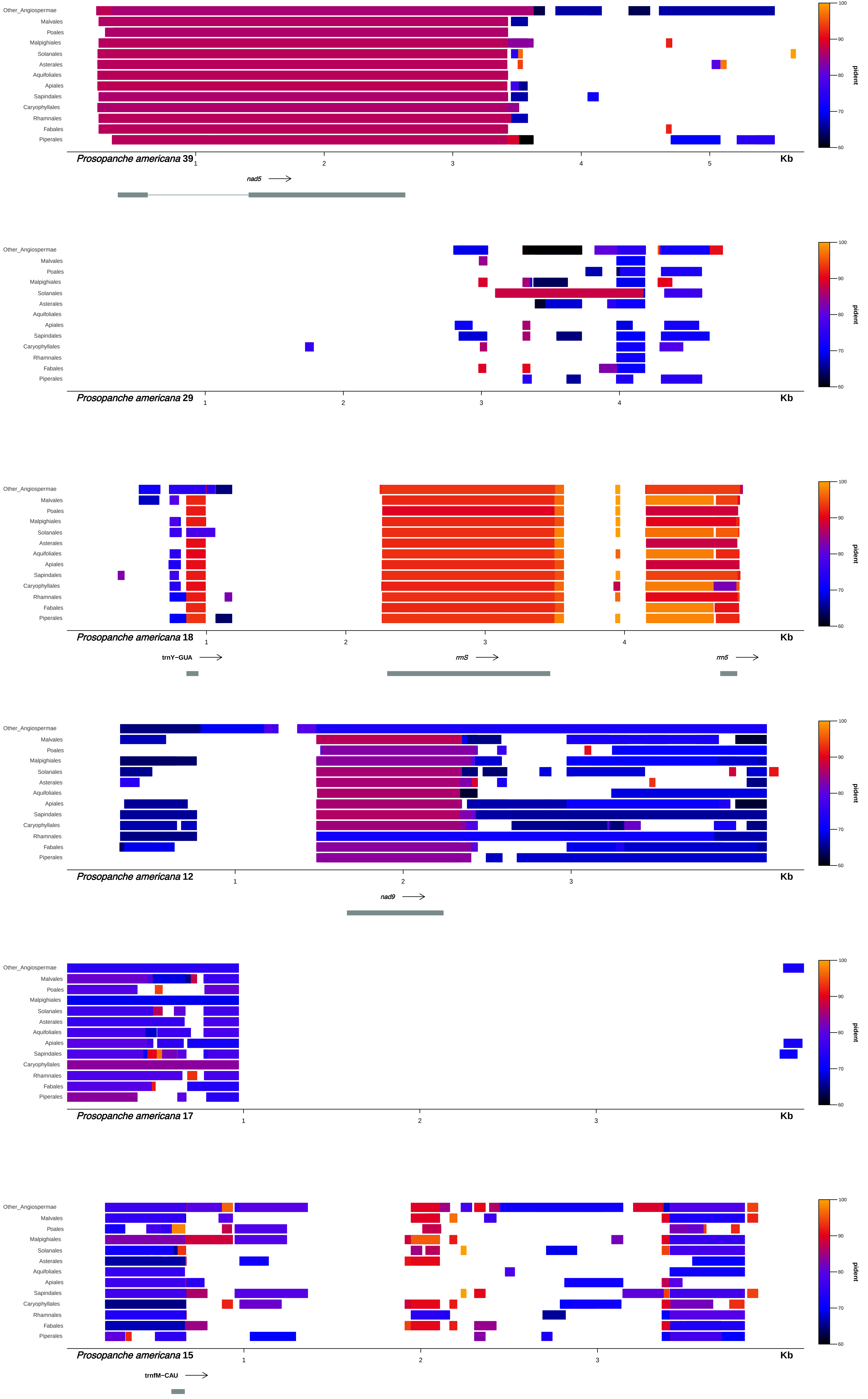
Other_Angiospermae
Malvales
Poales
Malpighiales
Solanales
Asterales
Aquifoliales
Apiales
Sapindales
Caryophyllales
Rhamnales
Fabales
Piperales
Prosopanche americana 39
1 2 3 4 5
Kb
nad5
pident
60 70 80 90 100
Prosopanche americana 29
1 2 3 4
Kb
Prosopanche americana 18
1 2 3 4
Kb
trnY−GUA
rrnS
rrn5
Prosopanche americana 12
1 2 3
Kb
nad9
Prosopanche americana 17
1 2 3
Kb
Prosopanche americana 15
1 2 3
Kb
trnfM−CAU

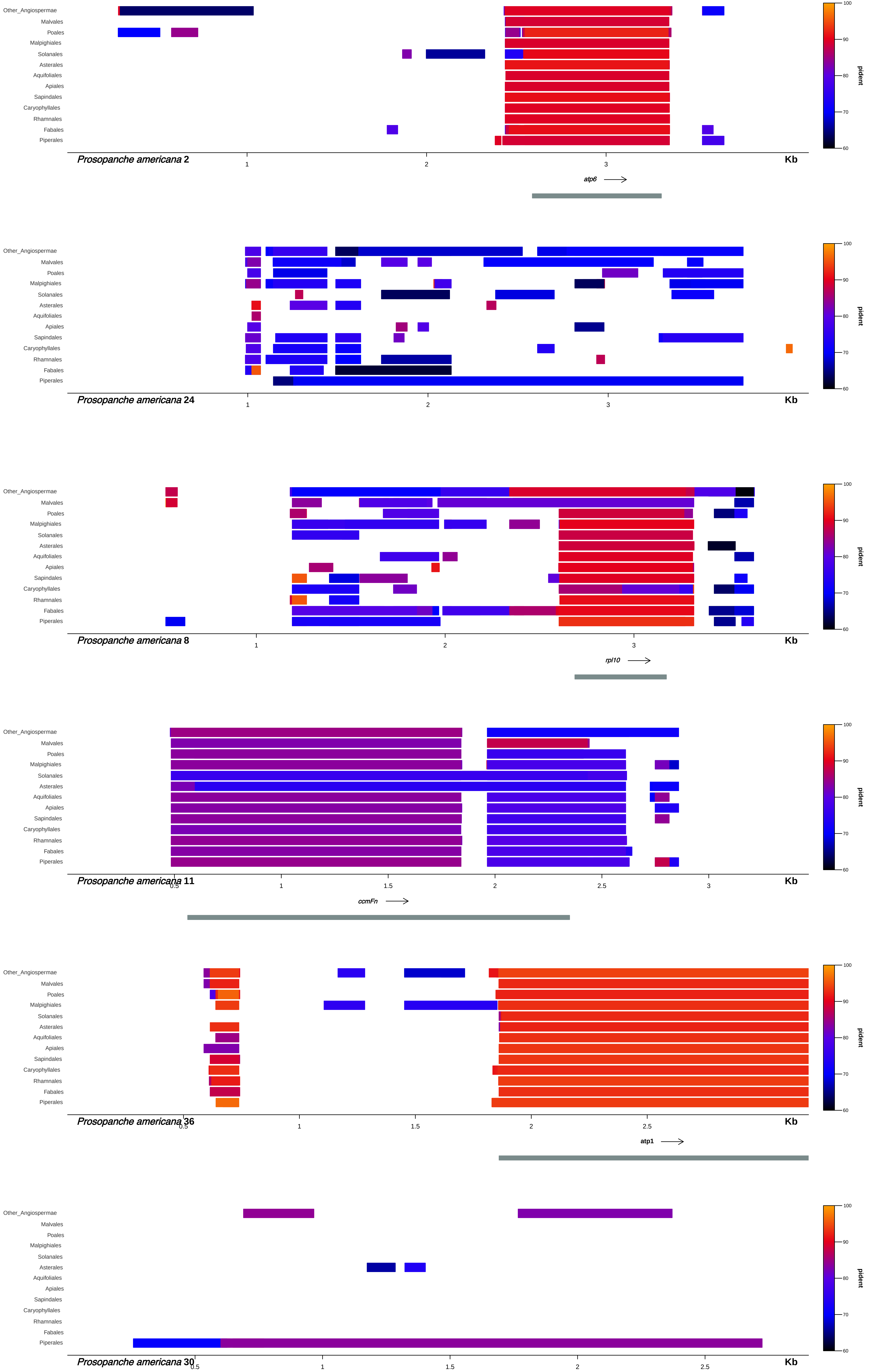
Other_Angiospermae
Malvales
Poales
Malpighiales
Solanales
Asterales
Aquifoliales
Apiales
Sapindales
Caryophyllales
Rhamnales
Fabales
Piperales
Prosopanche americana 2
atp6
Prosopanche americana 24
Prosopanche americana 8
rpl10
Prosopanche americana 11
ccmFn
Prosopanche americana 36
atp1
Prosopanche americana 30
Kb
pident
60
70
80
90
100

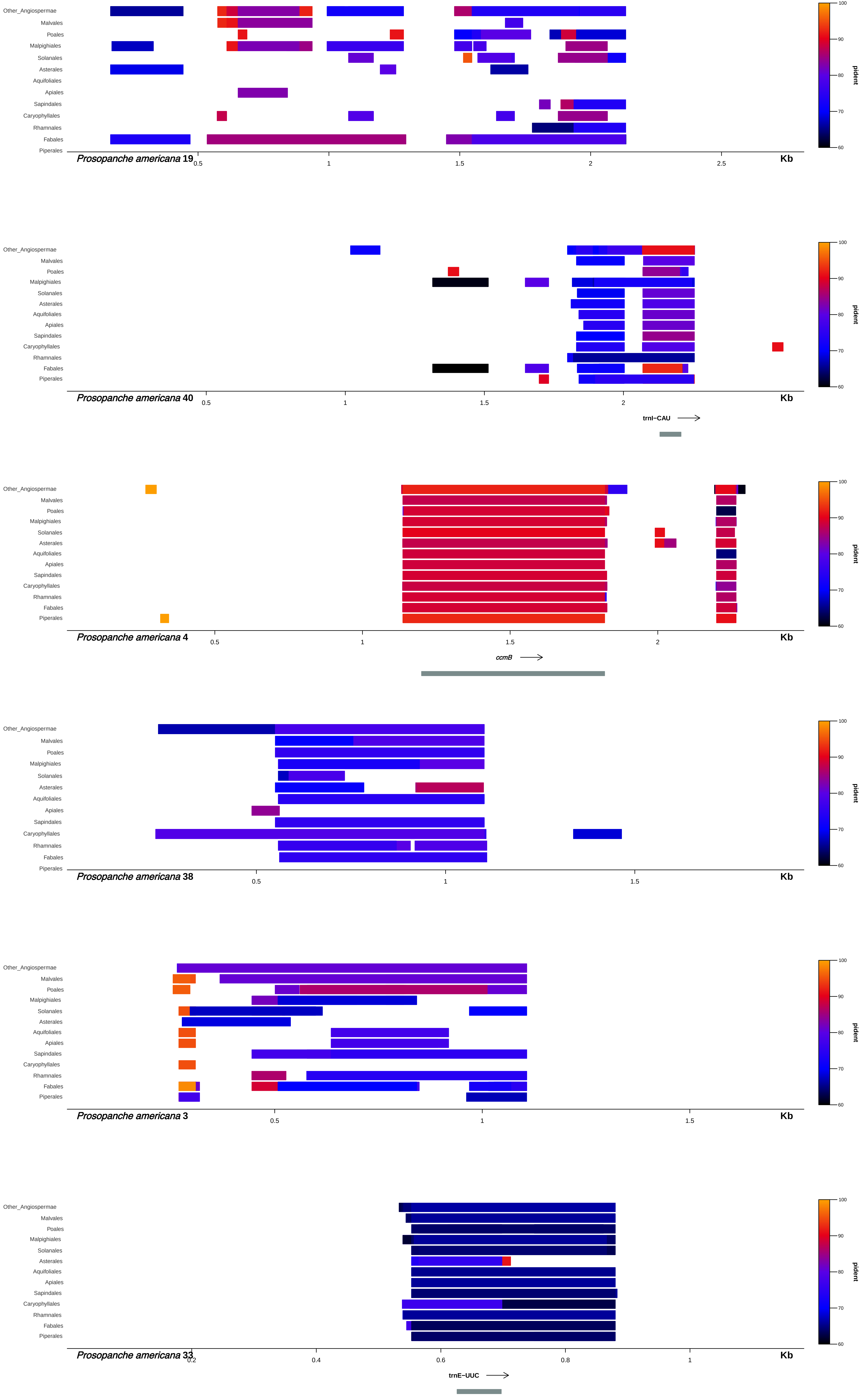
Other_Angiospermae
Malvales
Poales
Malpighiales
Solanales
Asterales
Aquifoliales
Apiales
Sapindales
Caryophyllales
Rhamnales
Fabales
Piperales
Prosopanche americana 19
Prosopanche americana 40
Prosopanche americana 4
Prosopanche americana 38
Prosopanche americana 3
Prosopanche americana 33
Kb
pident
trnI−CAU
ccmB
trnE−UUC
