# Supplementary material for: Genomic Footprints of Multiple Host Lineages in the Mitochondrial and Nuclear Genomes of the Holoparasite Prosopanche americana
Source: Plants (Basel). 2026 Apr 7;15(7):1121. doi: 10.3390/plants15071121 (PMC13074556; doi:10.3390/plants15071121)

Figure S3. Maximum likelihood (ML) phylogenetic analyses of mitochondrial foreign regions shared by taxa beyond the parasite and the host. Phylogenetic trees were reconstructed from nucleotide alignments under the GTR+G substitution model. Numbers at nodes indicate bootstrap support values. Clades corresponding to Solanales, Malpighiales, and Fabales are highlighted in green, pink and purple, respectively. The tree identifiers correspond to the specific mitochondrial contigs as designated in Yu et al. [24], including their respective genomic coordinates.

Contig1-663-1376

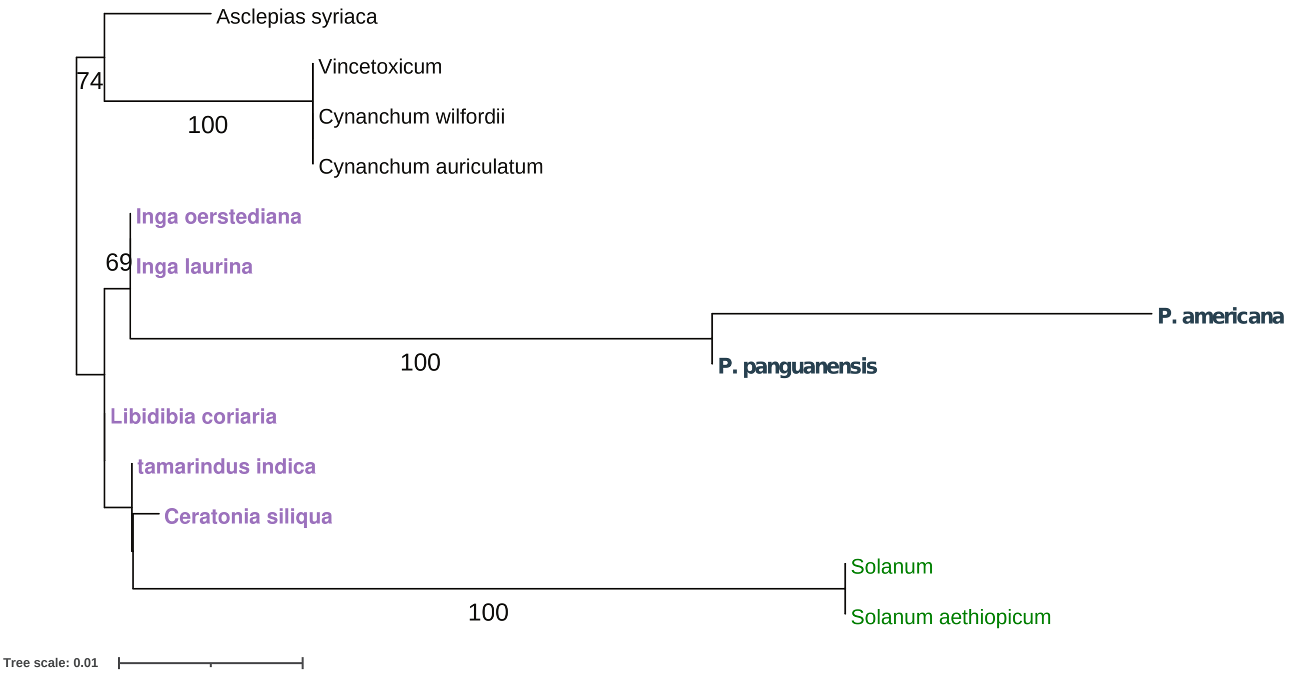

Contig1-25290-26907

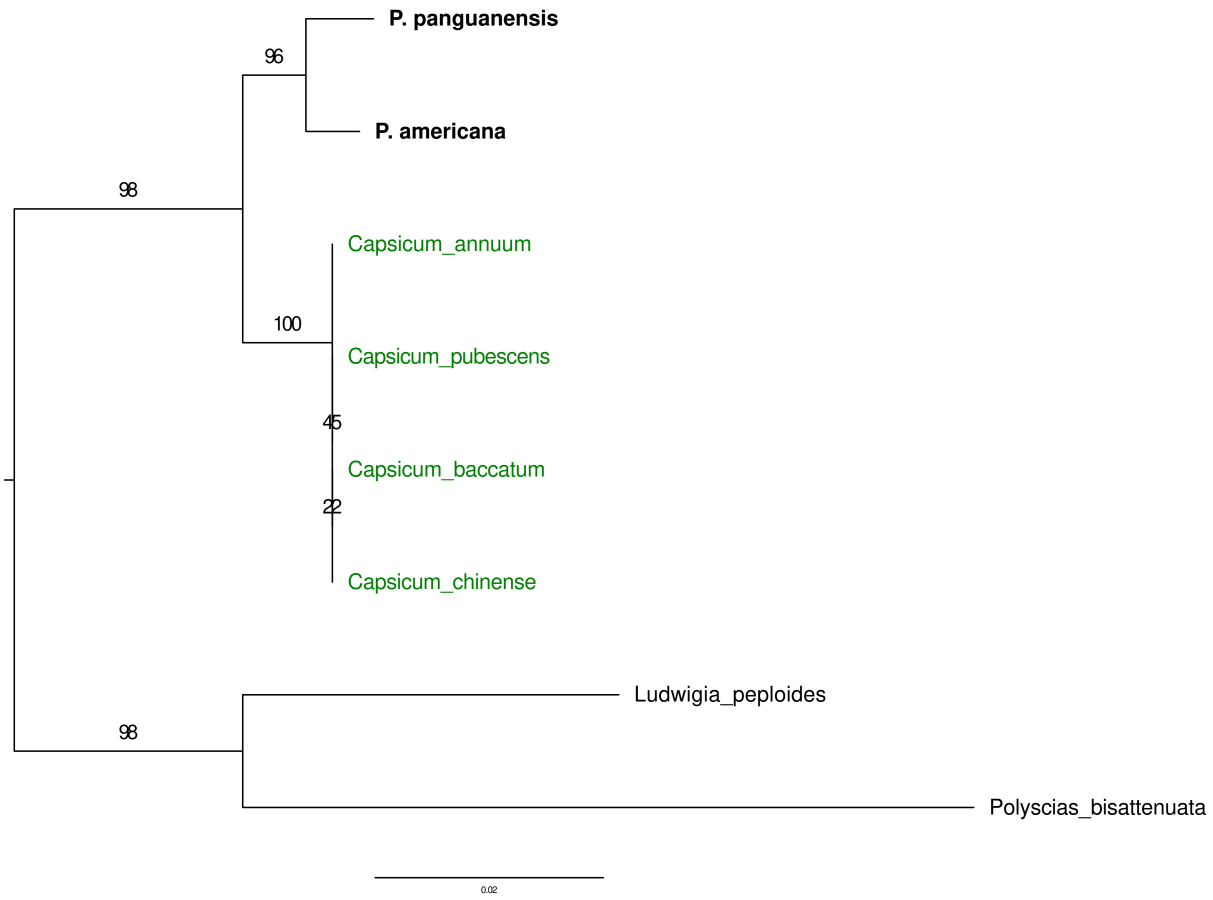

Contig1-27742-32109

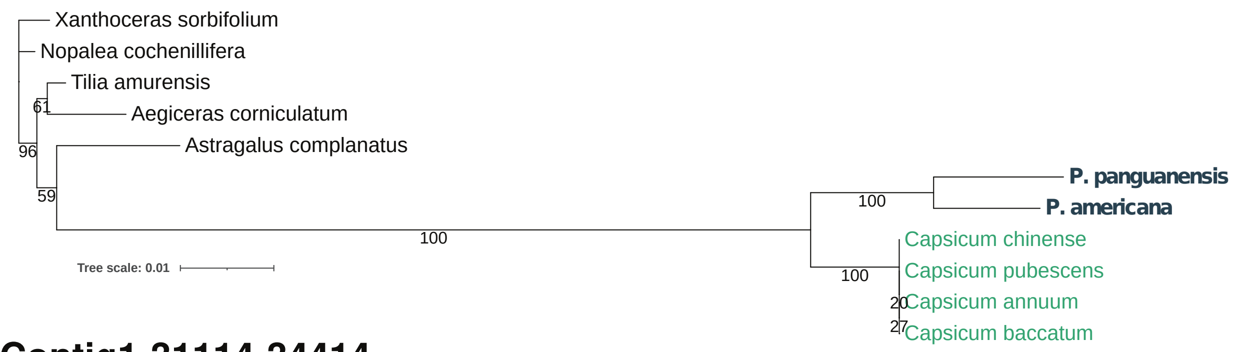

Contig1-31114-34414

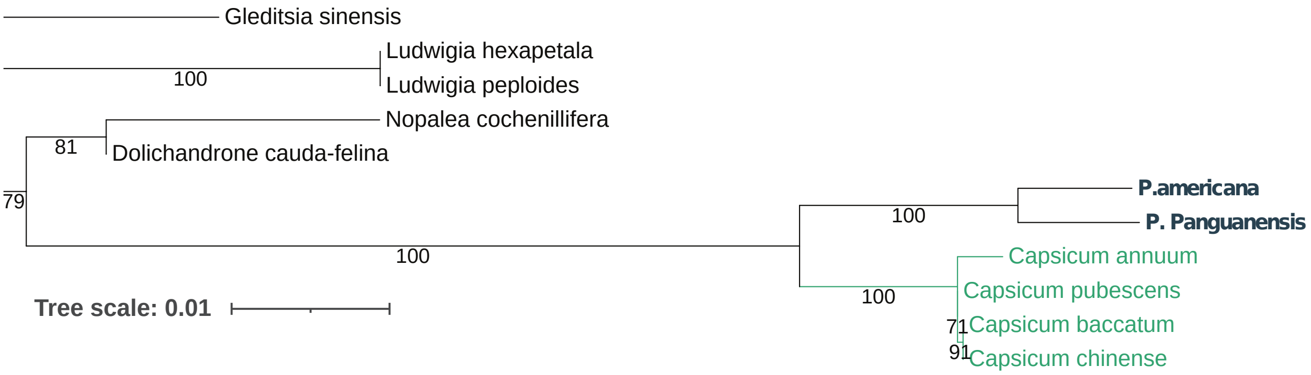

Contig5-18181-19077

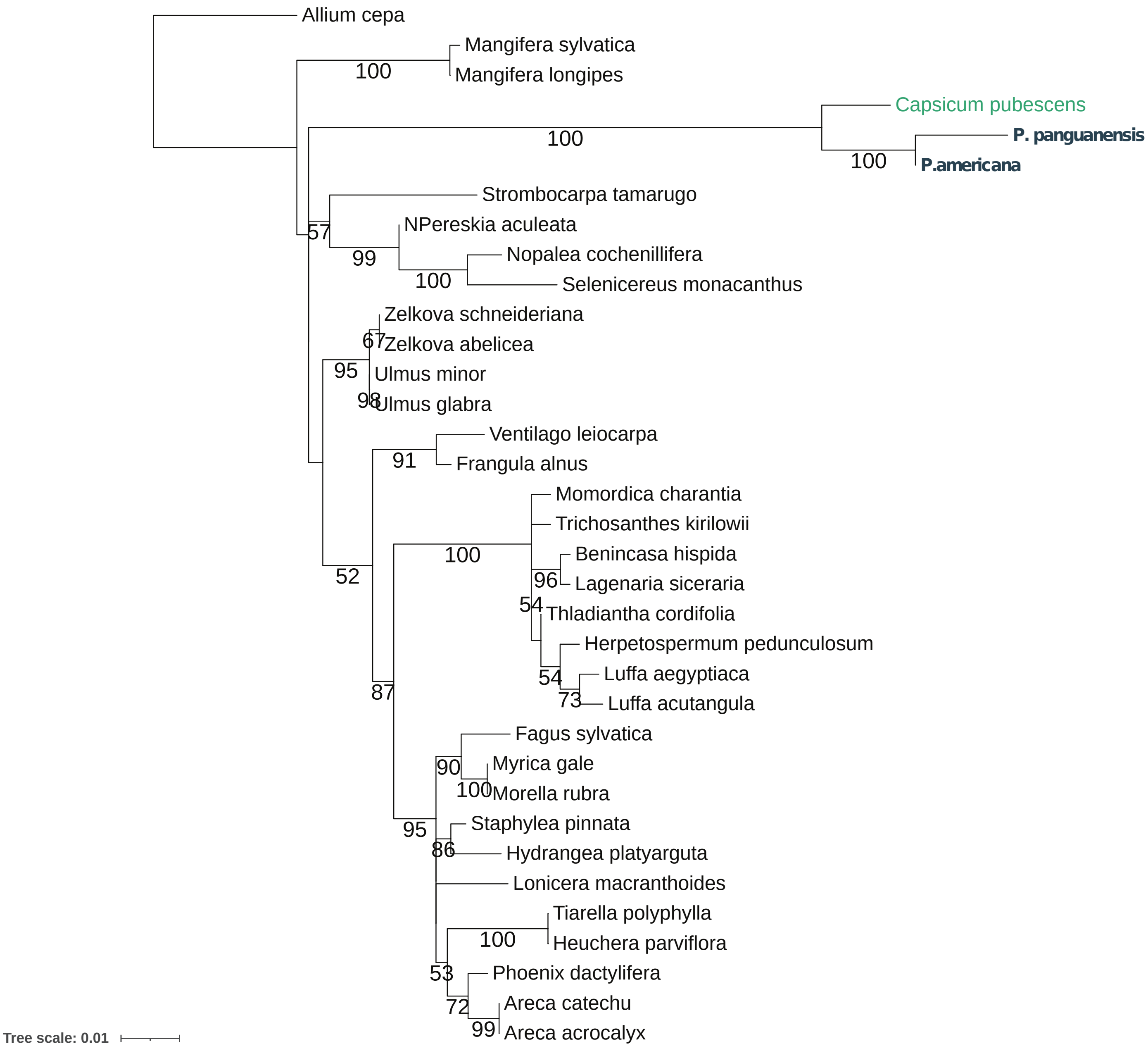

Contig22-7150-7766

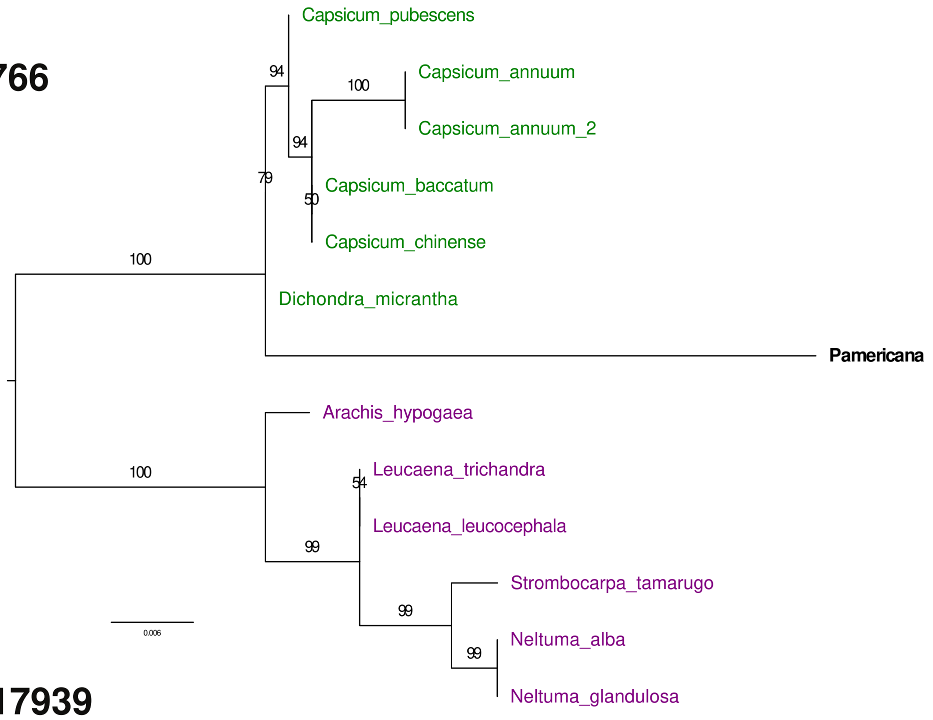

Contig27-17049-17939

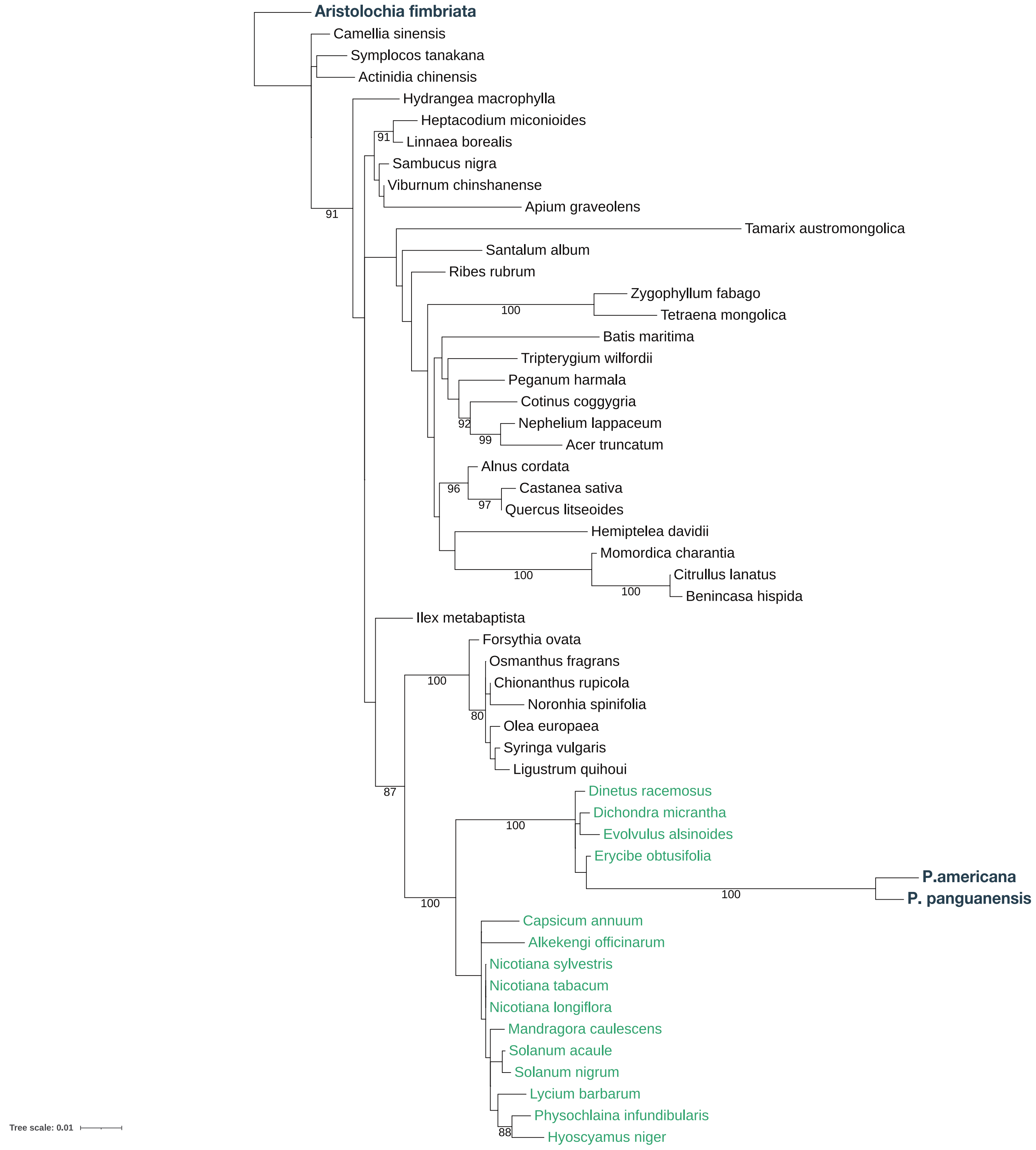

Supplement: Supplementary file 1 [file plants-15-01121-s001.zip › FigureS3.pdf]
