# Supplementary material for: Genomic Footprints of Multiple Host Lineages in the Mitochondrial and Nuclear Genomes of the Holoparasite Prosopanche americana
Source: Plants (Basel). 2026 Apr 7;15(7):1121. doi: 10.3390/plants15071121 (PMC13074556; doi:10.3390/plants15071121)

Figure S4. Horizontal Gene Transfer (HGT) landscape and host sequence coverage in the *Prosopanche panguanensis* mtDNA.
The inset donut chart displays the percentage of the total mitochondrial genome covered by sequences originating from different host lineages, based on total genomic coverage (bp). The main graph displays the mitochondrial contigs, where each horizontal bar represents a single contig, with the X-axis showing the position in kilobases (kb). The BLAST analysis was conducted against custom databases including host orders, the order Piperales, and all other angiosperms. Colored segments represent regions identified as high-confidence HGT candidates, where the *P. panguanensis* sequence showed its best BLAST hit against a specific host family (criteria: p-identity > 90 and length > 200 bp). The color key below the graph indicates the specific host Order that provided the best hit for the HGT region. The light gray background of the contigs represents regions that did not meet the HGT criteria. This includes sequences that did not yield a significant BLAST hit, hits broadly against all angiosperms (e.g., "Other Angiosperms"), and hits only against the native order Piperales. Note that only contigs containing identified HGT regions are displayed; entirely native contigs are excluded from the figure.

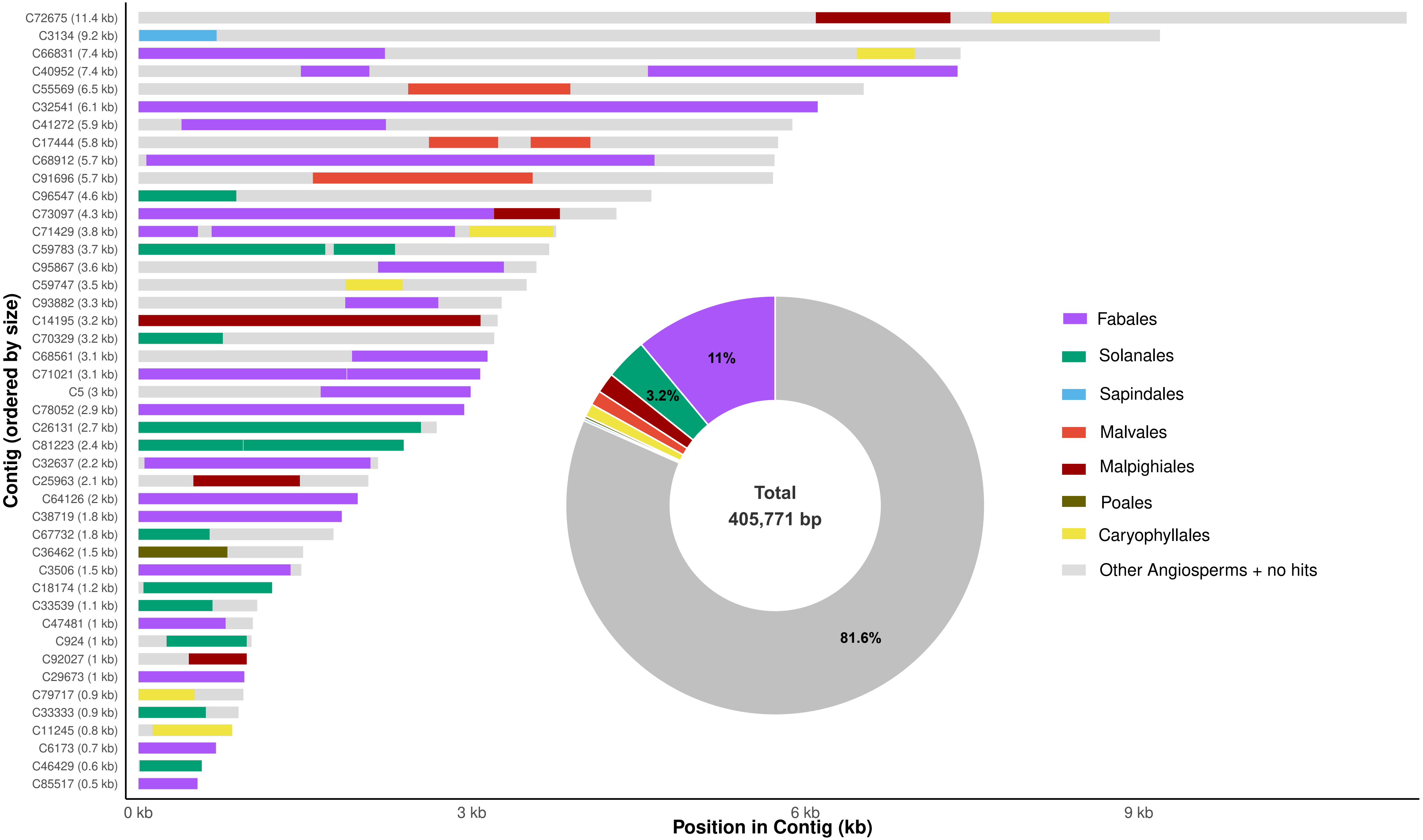

Supplement: Supplementary file 1 [file plants-15-01121-s001.zip › FigureS4.pdf]
