# Supplementary material for: Genomic Footprints of Multiple Host Lineages in the Mitochondrial and Nuclear Genomes of the Holoparasite Prosopanche americana
Source: Plants (Basel). 2026 Apr 7;15(7):1121. doi: 10.3390/plants15071121 (PMC13074556; doi:10.3390/plants15071121)
Supplement: Supplementary file 1 [file plants-15-01121-s001.zip › FigureS5.pdf]

Figure S5: Maximum likelihood (ML) phylogenetic analyses of mitochondrial genes identified in the draft mitochondrial assemblies of *P. bonacinae* and *P. panguanensis*. Phylogenetic trees were reconstructed from nucleotide alignments under the GTR+G substitution model. For ease of identification, species belonging to the order Piperales (including the genus *Prosopanche*) are highlighted in red. For genes where only partial sequences were recovered, this status is indicated following the taxon name. These trees illustrate the phylogenetic placement of mitochondrial loci identified within the genomic assemblies of three holoparasitic species.

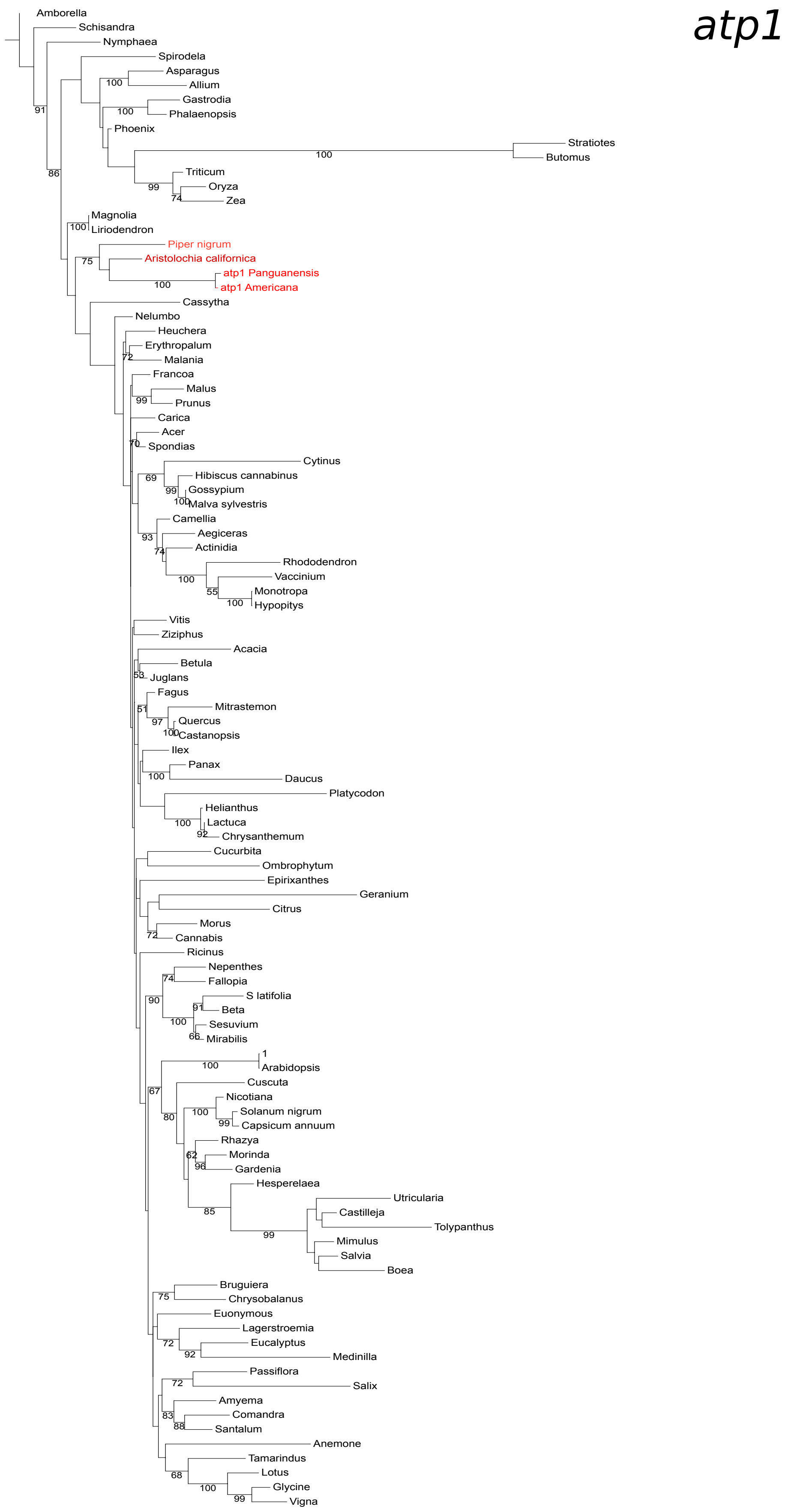

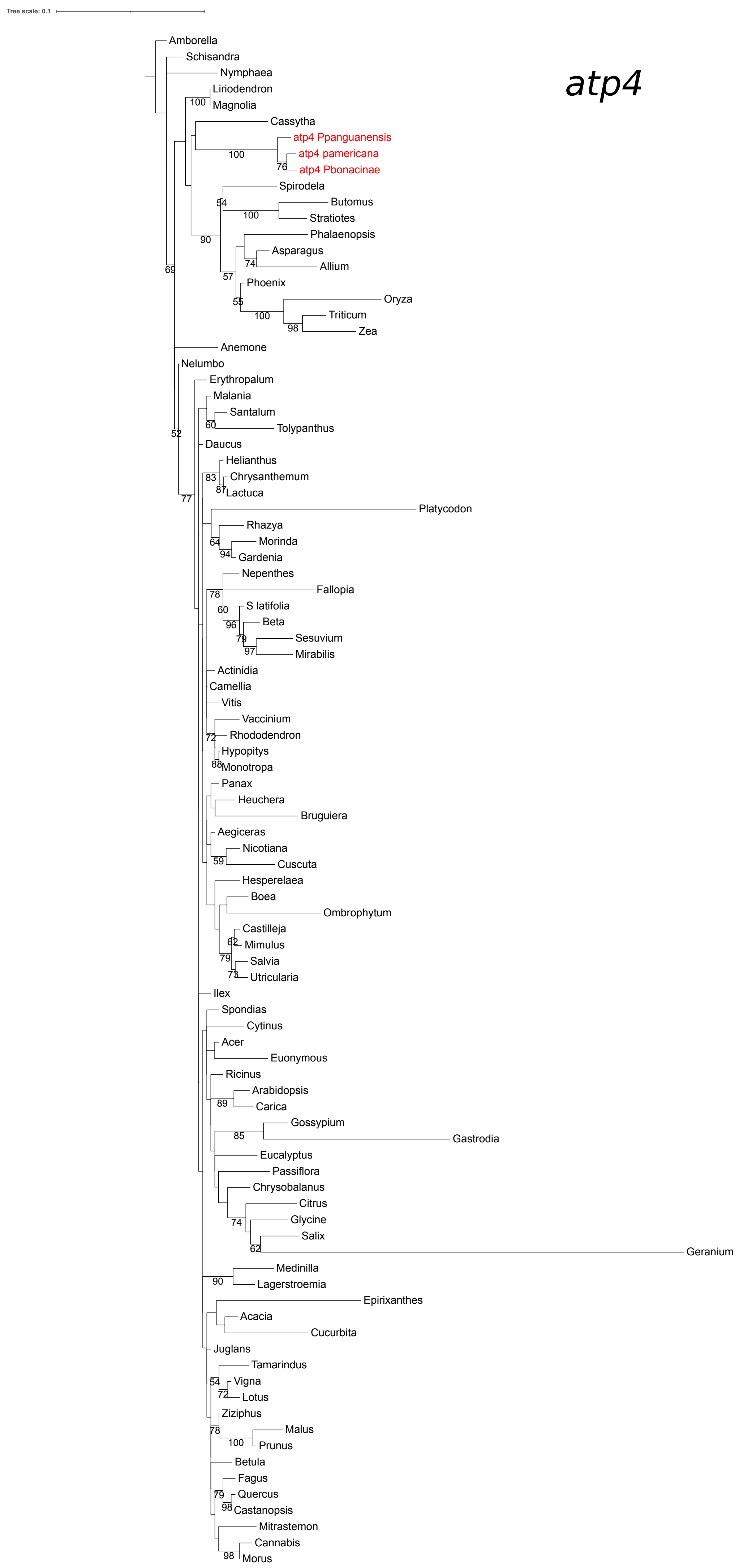

Tree scale: 0.1
atp4
Amborella
Schisandra
Nymphaea
Liriodendron
Magnolia
Cassytha
atp4 Ppanguanensis
atp4 pamericana
atp4 Pbonacinae
Spirodela
Butomus
Stratiotes
Phalaenopsis
Asparagus
Allium
Phoenix
Oryza
Triticum
Zea
Anemone
Nelumbo
Erythropalum
Malania
Santalum
Tolypanthus
Daucus
Helianthus
Chrysanthemum
Lactuca
Platycodon
Rhazya
Morinda
Gardenia
Nepenthes
Fallopia
S latifolia
Beta
Sesuvium
Mirabilis
Actinidia
Camellia
Vitis
Vaccinium
Rhododendron
Hypopitys
Monotropa
Panax
Heuchera
Bruguiera
Aegiceras
Nicotiana
Cuscuta
Hesperelaea
Boea
Ombrophytum
Castilleja
Mimulus
Salvia
Utricularia
Ilex
Spondias
Cytinus
Acer
Euonymous
Ricinus
Arabidopsis
Carica
Gossypium
Gastrodia
Eucalyptus
Passiflora
Chrysobalanus
Citrus
Glycine
Salix
Geranium
Medinilla
Lagerstroemia
Epirixanthes
Acacia
Cucurbita
Juglans
Tamarindus
Vigna
Lotus
Ziziphus
Malus
Prunus
Betula
Fagus
Quercus
Castanopsis
Mitrastemon
Cannabis
Morus

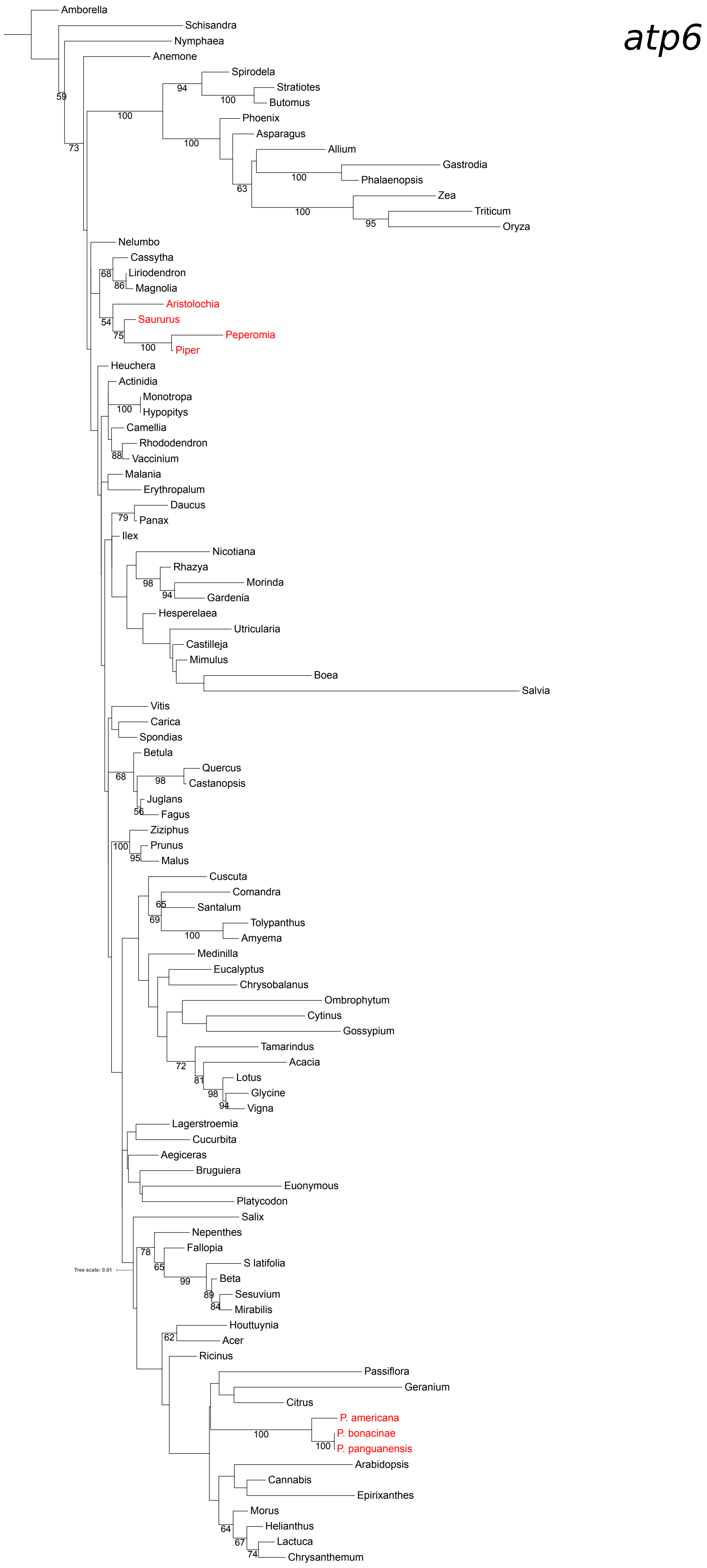
atp6
Amborella
Schisandra
Nymphaea
Anemone
Spirodela
Stratiotes
Butomus
Phoenix
Asparagus
Allium
Gastrodia
Phalaenopsis
Zea
Triticum
Oryza
Nelumbo
Cassytha
Liriodendron
Magnolia
Aristolochia
Saururus
Peperomia
Piper
Heuchera
Actinidia
Monotropa
Hypopitys
Camellia
Rhododendron
Vaccinium
Malania
Erythropalum
Daucus
Panax
Ilex
Nicotiana
Rhazya
Morinda
Gardenia
Hesperelaea
Utricularia
Castilleja
Mimulus
Boea
Salvia
Vitis
Carica
Spondias
Betula
Quercus
Castanopsis
Juglans
Fagus
Ziziphus
Prunus
Malus
Cuscuta
Comandra
Santalum
Tolypanthus
Amyema
Medinilla
Eucalyptus
Chrysobalanus
Ombrophytum
Cytinus
Gossypium
Tamarindus
Acacia
Lotus
Glycine
Vigna
Lagerstroemia
Cucurbita
Aegiceras
Bruguiera
Euonymous
Platycodon
Salix
Nepenthes
Fallopia
S latifolia
Beta
Sesuvium
Mirabilis
Houttuynia
Acer
Ricinus
Passiflora
Geranium
Citrus
P. americana
P. bonacinae
P. panguanensis
Arabidopsis
Cannabis
Epirixanthes
Morus
Helianthus
Lactuca
Chrysanthemum
Tree scale: 0.01

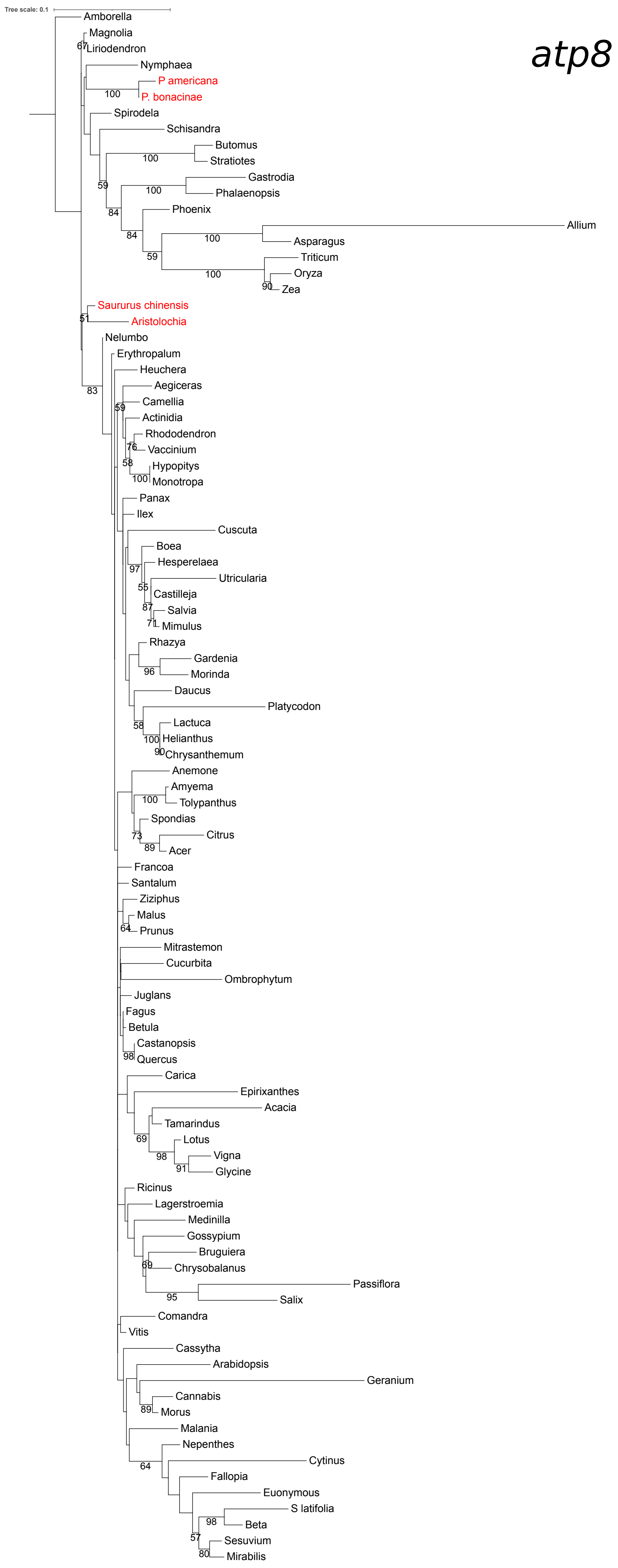
atp8
Tree scale: 0.1
Amborella
Magnolia
67
Liriodendron
Nymphaea
P americana
100
P. bonacinae
Spirodela
Schisandra
Butomus
100
Stratiotes
59
Gastrodia
100
Phalaenopsis
84
Phoenix
84
Allium
100
Asparagus
59
Triticum
100
Oryza
90
Zea
Saururus chinensis
51
Aristolochia
Nelumbo
Erythropalum
83
Heuchera
Aegiceras
Camellia
59
Actinidia
Rhododendron
76
Vaccinium
58
Hypopitys
100
Monotropa
Panax
Ilex
Cuscuta
Boea
97
Hesperelaea
55
Utricularia
Castilleja
87
Salvia
71
Mimulus
Rhazya
Gardenia
96
Morinda
Daucus
Platycodon
58
Lactuca
100
Helianthus
90
Chrysanthemum
Anemone
Amyema
100
Tolypanthus
Spondias
73
Citrus
89
Acer
Francoa
Santalum
Ziziphus
Malus
64
Prunus
Mitrastemon
Cucurbita
Ombrophytum
Juglans
Fagus
Betula
Castanopsis
98
Quercus
Carica
Epirixanthes
Acacia
Tamarindus
69
Lotus
98
Vigna
91
Glycine
Ricinus
Lagerstroemia
Medinilla
Gossypium
Bruguiera
69
Chrysobalanus
Passiflora
95
Salix
Comandra
Vitis
Cassytha
Arabidopsis
Geranium
Cannabis
89
Morus
Malania
Nepenthes
64
Cytinus
Fallopia
Euonymous
S latifolia
98
Beta
57
Sesuvium
80
Mirabilis

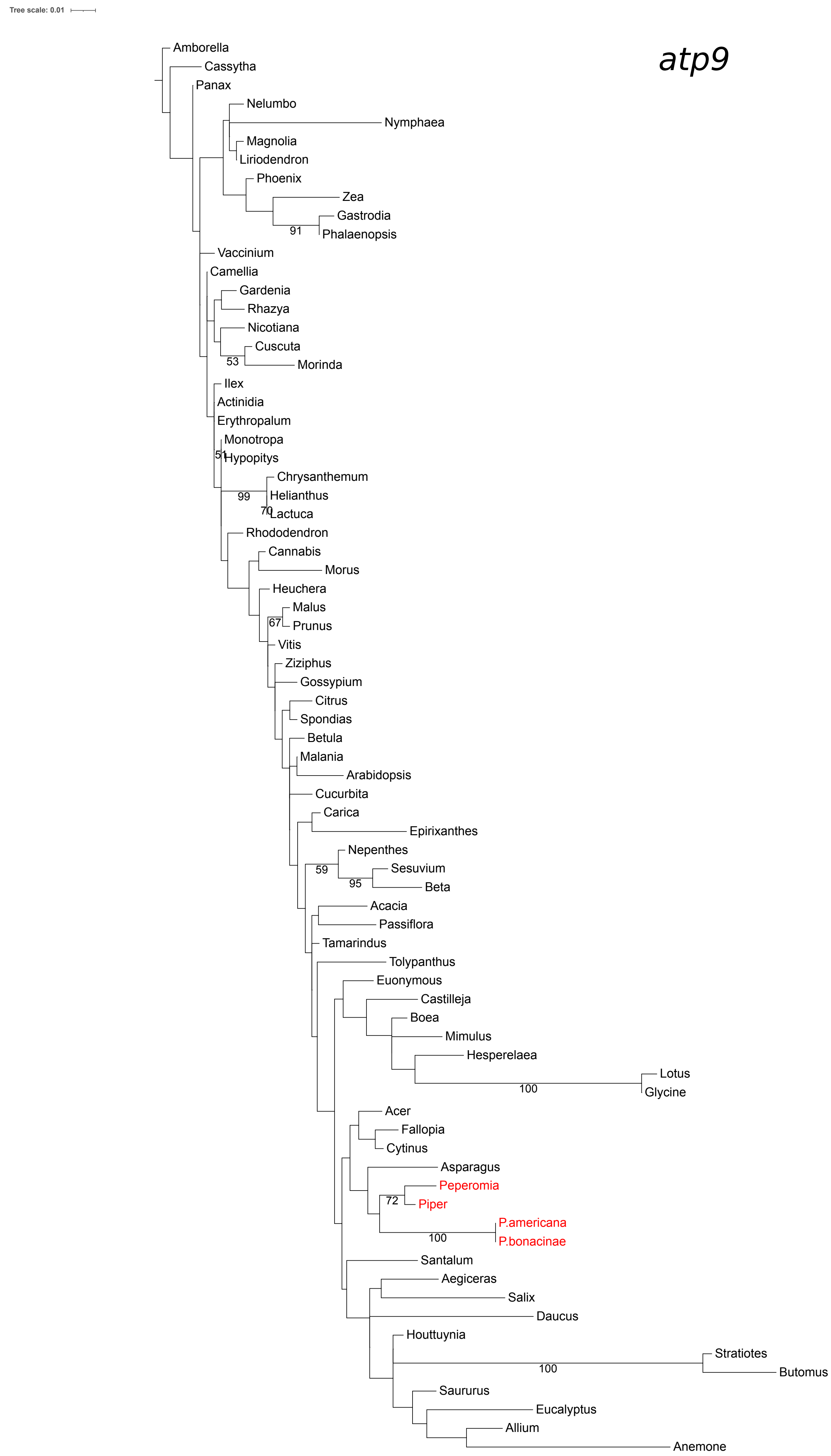

Tree scale: 0.01
atp9
Amborella
Cassytha
Panax
Nelumbo
Nymphaea
Magnolia
Liriodendron
Phoenix
Zea
Gastrodia
91
Phalaenopsis
Vaccinium
Camellia
Gardenia
Rhazya
Nicotiana
Cuscuta
53
Morinda
Ilex
Actinidia
Erythropalum
Monotropa
51
Hypopitys
Chrysanthemum
99
Helianthus
70
Lactuca
Rhododendron
Cannabis
Morus
Heuchera
Malus
67
Prunus
Vitis
Ziziphus
Gossypium
Citrus
Spondias
Betula
Malania
Arabidopsis
Cucurbita
Carica
Epirixanthes
Nepenthes
59
Sesuvium
95
Beta
Acacia
Passiflora
Tamarindus
Tolypanthus
Euonymous
Castilleja
Boea
Mimulus
Hesperelaea
Lotus
100
Glycine
Acer
Fallopia
Cytinus
Asparagus
Peperomia
72
Piper
P.americana
100
P.bonacinae
Santalum
Aegiceras
Salix
Daucus
Houttuynia
Stratiotes
100
Butomus
Saururus
Eucalyptus
Allium
Anemone

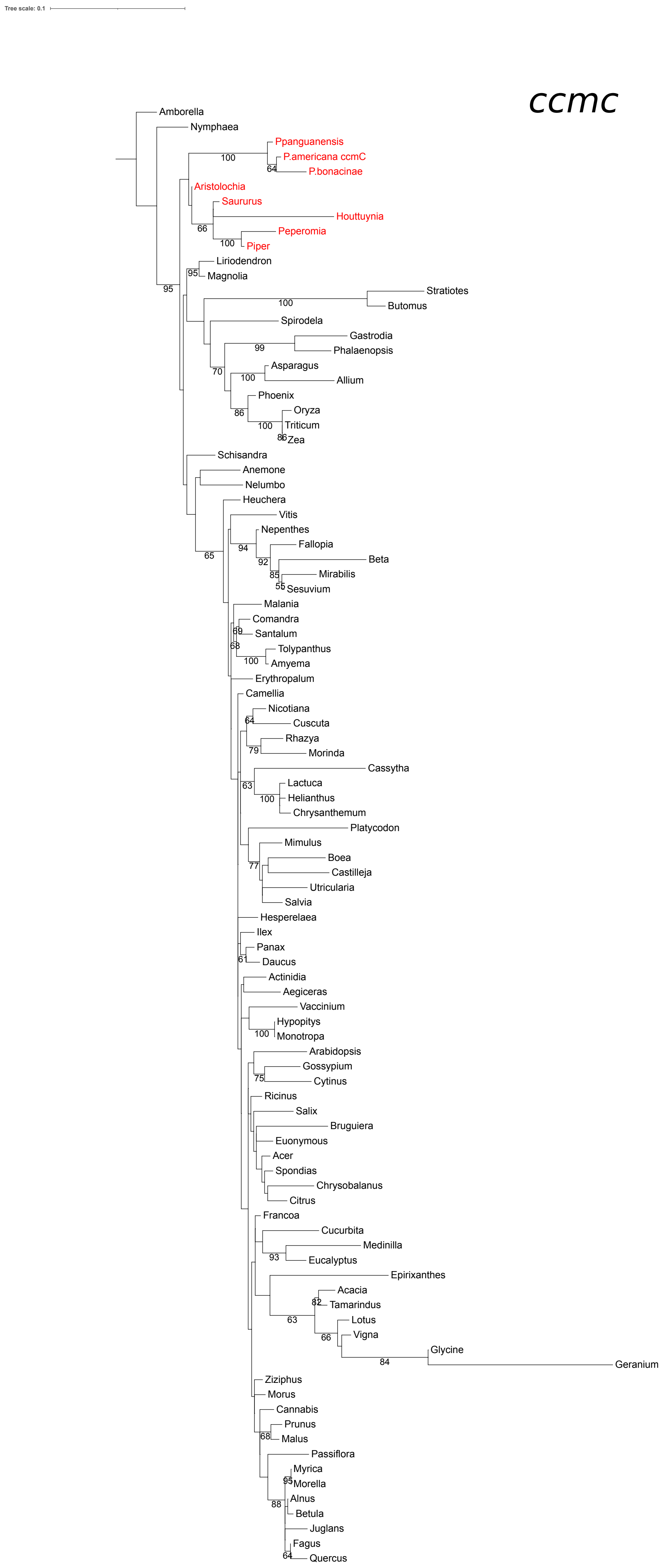
Tree scale: 0.1
ccmc
Amborella
Nymphaea
Ppanguanensis
P.americana ccmC
P.bonacinae
Aristolochia
Saururus
Houttuynia
Peperomia
Piper
Liriodendron
Magnolia
Stratiotes
Butomus
Spirodela
Gastrodia
Phalaenopsis
Asparagus
Allium
Phoenix
Oryza
Triticum
Zea
Schisandra
Anemone
Nelumbo
Heuchera
Vitis
Nepenthes
Fallopia
Beta
Mirabilis
Sesuvium
Malania
Comandra
Santalum
Tolypanthus
Amyema
Erythropalum
Camellia
Nicotiana
Cuscuta
Rhazya
Morinda
Cassytha
Lactuca
Helianthus
Chrysanthemum
Platycodon
Mimulus
Boea
Castilleja
Utricularia
Salvia
Hesperelaea
Ilex
Panax
Daucus
Actinidia
Aegiceras
Vaccinium
Hypopitys
Monotropa
Arabidopsis
Gossypium
Cytinus
Ricinus
Salix
Bruguiera
Euonymous
Acer
Spondias
Chrysobalanus
Citrus
Francoa
Cucurbita
Medinilla
Eucalyptus
Epirixanthes
Acacia
Tamarindus
Lotus
Vigna
Glycine
Geranium
Ziziphus
Morus
Cannabis
Prunus
Malus
Passiflora
Myrica
Morella
Alnus
Betula
Juglans
Fagus
Quercus

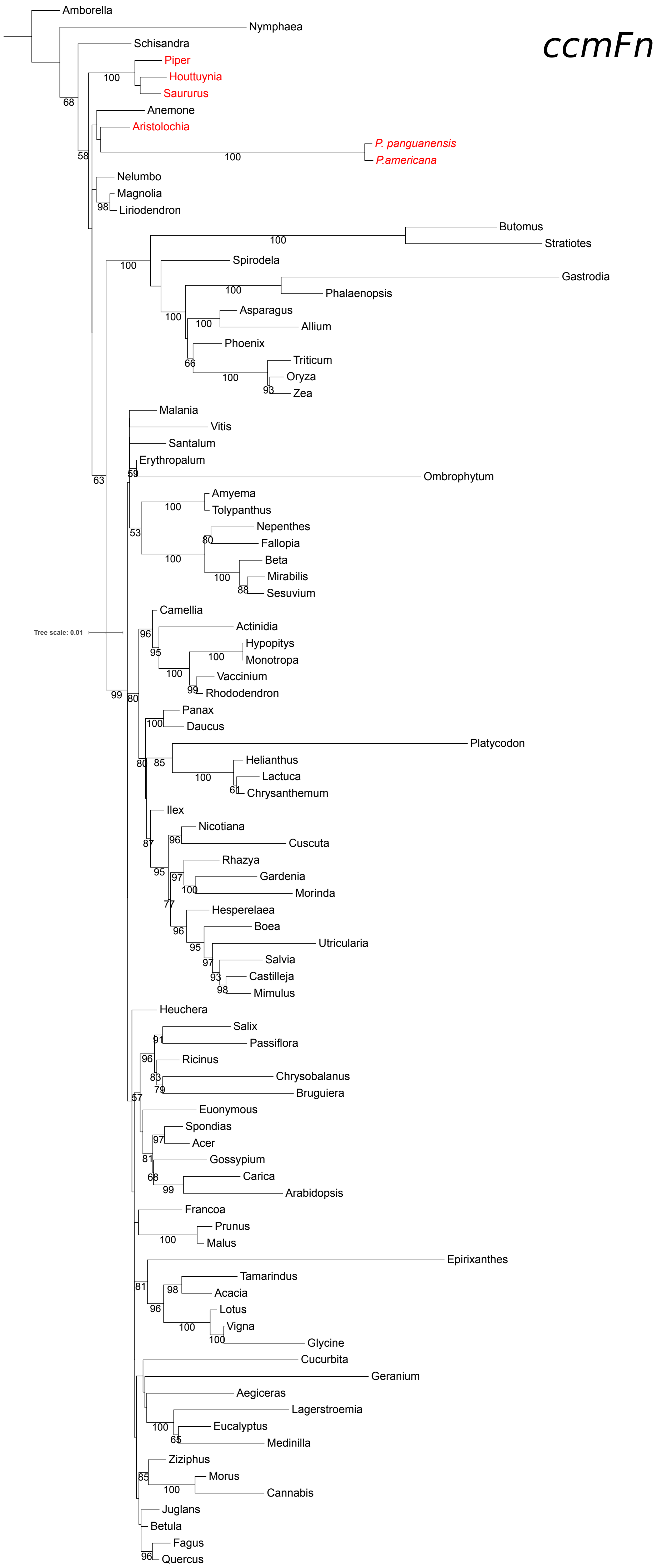
ccmFn
Amborella
Nymphaea
Schisandra
Piper
Houttuynia
Saururus
Anemone
Aristolochia
P. panguanensis
P.americana
Nelumbo
Magnolia
Liriodendron
Butomus
Stratiotes
Spirodela
Gastrodia
Phalaenopsis
Asparagus
Allium
Phoenix
Triticum
Oryza
Zea
Malania
Vitis
Santalum
Erythropalum
Ombrophytum
Amyema
Tolypanthus
Nepenthes
Fallopia
Beta
Mirabilis
Sesuvium
Camellia
Actinidia
Hypopitys
Monotropa
Vaccinium
Rhododendron
Panax
Daucus
Platycodon
Helianthus
Lactuca
Chrysanthemum
Ilex
Nicotiana
Cuscuta
Rhazya
Gardenia
Morinda
Hesperelaea
Boea
Utricularia
Salvia
Castilleja
Mimulus
Heuchera
Salix
Passiflora
Ricinus
Chrysobalanus
Bruguiera
Euonymous
Spondias
Acer
Gossypium
Carica
Arabidopsis
Francoa
Prunus
Malus
Epirixanthes
Tamarindus
Acacia
Lotus
Vigna
Glycine
Cucurbita
Geranium
Aegiceras
Lagerstroemia
Eucalyptus
Medinilla
Ziziphus
Morus
Cannabis
Juglans
Betula
Fagus
Quercus
Tree scale: 0.01
100
68
58
100
98
100
100
100
100
100
66
100
93
59
53
100
80
100
100
88
63
96
95
100
100
99
99
80
100
80
85
100
61
87
96
95
97
100
77
96
95
97
93
98
91
96
83
79
57
97
81
68
99
100
81
98
96
100
100
100
65
85
100
96

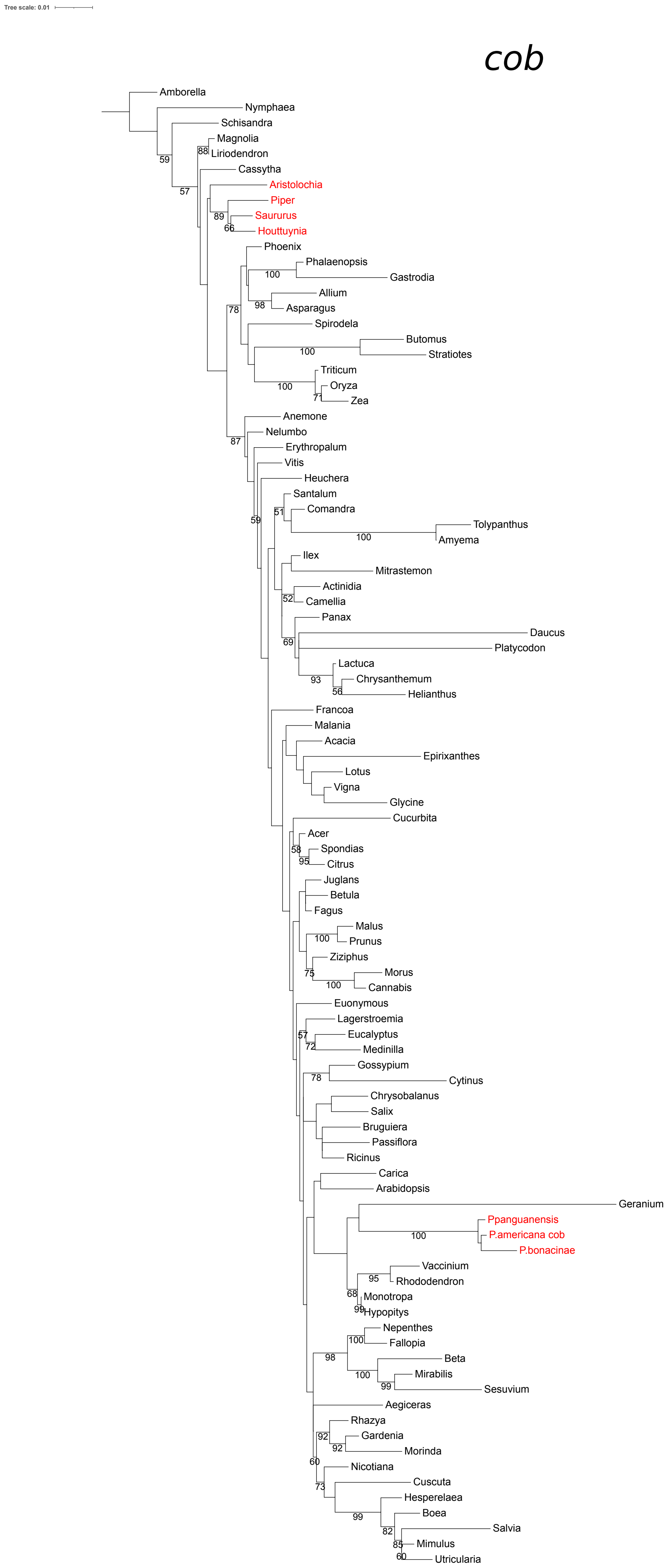

Tree scale: 0.01
cob
Amborella
Nymphaea
Schisandra
Magnolia
Liriodendron
Cassytha
Aristolochia
Piper
Saururus
Houttuynia
Phoenix
Phalaenopsis
Gastrodia
Allium
Asparagus
Spirodela
Butomus
Stratiotes
Triticum
Oryza
Zea
Anemone
Nelumbo
Erythropalum
Vitis
Heuchera
Santalum
Comandra
Tolypanthus
Amyema
Ilex
Mitrastemon
Actinidia
Camellia
Panax
Daucus
Platycodon
Lactuca
Chrysanthemum
Helianthus
Francoa
Malania
Acacia
Epirixanthes
Lotus
Vigna
Glycine
Cucurbita
Acer
Spondias
Citrus
Juglans
Betula
Fagus
Malus
Prunus
Ziziphus
Morus
Cannabis
Euonymous
Lagerstroemia
Eucalyptus
Medinilla
Gossypium
Cytinus
Chrysobalanus
Salix
Bruguiera
Passiflora
Ricinus
Carica
Arabidopsis
Geranium
Ppanguanensis
P.americana cob
P.bonacinae
Vaccinium
Rhododendron
Monotropa
Hypopitys
Nepenthes
Fallopia
Beta
Mirabilis
Sesuvium
Aegiceras
Rhazya
Gardenia
Morinda
Nicotiana
Cuscuta
Hesperelaea
Boea
Salvia
Mimulus
Utricularia

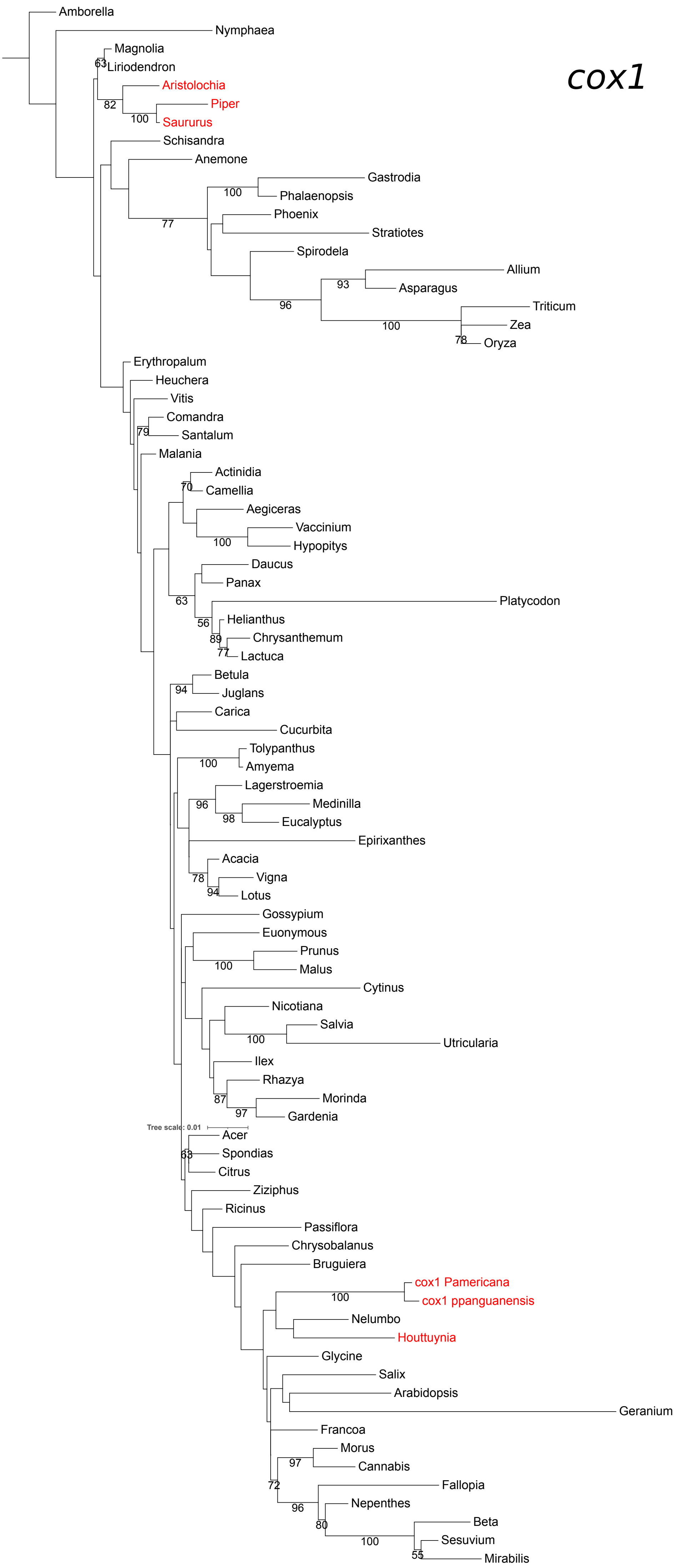
cox1
Amborella
Nymphaea
Magnolia
Liriodendron
Aristolochia
Piper
Saururus
Schisandra
Anemone
Gastrodia
Phalaenopsis
Phoenix
Stratiotes
Spirodela
Allium
Asparagus
Triticum
Zea
Oryza
Erythropalum
Heuchera
Vitis
Comandra
Santalum
Malania
Actinidia
Camellia
Aegiceras
Vaccinium
Hypopitys
Daucus
Panax
Platycodon
Helianthus
Chrysanthemum
Lactuca
Betula
Juglans
Carica
Cucurbita
Tolypanthus
Amyema
Lagerstroemia
Medinilla
Eucalyptus
Epirixanthes
Acacia
Vigna
Lotus
Gossypium
Euonymous
Prunus
Malus
Cytinus
Nicotiana
Salvia
Utricularia
Ilex
Rhazya
Morinda
Gardenia
Tree scale: 0.01
Acer
Spondias
Citrus
Ziziphus
Ricinus
Passiflora
Chrysobalanus
Bruguiera
cox1 Pamericana
cox1 ppanguanensis
Nelumbo
Houttuynia
Glycine
Salix
Arabidopsis
Geranium
Francoa
Morus
Cannabis
Fallopia
Nepenthes
Beta
Sesuvium
Mirabilis

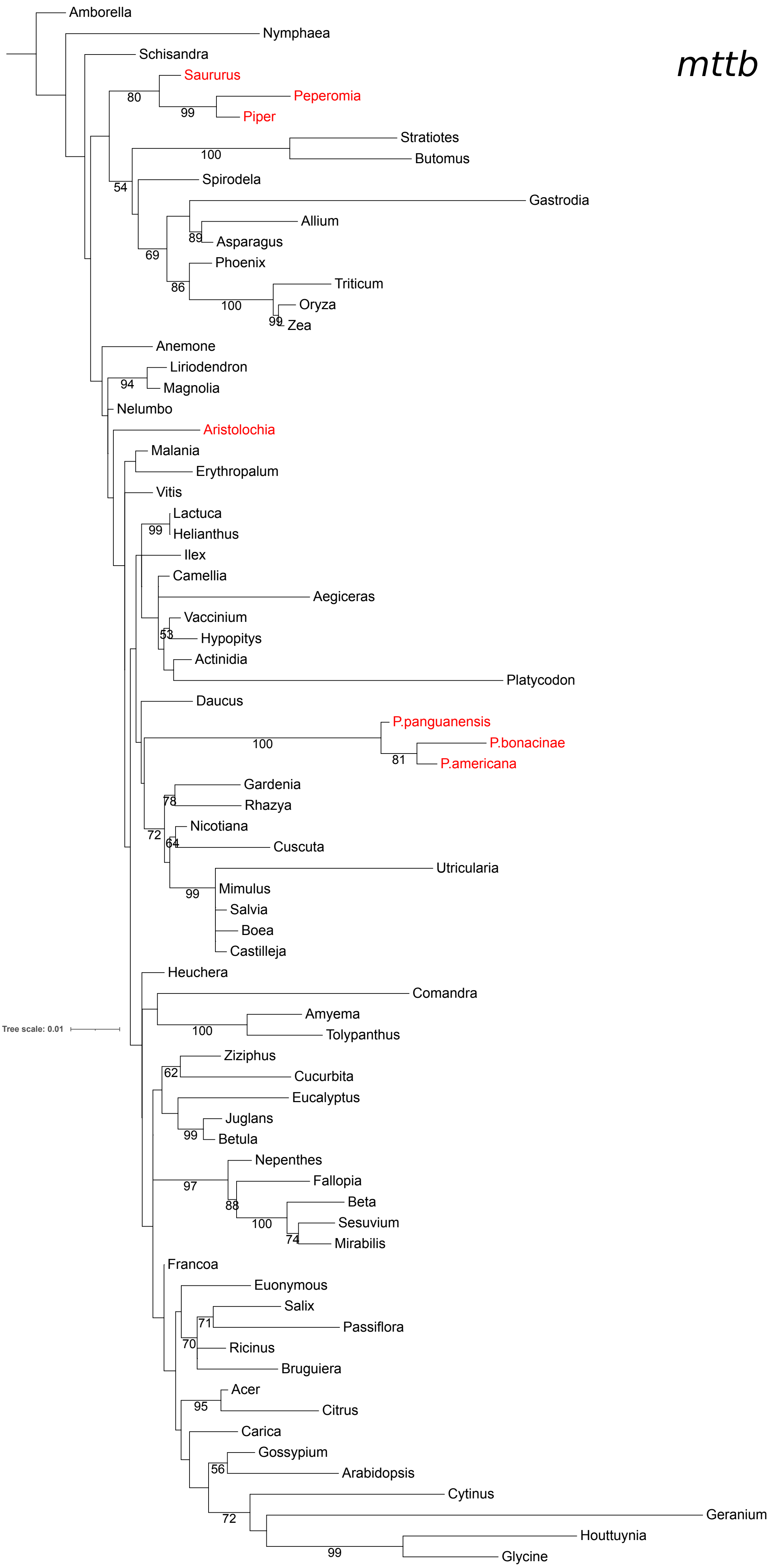
mttb
Amborella
Nymphaea
Schisandra
Saururus
Peperomia
Piper
Stratiotes
Butomus
Spirodela
Gastrodia
Allium
Asparagus
Phoenix
Triticum
Oryza
Zea
Anemone
Liriodendron
Magnolia
Nelumbo
Aristolochia
Malania
Erythropalum
Vitis
Lactuca
Helianthus
Ilex
Camellia
Aegiceras
Vaccinium
Hypopitys
Actinidia
Platycodon
Daucus
P.panguanensis
P.bonacinae
P.americana
Gardenia
Rhazya
Nicotiana
Cuscuta
Utricularia
Mimulus
Salvia
Boea
Castilleja
Heuchera
Comandra
Amyema
Tolypanthus
Ziziphus
Cucurbita
Eucalyptus
Juglans
Betula
Nepenthes
Fallopia
Beta
Sesuvium
Mirabilis
Francoa
Euonymous
Salix
Passiflora
Ricinus
Bruguiera
Acer
Citrus
Carica
Gossypium
Arabidopsis
Cytinus
Geranium
Houttuynia
Glycine
Tree scale: 0.01

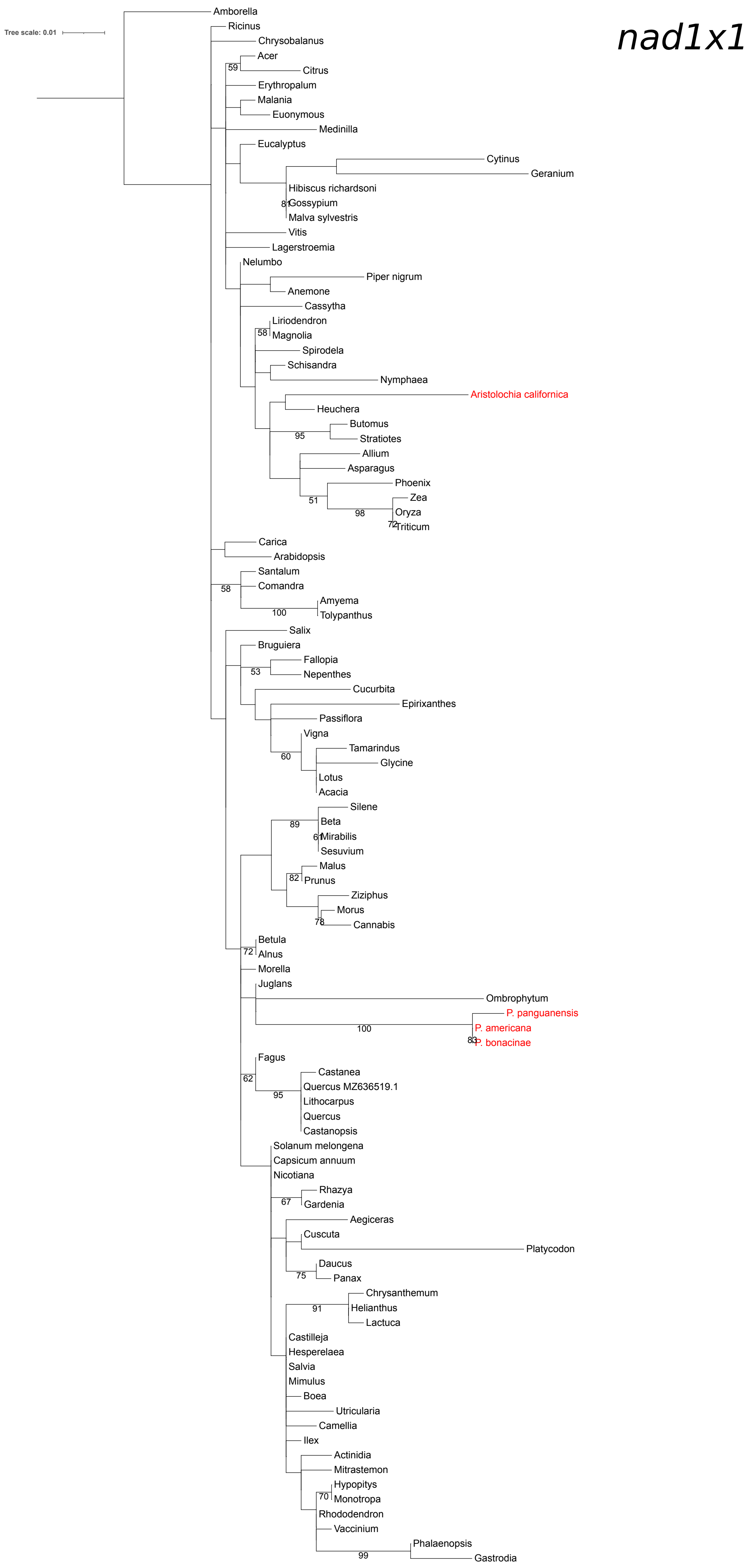
nad1x1
Tree scale: 0.01
Amborella
Ricinus
Chrysobalanus
Acer
Citrus
Erythropalum
Malania
Euonymous
Medinilla
Eucalyptus
Cytinus
Geranium
Hibiscus richardsoni
Gossypium
Malva sylvestris
Vitis
Lagerstroemia
Nelumbo
Piper nigrum
Anemone
Cassytha
Liriodendron
Magnolia
Spirodela
Schisandra
Nymphaea
Aristolochia californica
Heuchera
Butomus
Stratiotes
Allium
Asparagus
Phoenix
Zea
Oryza
Triticum
Carica
Arabidopsis
Santalum
Comandra
Amyema
Tolypanthus
Salix
Bruguiera
Fallopia
Nepenthes
Cucurbita
Epirixanthes
Passiflora
Vigna
Tamarindus
Glycine
Lotus
Acacia
Silene
Beta
Mirabilis
Sesuvium
Malus
Prunus
Ziziphus
Morus
Cannabis
Betula
Alnus
Morella
Juglans
Ombrophytum
P. panguanensis
P. americana
P. bonacinae
Fagus
Castanea
Quercus MZ636519.1
Lithocarpus
Quercus
Castanopsis
Solanum melongena
Capsicum annuum
Nicotiana
Rhazya
Gardenia
Aegiceras
Cuscuta
Platycodon
Daucus
Panax
Chrysanthemum
Helianthus
Lactuca
Castilleja
Hesperelaea
Salvia
Mimulus
Boea
Utricularia
Camellia
Ilex
Actinidia
Mitrastemon
Hypopitys
Monotropa
Rhododendron
Vaccinium
Phalaenopsis
Gastrodia

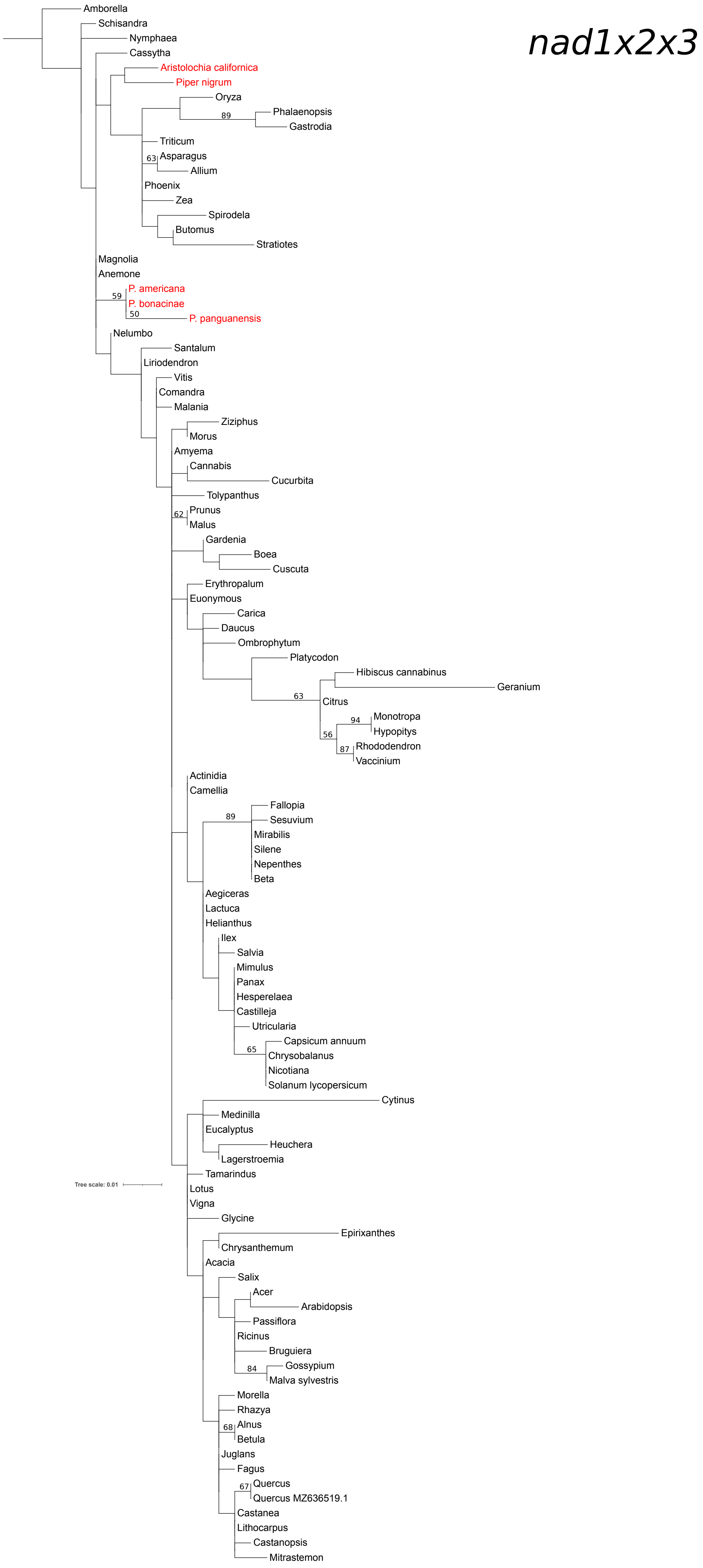
nad1x2x3
Amborella
Schisandra
Nymphaea
Cassytha
Aristolochia californica
Piper nigrum
Oryza
89
Phalaenopsis
Gastrodia
Triticum
63
Asparagus
Allium
Phoenix
Zea
Spirodela
Butomus
Stratiotes
Magnolia
Anemone
59
P. americana
P. bonacinae
50
P. panguanensis
Nelumbo
Santalum
Liriodendron
Vitis
Comandra
Malania
Ziziphus
Morus
Amyema
Cannabis
Cucurbita
Tolypanthus
62
Prunus
Malus
Gardenia
Boea
Cuscuta
Erythropalum
Euonymous
Carica
Daucus
Ombrophytum
Platycodon
Hibiscus cannabinus
Geranium
63
Citrus
94
Monotropa
Hypopitys
56
87
Rhododendron
Vaccinium
Actinidia
Camellia
89
Fallopia
Sesuvium
Mirabilis
Silene
Nepenthes
Beta
Aegiceras
Lactuca
Helianthus
Ilex
Salvia
Mimulus
Panax
Hesperelaea
Castilleja
Utricularia
65
Capsicum annuum
Chrysobalanus
Nicotiana
Solanum lycopersicum
Cytinus
Medinilla
Eucalyptus
Heuchera
Lagerstroemia
Tamarindus
Tree scale: 0.01
Lotus
Vigna
Glycine
Epirixanthes
Chrysanthemum
Acacia
Salix
Acer
Arabidopsis
Passiflora
Ricinus
Bruguiera
84
Gossypium
Malva sylvestris
Morella
Rhazya
68
Alnus
Betula
Juglans
Fagus
67
Quercus
Quercus MZ636519.1
Castanea
Lithocarpus
Castanopsis
Mitrastemon

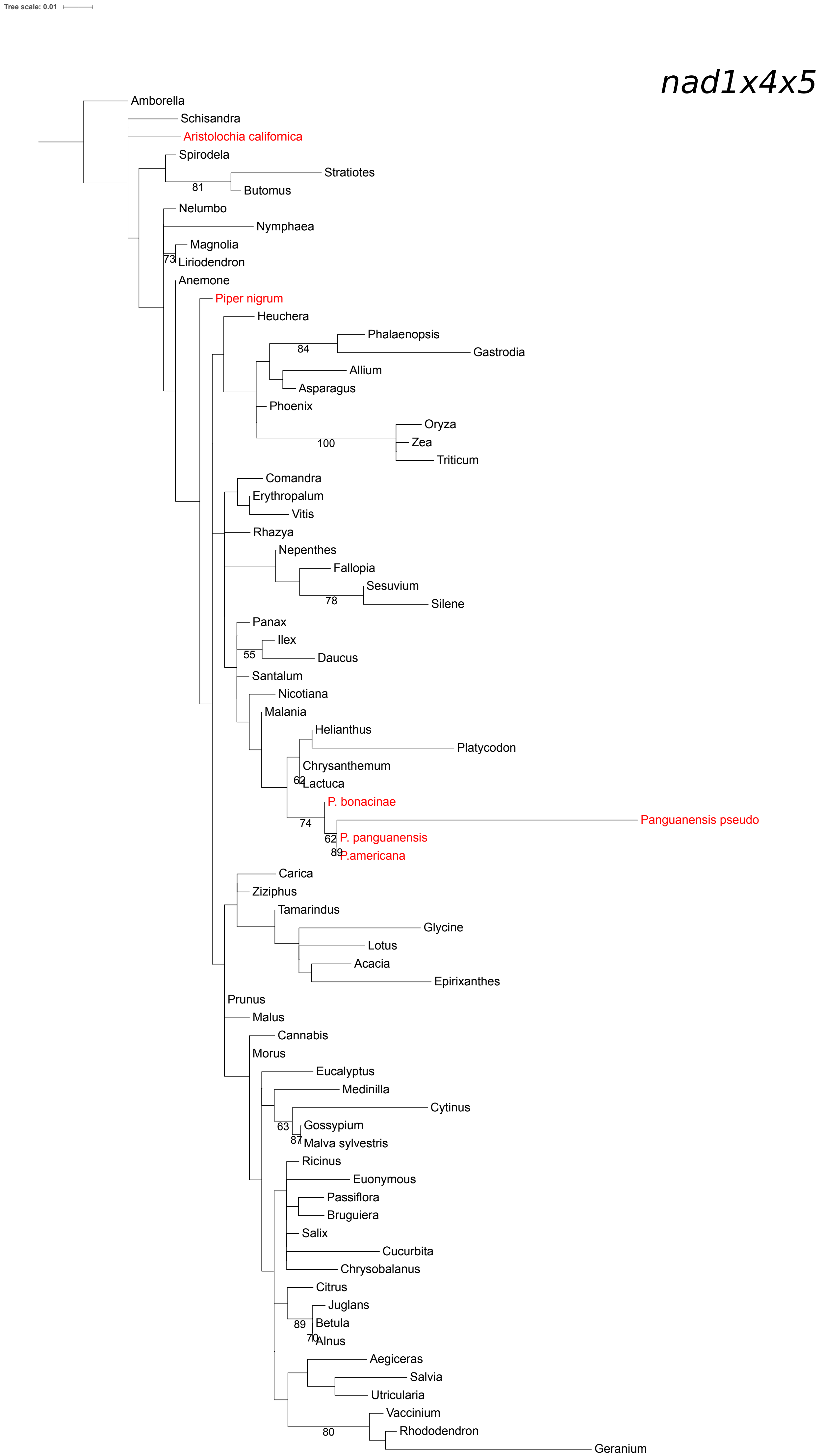

Tree scale: 0.01
nad1x4x5
Amborella
Schisandra
Aristolochia californica
Spirodela
Stratiotes
81
Butomus
Nelumbo
Nymphaea
Magnolia
73
Liriodendron
Anemone
Piper nigrum
Heuchera
Phalaenopsis
84
Gastrodia
Allium
Asparagus
Phoenix
Oryza
100
Zea
Triticum
Comandra
Erythropalum
Vitis
Rhazya
Nepenthes
Fallopia
Sesuvium
78
Silene
Panax
Ilex
55
Daucus
Santalum
Nicotiana
Malania
Helianthus
Platycodon
Chrysanthemum
62
Lactuca
P. bonacinae
Panguanensis pseudo
74
62
P. panguanensis
89
P.americana
Carica
Ziziphus
Tamarindus
Glycine
Lotus
Acacia
Epirixanthes
Prunus
Malus
Cannabis
Morus
Eucalyptus
Medinilla
Cytinus
63
Gossypium
87
Malva sylvestris
Ricinus
Euonymous
Passiflora
Bruguiera
Salix
Cucurbita
Chrysobalanus
Citrus
Juglans
89
Betula
70
Alnus
Aegiceras
Salvia
Utricularia
Vaccinium
80
Rhododendron
Geranium

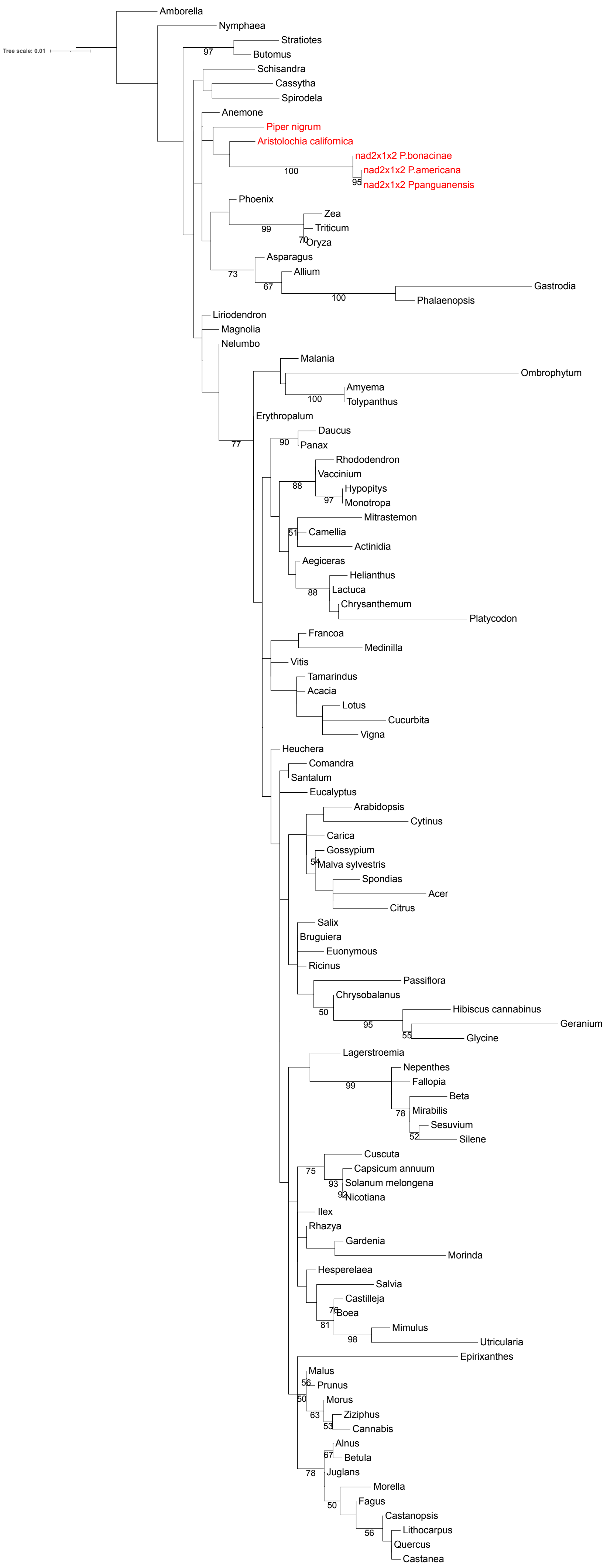
Tree scale: 0.01
Amborella
Nymphaea
Stratiotes
Butomus
Schisandra
Cassytha
Spirodela
Anemone
Piper nigrum
Aristolochia californica
nad2x1x2 P.bonacinae
nad2x1x2 P.americana
nad2x1x2 Ppanguanensis
Phoenix
Zea
Triticum
Oryza
Asparagus
Allium
Gastrodia
Phalaenopsis
Liriodendron
Magnolia
Nelumbo
Malania
Ombrophytum
Amyema
Tolypanthus
Erythropalum
Daucus
Panax
Rhododendron
Vaccinium
Hypopitys
Monotropa
Mitrastemon
Camellia
Actinidia
Aegiceras
Helianthus
Lactuca
Chrysanthemum
Platycodon
Francoa
Medinilla
Vitis
Tamarindus
Acacia
Lotus
Cucurbita
Vigna
Heuchera
Comandra
Santalum
Eucalyptus
Arabidopsis
Cytinus
Carica
Gossypium
Malva sylvestris
Spondias
Acer
Citrus
Salix
Bruguiera
Euonymous
Ricinus
Passiflora
Chrysobalanus
Hibiscus cannabinus
Geranium
Glycine
Lagerstroemia
Nepenthes
Fallopia
Beta
Mirabilis
Sesuvium
Silene
Cuscuta
Capsicum annuum
Solanum melongena
Nicotiana
Ilex
Rhazya
Gardenia
Morinda
Hesperelaea
Salvia
Castilleja
Boea
Mimulus
Utricularia
Epirixanthes
Malus
Prunus
Morus
Ziziphus
Cannabis
Alnus
Betula
Juglans
Morella
Fagus
Castanopsis
Lithocarpus
Quercus
Castanea

nad2x1x2

nad2x3x4x5

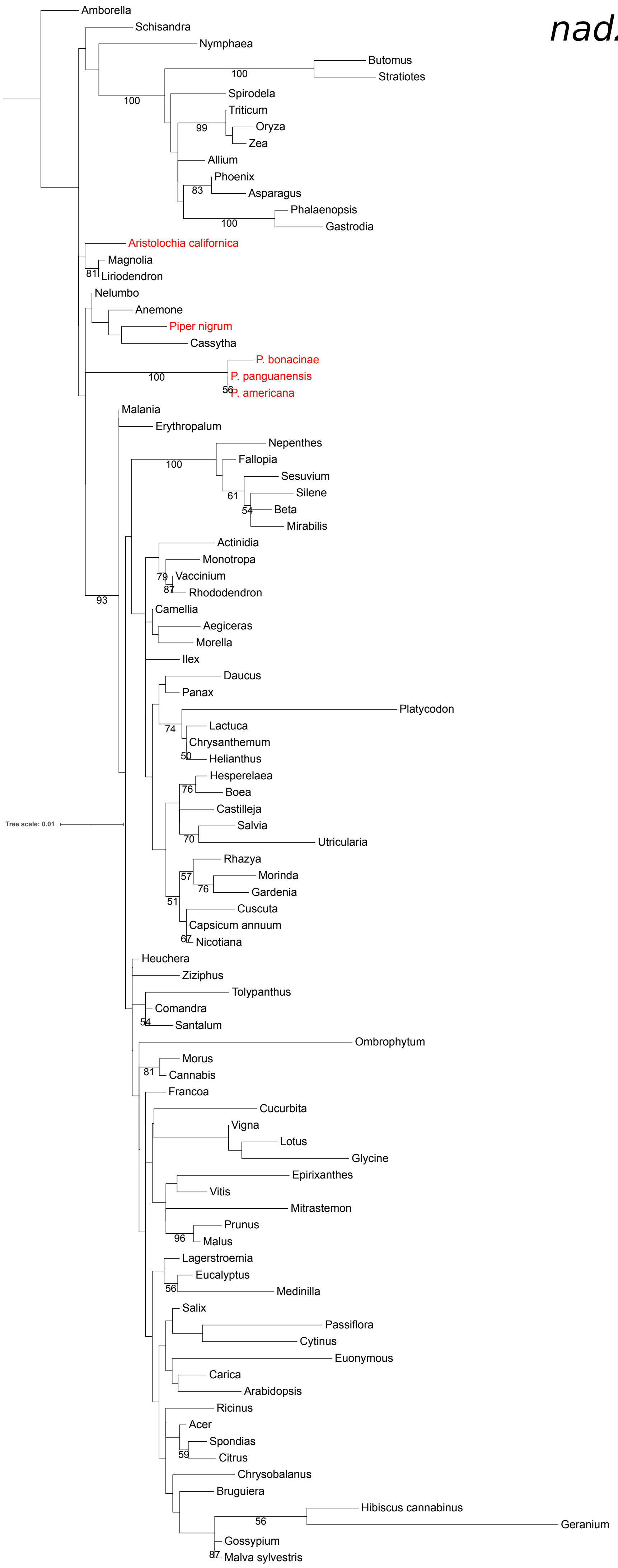
Amborella
Schisandra
Nymphaea
Butomus
Stratiotes
Spirodela
Triticum
Oryza
Zea
Allium
Phoenix
Asparagus
Phalaenopsis
Gastrodia
Aristolochia californica
Magnolia
Liriodendron
Nelumbo
Anemone
Piper nigrum
Cassytha
P. bonacinae
P. panguanensis
P. americana
Malania
Erythropalum
Nepenthes
Fallopia
Sesuvium
Silene
Beta
Mirabilis
Actinidia
Monotropa
Vaccinium
Rhododendron
Camellia
Aegiceras
Morella
Ilex
Daucus
Panax
Platycodon
Lactuca
Chrysanthemum
Helianthus
Hesperelaea
Boea
Castilleja
Salvia
Utricularia
Rhazya
Morinda
Gardenia
Cuscuta
Capsicum annuum
Nicotiana
Heuchera
Ziziphus
Tolypanthus
Comandra
Santalum
Ombrophytum
Morus
Cannabis
Francoa
Cucurbita
Vigna
Lotus
Glycine
Epirixanthes
Vitis
Mitrastemon
Prunus
Malus
Lagerstroemia
Eucalyptus
Medinilla
Salix
Passiflora
Cytinus
Euonymous
Carica
Arabidopsis
Ricinus
Acer
Spondias
Citrus
Chrysobalanus
Bruguiera
Hibiscus cannabinus
Geranium
Gossypium
Malva sylvestris
Tree scale: 0.01

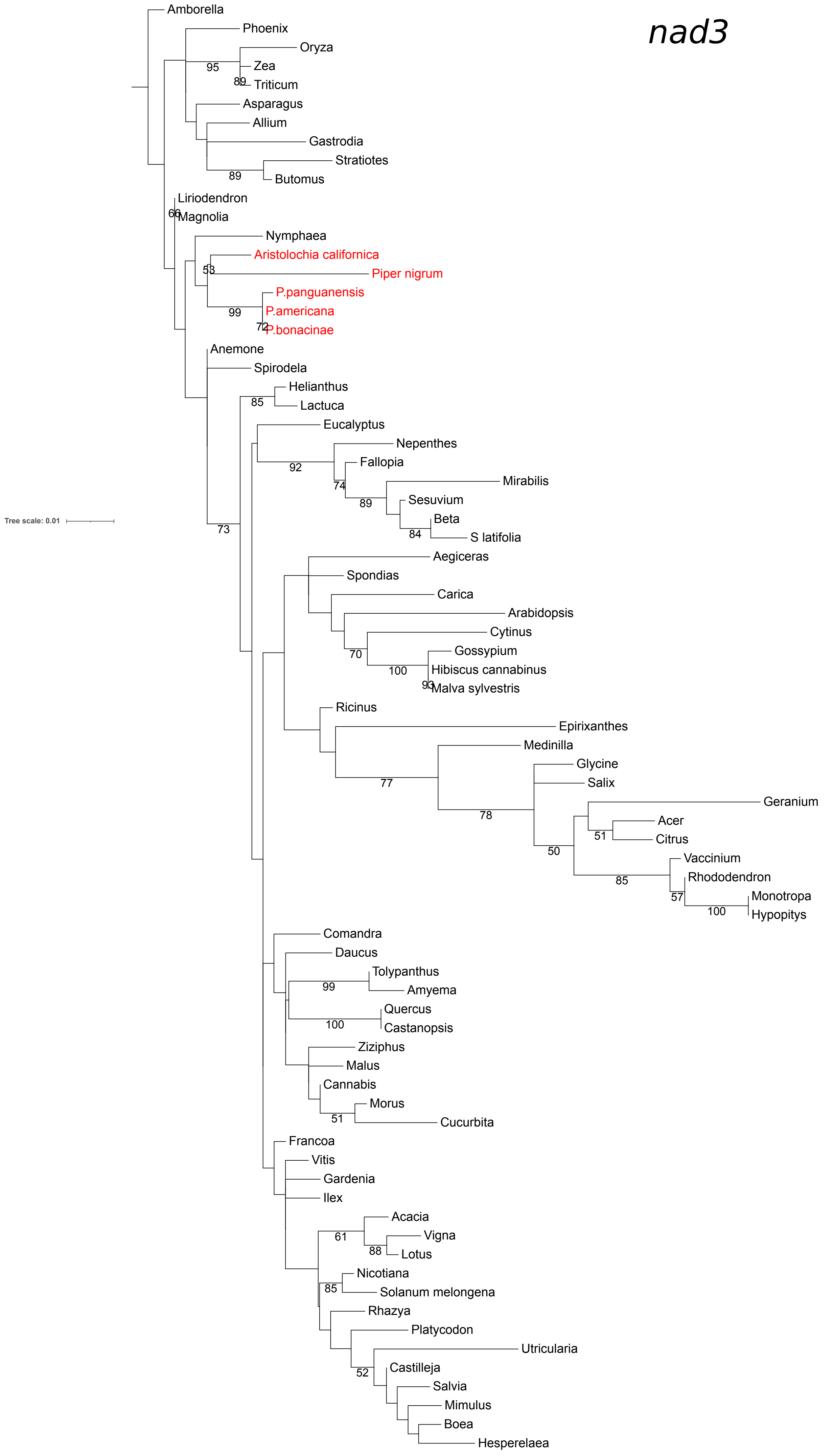
nad3
Tree scale: 0.01
Amborella
Phoenix
Oryza
95
Zea
89
Triticum
Asparagus
Allium
Gastrodia
Stratiotes
89
Butomus
Liriodendron
66
Magnolia
Nymphaea
Aristolochia californica
53
Piper nigrum
P.panguanensis
99
P.americana
72
P.bonacinae
Anemone
Spirodela
Helianthus
85
Lactuca
Eucalyptus
Nepenthes
92
Fallopia
74
Mirabilis
89
Sesuvium
Beta
84
S latifolia
73
Aegiceras
Spondias
Carica
Arabidopsis
Cytinus
70
Gossypium
100
Hibiscus cannabinus
93
Malva sylvestris
Ricinus
Epirixanthes
Medinilla
77
Glycine
Salix
78
Geranium
Acer
51
Citrus
50
Vaccinium
85
Rhododendron
57
Monotropa
100
Hypopitys
Comandra
Daucus
Tolypanthus
99
Amyema
Quercus
100
Castanopsis
Ziziphus
Malus
Cannabis
Morus
51
Cucurbita
Francoa
Vitis
Gardenia
Ilex
Acacia
61
Vigna
88
Lotus
Nicotiana
85
Solanum melongena
Rhazya
Platycodon
Utricularia
52
Castilleja
Salvia
Mimulus
Boea
Hesperelaea

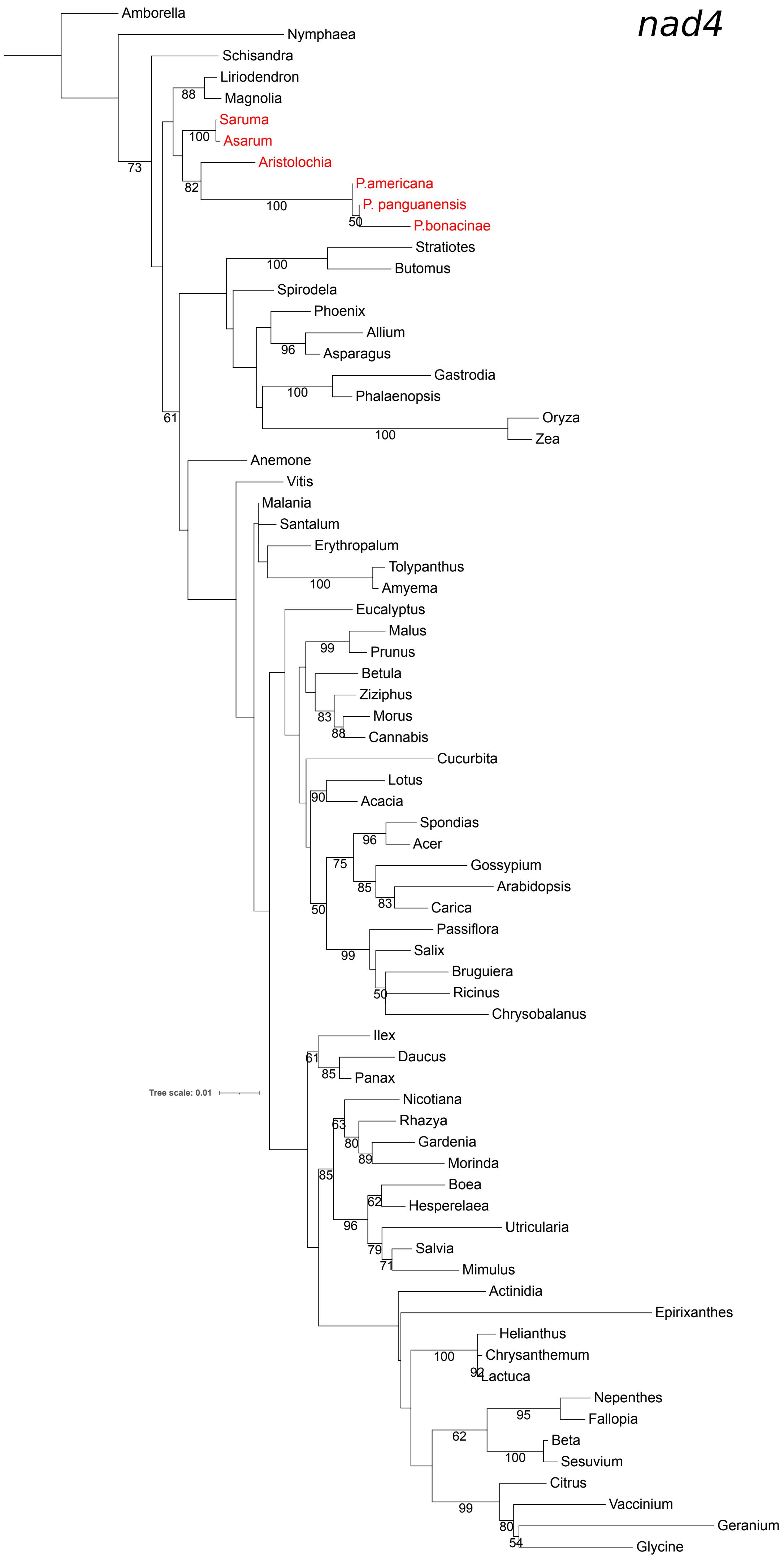
nad4
Amborella
Nymphaea
Schisandra
Liriodendron
Magnolia
Saruma
Asarum
Aristolochia
P.americana
P. panguanensis
P.bonacinae
Stratiotes
Butomus
Spirodela
Phoenix
Allium
Asparagus
Gastrodia
Phalaenopsis
Oryza
Zea
Anemone
Vitis
Malania
Santalum
Erythropalum
Tolypanthus
Amyema
Eucalyptus
Malus
Prunus
Betula
Ziziphus
Morus
Cannabis
Cucurbita
Lotus
Acacia
Spondias
Acer
Gossypium
Arabidopsis
Carica
Passiflora
Salix
Bruguiera
Ricinus
Chrysobalanus
Ilex
Daucus
Panax
Nicotiana
Rhazya
Gardenia
Morinda
Boea
Hesperelaea
Utricularia
Salvia
Mimulus
Actinidia
Epirixanthes
Helianthus
Chrysanthemum
Lactuca
Nepenthes
Fallopia
Beta
Sesuvium
Citrus
Vaccinium
Geranium
Glycine
Tree scale: 0.01

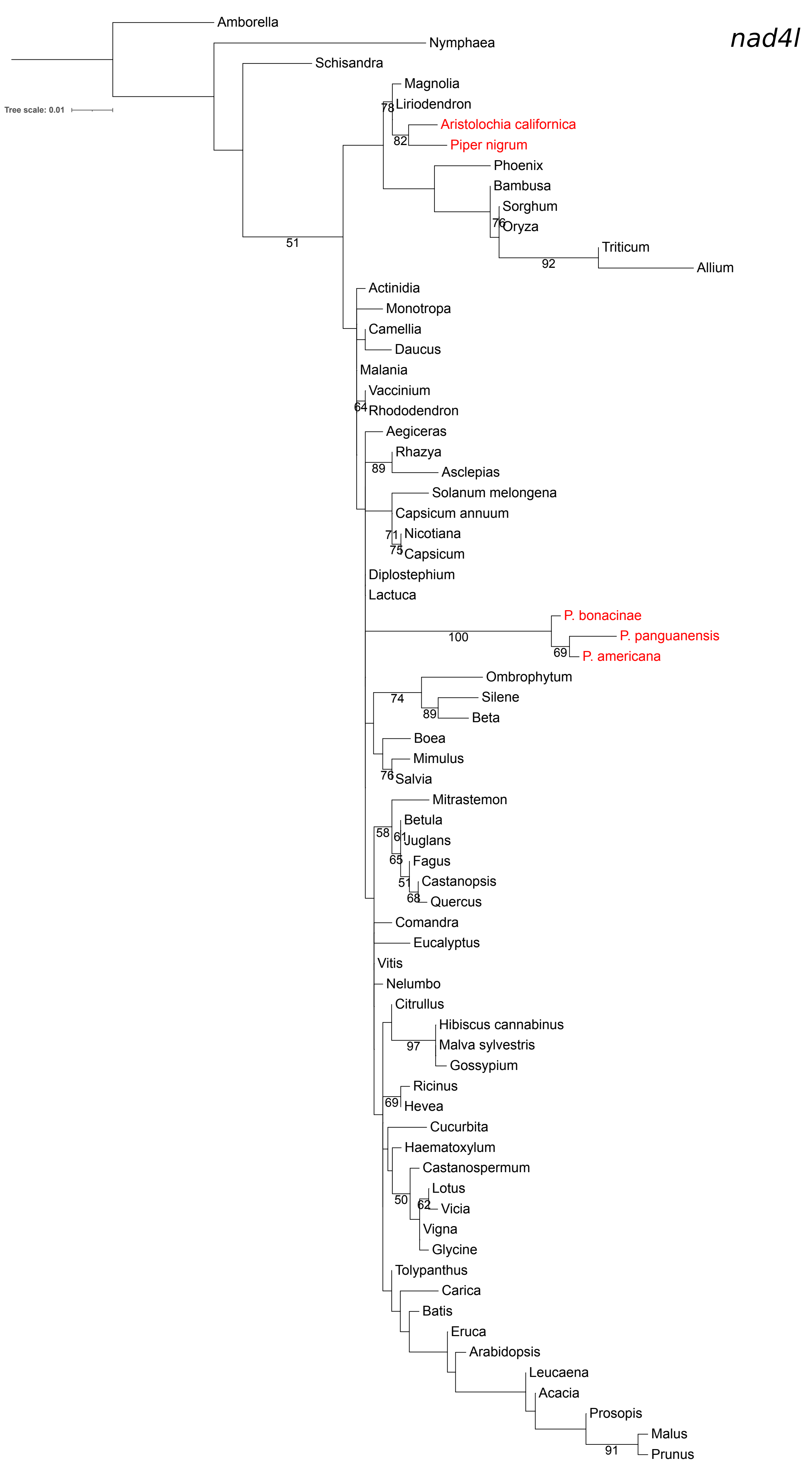
nad4l
Tree scale: 0.01
Amborella
Nymphaea
Schisandra
Magnolia
Liriodendron
78
Aristolochia californica
Piper nigrum
82
Phoenix
Bambusa
Sorghum
76
Oryza
Triticum
92
Allium
51
Actinidia
Monotropa
Camellia
Daucus
Malania
Vaccinium
64
Rhododendron
Aegiceras
Rhazya
89
Asclepias
Solanum melongena
Capsicum annuum
71
Nicotiana
75
Capsicum
Diplostephium
Lactuca
P. bonacinae
100
P. panguanensis
69
P. americana
Ombrophytum
74
Silene
89
Beta
Boea
Mimulus
76
Salvia
Mitrastemon
Betula
58
61
Juglans
65
Fagus
51
Castanopsis
68
Quercus
Comandra
Eucalyptus
Vitis
Nelumbo
Citrullus
Hibiscus cannabinus
97
Malva sylvestris
Gossypium
Ricinus
69
Hevea
Cucurbita
Haematoxylum
Castanospermum
Lotus
50
62
Vicia
Vigna
Glycine
Tolypanthus
Carica
Batis
Eruca
Arabidopsis
Leucaena
Acacia
Prosopis
Malus
91
Prunus

nad5x1x2

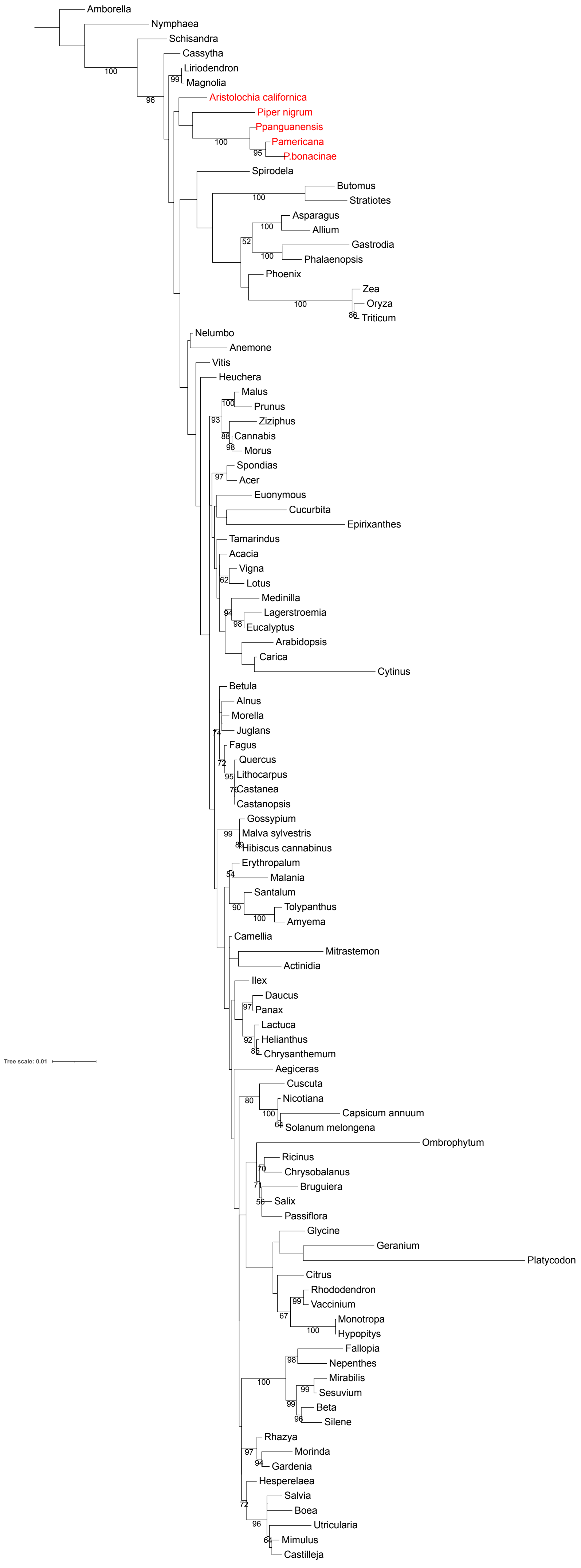
Amborella
Nymphaea
Schisandra
Cassytha
Liriodendron
Magnolia
Aristolochia californica
Piper nigrum
Ppanguanensis
Pamericana
P.bonacinae
Spirodela
Butomus
Stratiotes
Asparagus
Allium
Gastrodia
Phalaenopsis
Phoenix
Zea
Oryza
Triticum
Nelumbo
Anemone
Vitis
Heuchera
Malus
Prunus
Ziziphus
Cannabis
Morus
Spondias
Acer
Euonymous
Cucurbita
Epirixanthes
Tamarindus
Acacia
Vigna
Lotus
Medinilla
Lagerstroemia
Eucalyptus
Arabidopsis
Carica
Cytinus
Betula
Alnus
Morella
Juglans
Fagus
Quercus
Lithocarpus
Castanea
Castanopsis
Gossypium
Malva sylvestris
Hibiscus cannabinus
Erythropalum
Malania
Santalum
Tolypanthus
Amyema
Camellia
Mitrastemon
Actinidia
Ilex
Daucus
Panax
Lactuca
Helianthus
Chrysanthemum
Aegiceras
Cuscuta
Nicotiana
Capsicum annuum
Solanum melongena
Ombrophytum
Ricinus
Chrysobalanus
Bruguiera
Salix
Passiflora
Glycine
Geranium
Platycodon
Citrus
Rhododendron
Vaccinium
Monotropa
Hypopitys
Fallopia
Nepenthes
Mirabilis
Sesuvium
Beta
Silene
Rhazya
Morinda
Gardenia
Hesperelaea
Salvia
Boea
Utricularia
Mimulus
Castilleja
Tree scale: 0.01

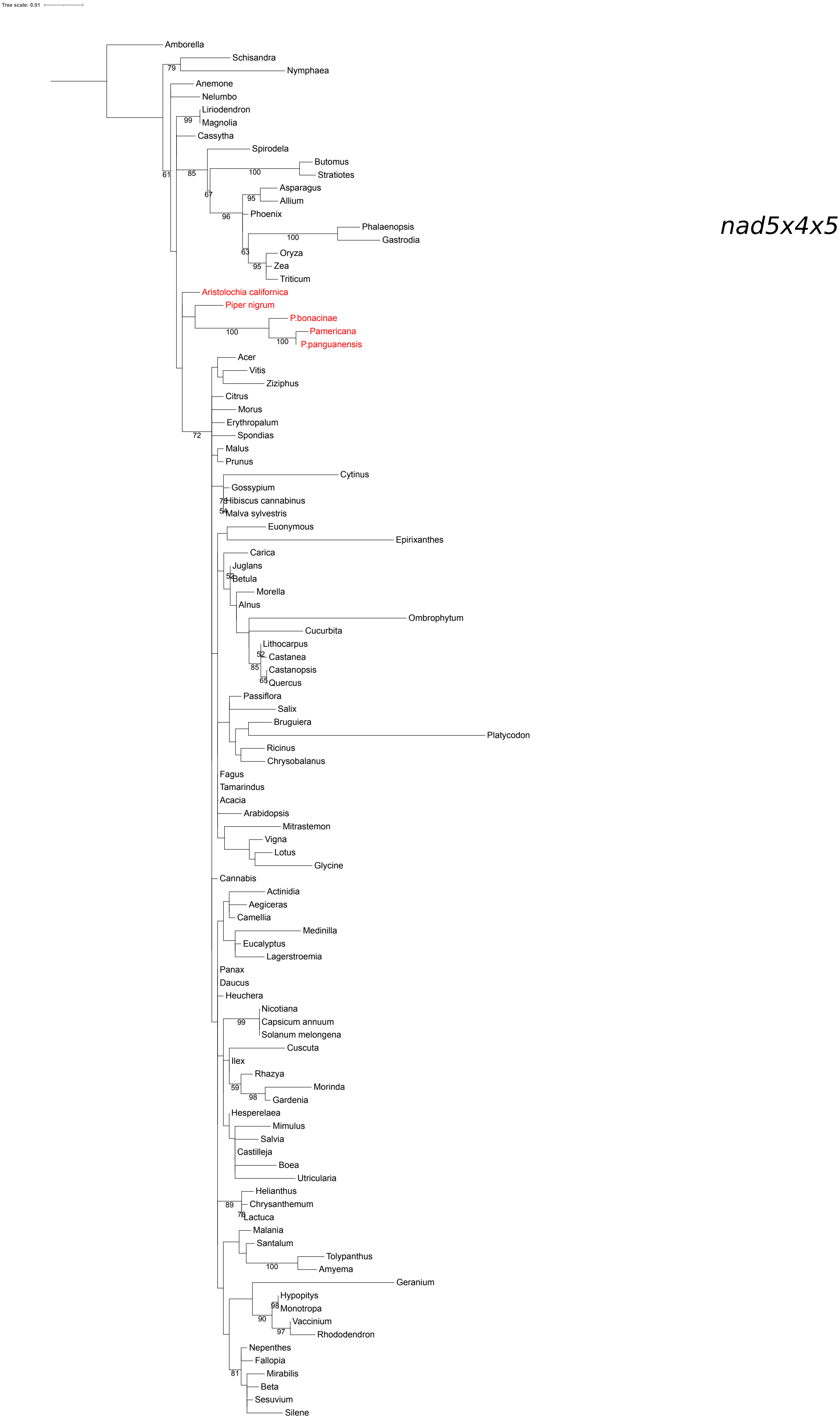

Tree scale: 0.01
nad5x4x5
Amborella
Schisandra
Nymphaea
Anemone
Nelumbo
Liriodendron
Magnolia
Cassytha
Spirodela
Butomus
Stratiotes
Asparagus
Allium
Phoenix
Phalaenopsis
Gastrodia
Oryza
Zea
Triticum
Aristolochia californica
Piper nigrum
P.bonacinae
Pamericana
P.panguanensis
Acer
Vitis
Ziziphus
Citrus
Morus
Erythropalum
Spondias
Malus
Prunus
Cytinus
Gossypium
Hibiscus cannabinus
Malva sylvestris
Euonymous
Epirixanthes
Carica
Juglans
Betula
Morella
Alnus
Ombrophytum
Cucurbita
Lithocarpus
Castanea
Castanopsis
Quercus
Passiflora
Salix
Bruguiera
Platycodon
Ricinus
Chrysobalanus
Fagus
Tamarindus
Acacia
Arabidopsis
Mitrastemon
Vigna
Lotus
Glycine
Cannabis
Actinidia
Aegiceras
Camellia
Medinilla
Eucalyptus
Lagerstroemia
Panax
Daucus
Heuchera
Nicotiana
Capsicum annuum
Solanum melongena
Cuscuta
Ilex
Rhazya
Morinda
Gardenia
Hesperelaea
Mimulus
Salvia
Castilleja
Boea
Utricularia
Helianthus
Chrysanthemum
Lactuca
Malania
Santalum
Tolypanthus
Amyema
Geranium
Hypopitys
Monotropa
Vaccinium
Rhododendron
Nepenthes
Fallopia
Mirabilis
Beta
Sesuvium
Silene

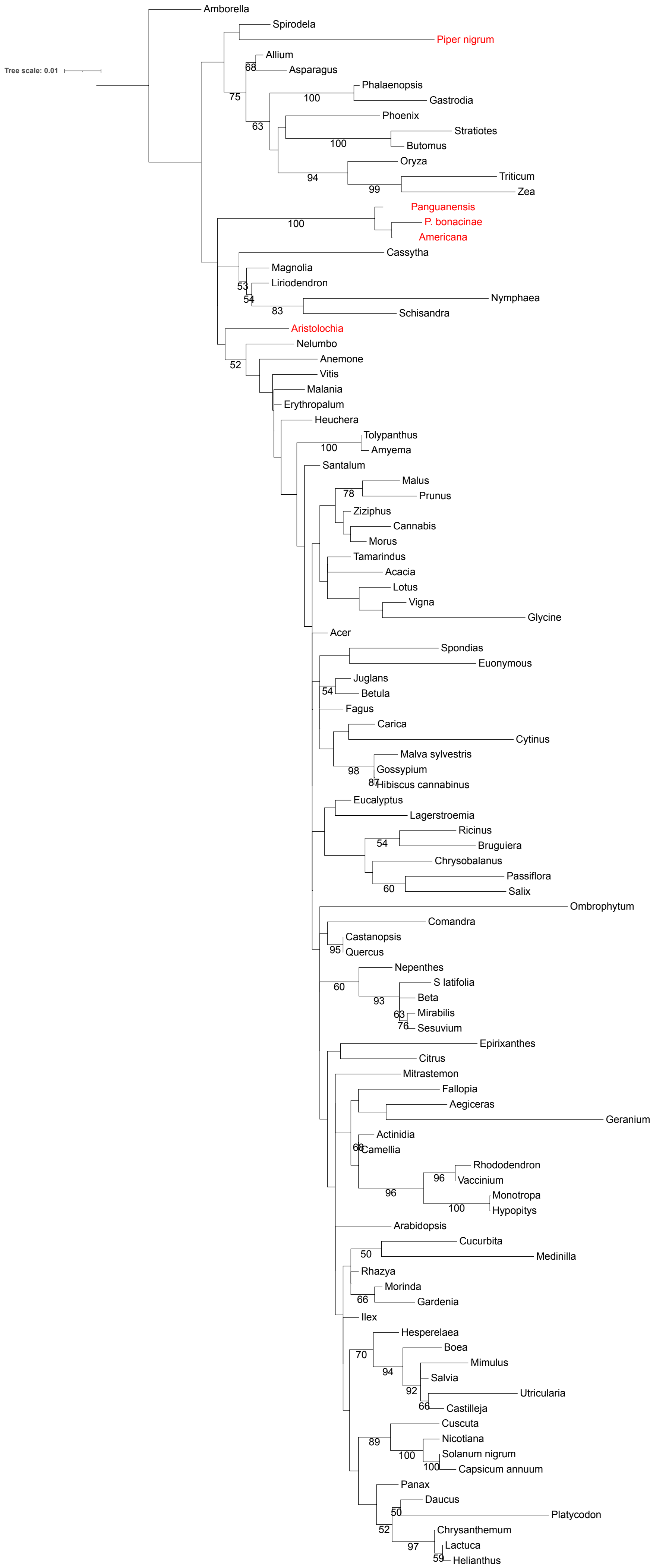

nad6
Tree scale: 0.01
Amborella
Spirodela
Piper nigrum
Allium
Asparagus
Phalaenopsis
Gastrodia
Phoenix
Stratiotes
Butomus
Oryza
Triticum
Zea
Panguanensis
P. bonacinae
Americana
Cassytha
Magnolia
Liriodendron
Nymphaea
Schisandra
Aristolochia
Nelumbo
Anemone
Vitis
Malania
Erythropalum
Heuchera
Tolypanthus
Amyema
Santalum
Malus
Prunus
Ziziphus
Cannabis
Morus
Tamarindus
Acacia
Lotus
Vigna
Glycine
Acer
Spondias
Euonymous
Juglans
Betula
Fagus
Carica
Cytinus
Malva sylvestris
Gossypium
Hibiscus cannabinus
Eucalyptus
Lagerstroemia
Ricinus
Bruguiera
Chrysobalanus
Passiflora
Salix
Ombrophytum
Comandra
Castanopsis
Quercus
Nepenthes
S latifolia
Beta
Mirabilis
Sesuvium
Epirixanthes
Citrus
Mitrastemon
Fallopia
Aegiceras
Geranium
Actinidia
Camellia
Rhododendron
Vaccinium
Monotropa
Hypopitys
Arabidopsis
Cucurbita
Medinilla
Rhazya
Morinda
Gardenia
Ilex
Hesperelaea
Boea
Mimulus
Salvia
Utricularia
Castilleja
Cuscuta
Nicotiana
Solanum nigrum
Capsicum annuum
Panax
Daucus
Platycodon
Chrysanthemum
Lactuca
Helianthus

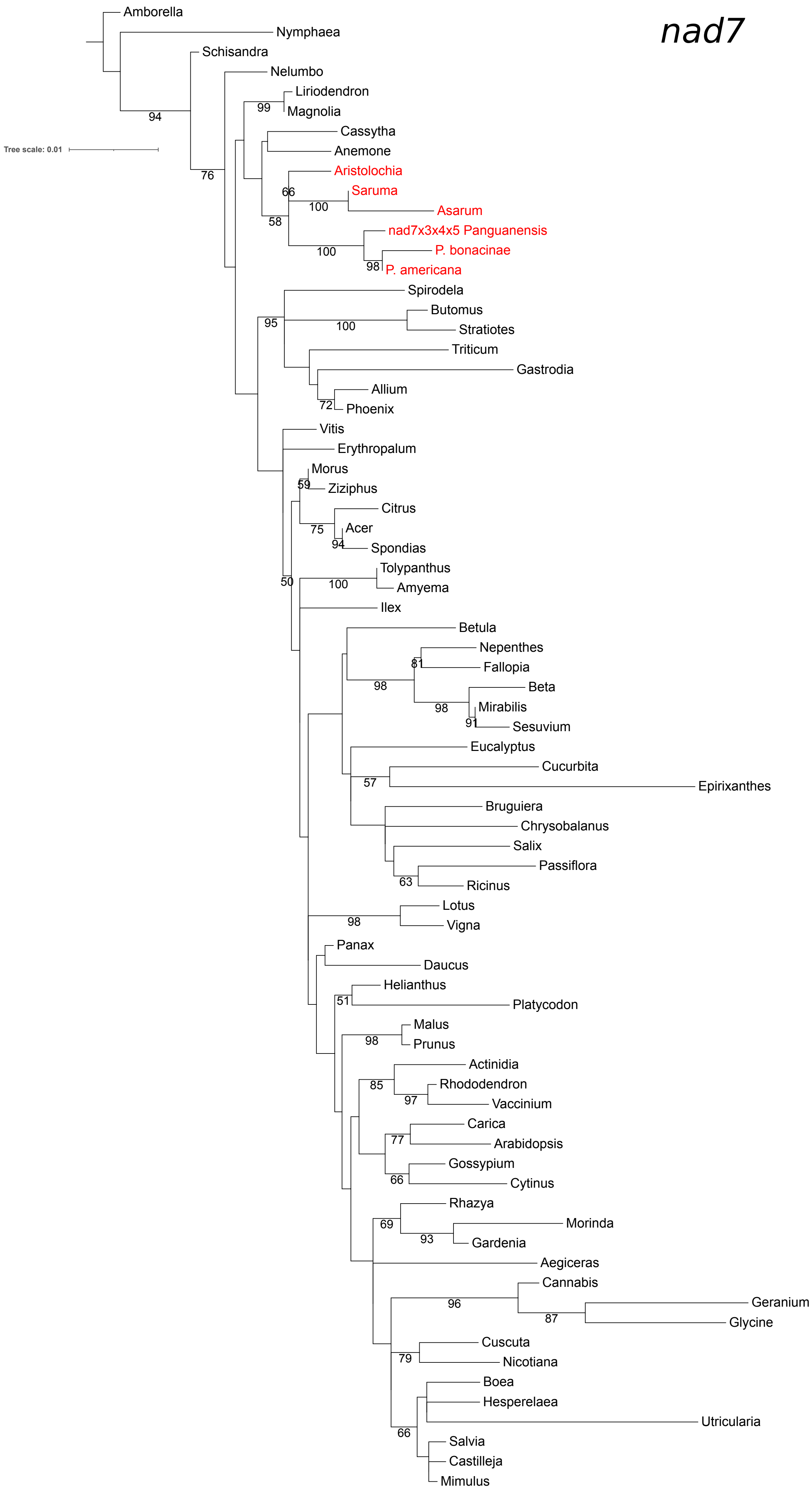
nad7
Tree scale: 0.01
Amborella
Nymphaea
Schisandra
94
Nelumbo
Liriodendron
99
Magnolia
Cassytha
Anemone
76
Aristolochia
66
Saruma
100
Asarum
58
nad7x3x4x5 Panguanensis
100
P. bonacinae
98
P. americana
Spirodela
Butomus
95
100
Stratiotes
Triticum
Gastrodia
Allium
72
Phoenix
Vitis
Erythropalum
Morus
59
Ziziphus
Citrus
75
Acer
94
Spondias
Tolypanthus
50
100
Amyema
Ilex
Betula
Nepenthes
81
Fallopia
98
Beta
98
Mirabilis
91
Sesuvium
Eucalyptus
Cucurbita
57
Epirixanthes
Bruguiera
Chrysobalanus
Salix
Passiflora
63
Ricinus
Lotus
98
Vigna
Panax
Daucus
Helianthus
51
Platycodon
Malus
98
Prunus
Actinidia
85
Rhododendron
97
Vaccinium
Carica
77
Arabidopsis
Gossypium
66
Cytinus
Rhazya
69
Morinda
93
Gardenia
Aegiceras
Cannabis
96
Geranium
87
Glycine
Cuscuta
79
Nicotiana
Boea
Hesperelaea
Utricularia
66
Salvia
Castilleja
Mimulus

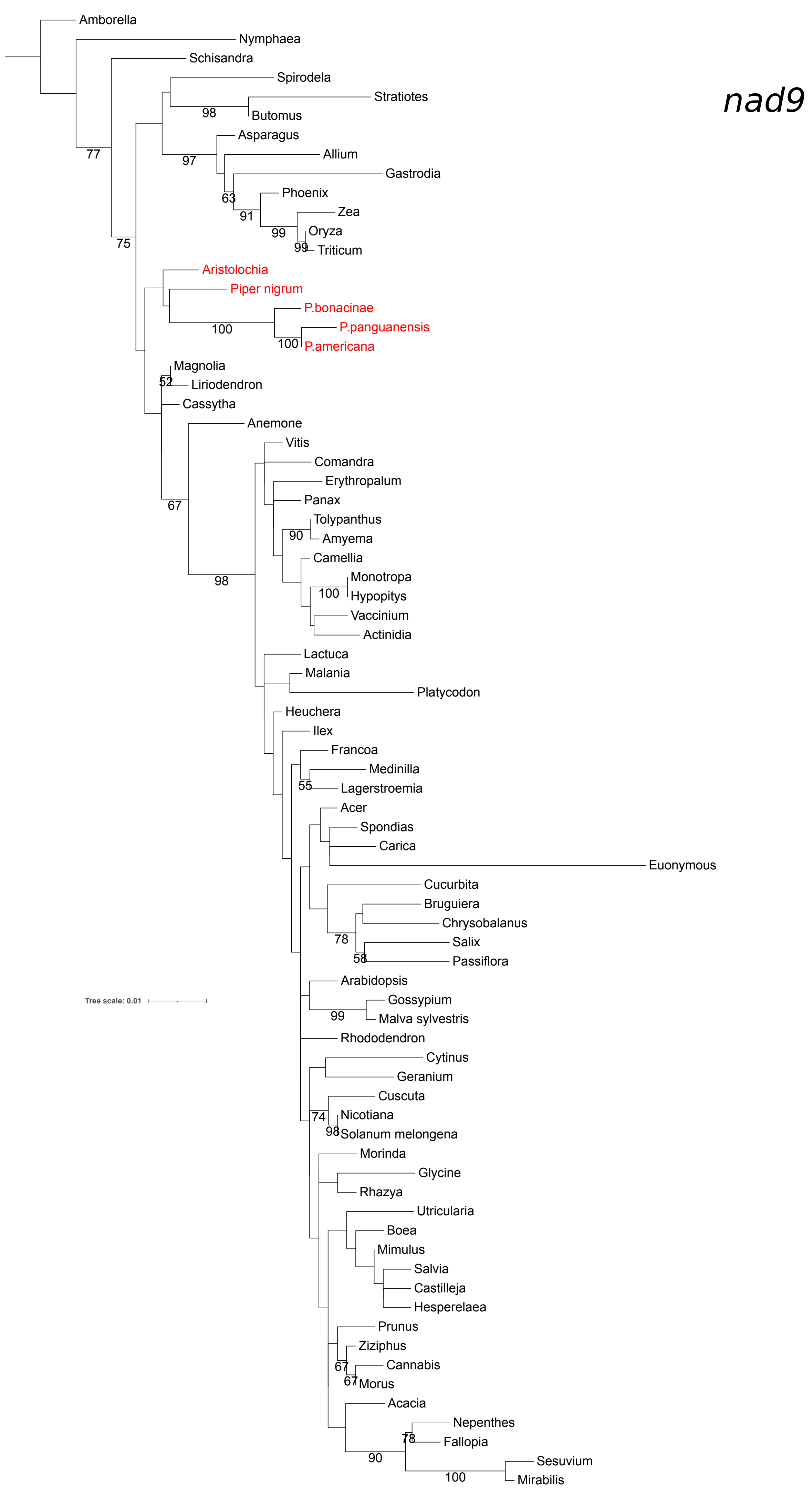

nad9
Amborella
Nymphaea
Schisandra
Spirodela
Stratiotes
Butomus
Asparagus
Allium
Gastrodia
Phoenix
Zea
Oryza
Triticum
Aristolochia
Piper nigrum
P.bonacinae
P.panguanensis
P.americana
Magnolia
Liriodendron
Cassytha
Anemone
Vitis
Comandra
Erythropalum
Panax
Tolypanthus
Amyema
Camellia
Monotropa
Hypopitys
Vaccinium
Actinidia
Lactuca
Malania
Platycodon
Heuchera
Ilex
Francoa
Medinilla
Lagerstroemia
Acer
Spondias
Carica
Euonymous
Cucurbita
Bruguiera
Chrysobalanus
Salix
Passiflora
Arabidopsis
Gossypium
Malva sylvestris
Rhododendron
Cytinus
Geranium
Cuscuta
Nicotiana
Solanum melongena
Morinda
Glycine
Rhazya
Utricularia
Boea
Mimulus
Salvia
Castilleja
Hesperelaea
Prunus
Ziziphus
Cannabis
Morus
Acacia
Nepenthes
Fallopia
Sesuvium
Mirabilis
Tree scale: 0.01

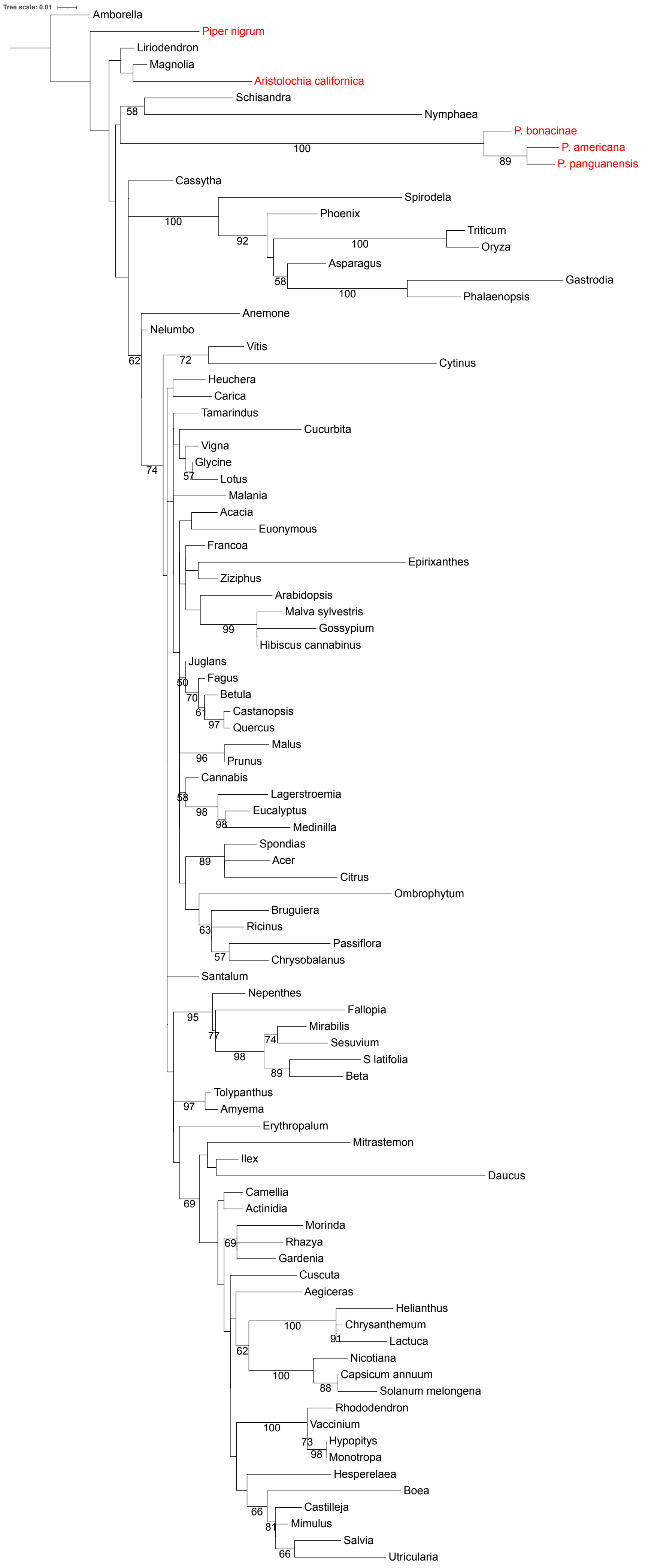
Tree scale: 0.01
Amborella
Piper nigrum
Liriodendron
Magnolia
Aristolochia californica
Schisandra
Nymphaea
P. bonacinae
P. americana
P. panguanensis
Cassytha
Spirodela
Phoenix
Triticum
Oryza
Asparagus
Gastrodia
Phalaenopsis
Anemone
Nelumbo
Vitis
Cytinus
Heuchera
Carica
Tamarindus
Cucurbita
Vigna
Glycine
Lotus
Malania
Acacia
Euonymous
Francoa
Epirixanthes
Ziziphus
Arabidopsis
Malva sylvestris
Gossypium
Hibiscus cannabinus
Juglans
Fagus
Betula
Castanopsis
Quercus
Malus
Prunus
Cannabis
Lagerstroemia
Eucalyptus
Medinilla
Spondias
Acer
Citrus
Ombrophytum
Bruguiera
Ricinus
Passiflora
Chrysobalanus
Santalum
Nepenthes
Fallopia
Mirabilis
Sesuvium
S latifolia
Beta
Tolypanthus
Amyema
Erythropalum
Mitrastemon
Ilex
Daucus
Camellia
Actinidia
Morinda
Rhazya
Gardenia
Cuscuta
Aegiceras
Helianthus
Chrysanthemum
Lactuca
Nicotiana
Capsicum annuum
Solanum melongena
Rhododendron
Vaccinium
Hypopitys
Monotropa
Hesperelaea
Boea
Castilleja
Mimulus
Salvia
Utricularia

rpl5

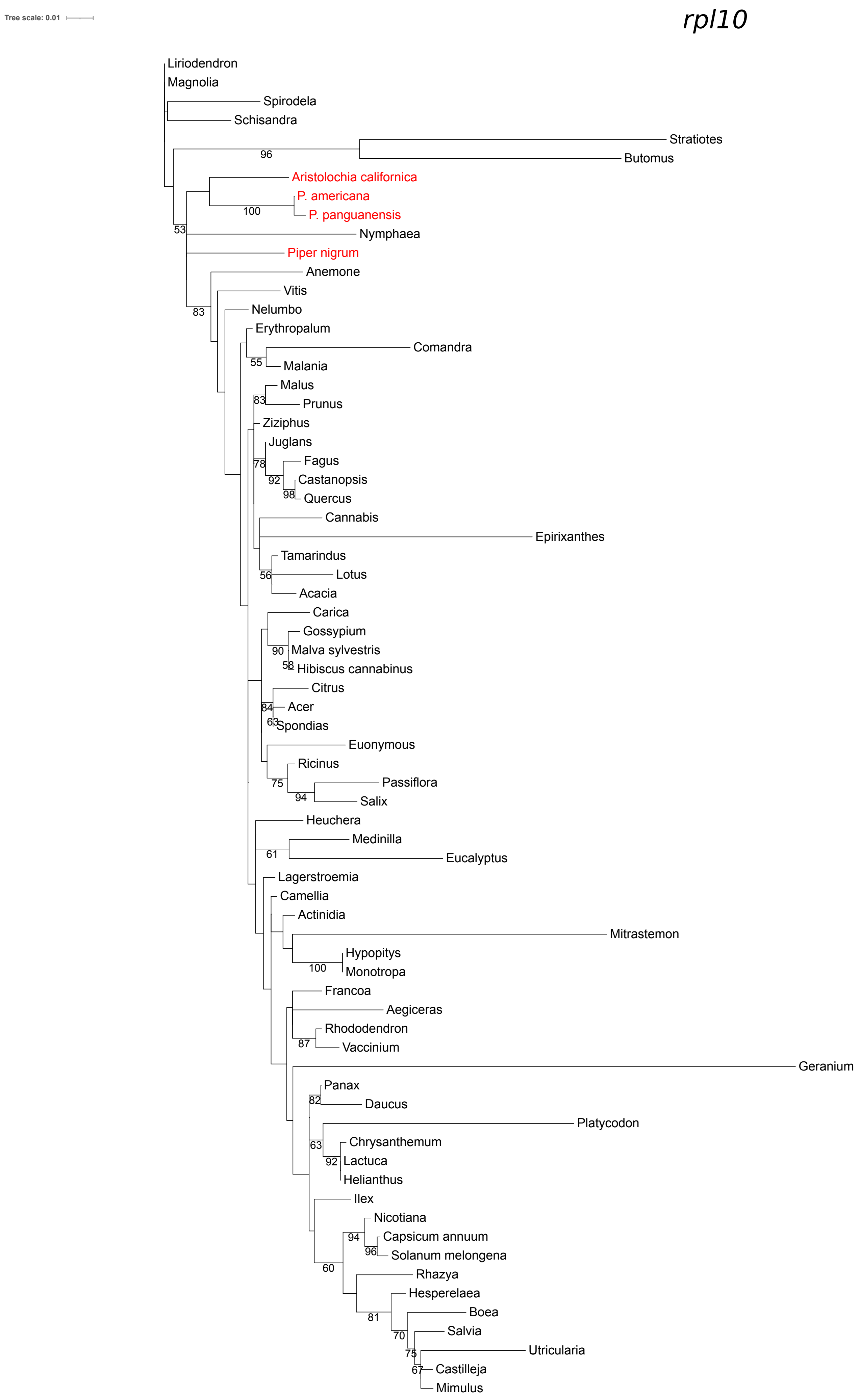
Tree scale: 0.01
rpl10
Liriodendron
Magnolia
Spirodela
Schisandra
Stratiotes
96
Butomus
Aristolochia californica
P. americana
100
P. panguanensis
53
Nymphaea
Piper nigrum
Anemone
Vitis
83
Nelumbo
Erythropalum
Comandra
55
Malania
Malus
83
Prunus
Ziziphus
Juglans
78
Fagus
92
Castanopsis
98
Quercus
Cannabis
Epirixanthes
Tamarindus
56
Lotus
Acacia
Carica
Gossypium
90
Malva sylvestris
58
Hibiscus cannabinus
Citrus
84
Acer
63
Spondias
Euonymous
Ricinus
75
Passiflora
94
Salix
Heuchera
Medinilla
61
Eucalyptus
Lagerstroemia
Camellia
Actinidia
Mitrastemon
Hypopitys
100
Monotropa
Francoa
Aegiceras
Rhododendron
87
Vaccinium
Geranium
Panax
82
Daucus
Platycodon
63
Chrysanthemum
92
Lactuca
Helianthus
Ilex
Nicotiana
94
Capsicum annuum
96
Solanum melongena
60
Rhazya
Hesperelaea
81
Boea
70
Salvia
75
Utricularia
67
Castilleja
Mimulus

rpl16

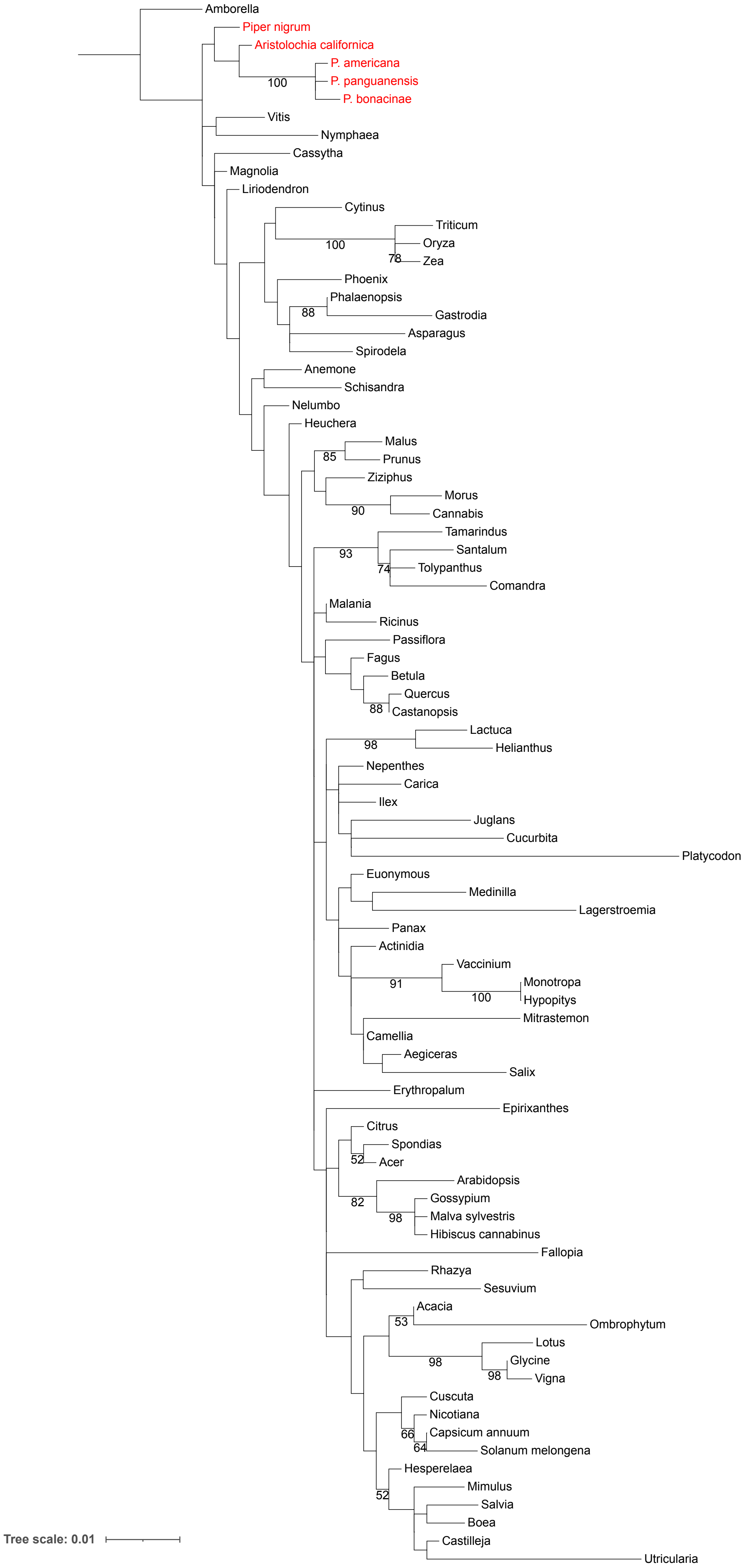

Amborella
Piper nigrum
Aristolochia californica
P. americana
P. panguanensis
P. bonacinae
100
Vitis
Nymphaea
Cassytha
Magnolia
Liriodendron
Cytinus
Triticum
Oryza
Zea
100
78
Phoenix
Phalaenopsis
Gastrodia
88
Asparagus
Spirodela
Anemone
Schisandra
Nelumbo
Heuchera
Malus
Prunus
85
Ziziphus
Morus
Cannabis
90
Tamarindus
Santalum
93
Tolypanthus
74
Comandra
Malania
Ricinus
Passiflora
Fagus
Betula
Quercus
Castanopsis
88
Lactuca
Helianthus
98
Nepenthes
Carica
Ilex
Juglans
Cucurbita
Platycodon
Euonymous
Medinilla
Lagerstroemia
Panax
Actinidia
Vaccinium
Monotropa
Hypopitys
91
100
Mitrastemon
Camellia
Aegiceras
Salix
Erythropalum
Epirixanthes
Citrus
Spondias
Acer
52
Arabidopsis
Gossypium
Malva sylvestris
Hibiscus cannabinus
82
98
Fallopia
Rhazya
Sesuvium
Acacia
53
Ombrophytum
Lotus
Glycine
Vigna
98
98
Cuscuta
Nicotiana
Capsicum annuum
Solanum melongena
66
64
Hesperelaea
Mimulus
Salvia
Boea
52
Castilleja
Utricularia
Tree scale: 0.01

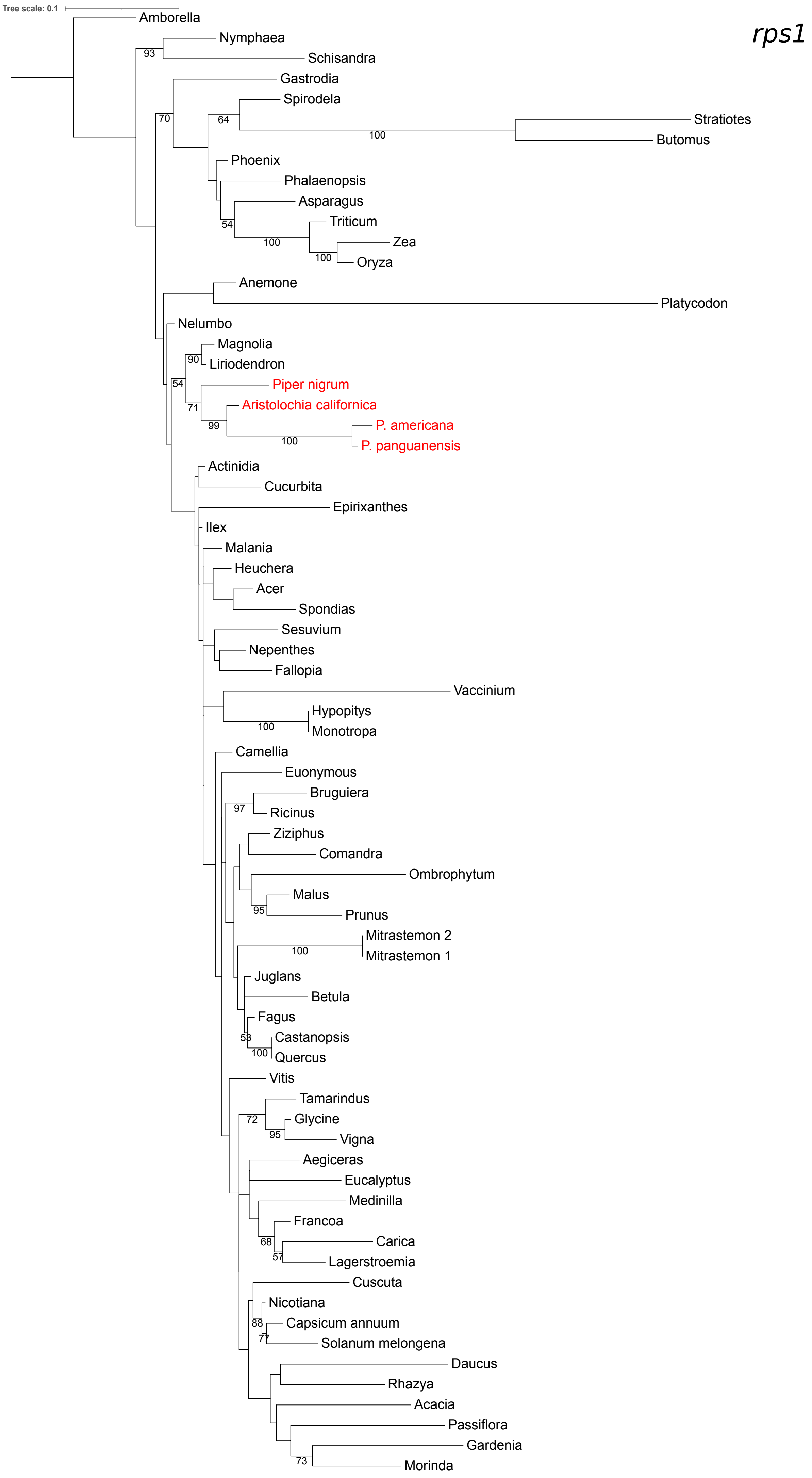
Tree scale: 0.1
rps1
Amborella
Nymphaea
Schisandra
Gastrodia
Spirodela
Stratiotes
Butomus
Phoenix
Phalaenopsis
Asparagus
Triticum
Zea
Oryza
Anemone
Platycodon
Nelumbo
Magnolia
Liriodendron
Piper nigrum
Aristolochia californica
P. americana
P. panguanensis
Actinidia
Cucurbita
Epirixanthes
Ilex
Malania
Heuchera
Acer
Spondias
Sesuvium
Nepenthes
Fallopia
Vaccinium
Hypopitys
Monotropa
Camellia
Euonymous
Bruguiera
Ricinus
Ziziphus
Comandra
Ombrophytum
Malus
Prunus
Mitrastemon 2
Mitrastemon 1
Juglans
Betula
Fagus
Castanopsis
Quercus
Vitis
Tamarindus
Glycine
Vigna
Aegiceras
Eucalyptus
Medinilla
Francoa
Carica
Lagerstroemia
Cuscuta
Nicotiana
Capsicum annuum
Solanum melongena
Daucus
Rhazya
Acacia
Passiflora
Gardenia
Morinda

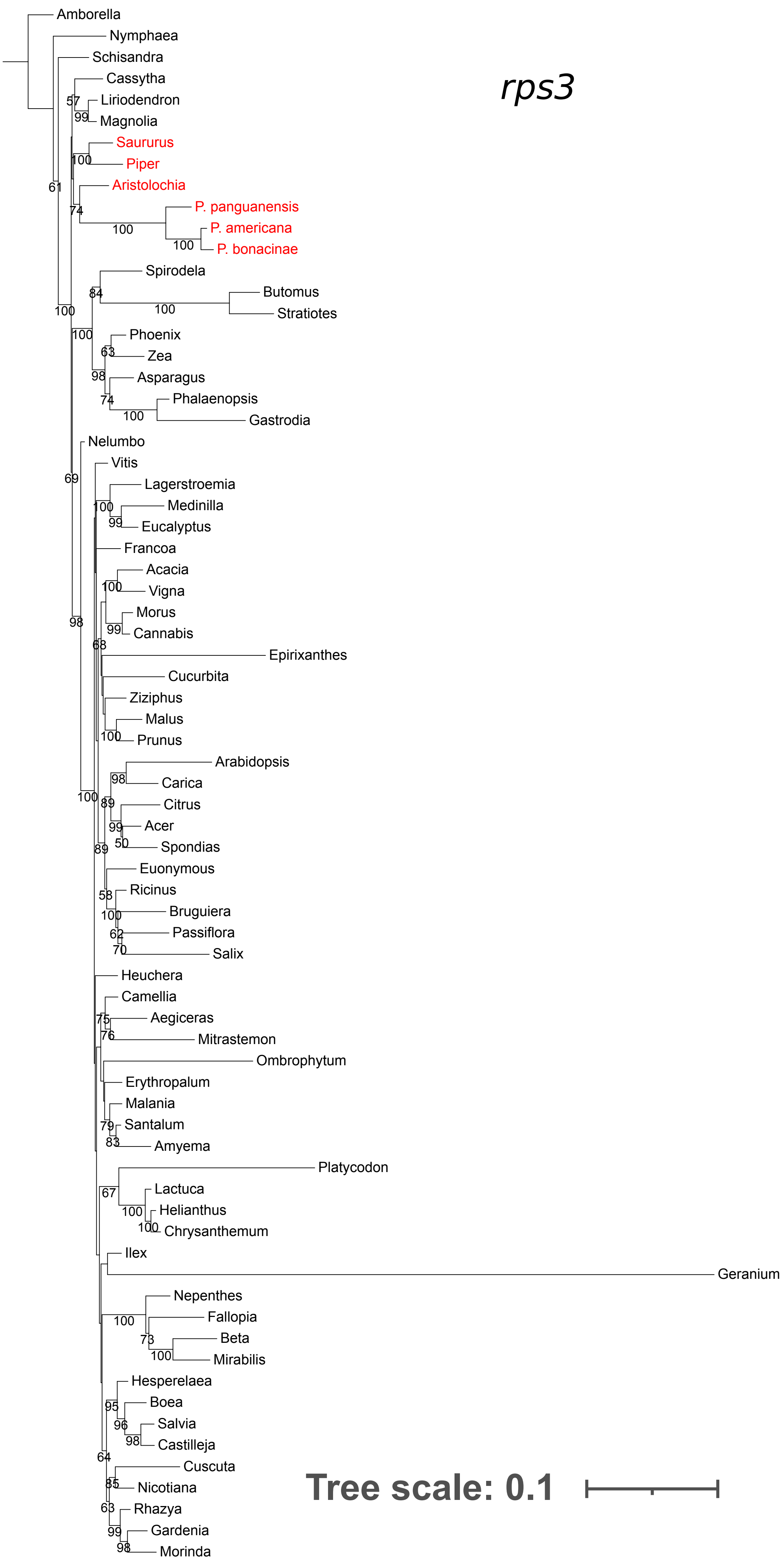

rps3
Amborella
Nymphaea
Schisandra
Cassytha
Liriodendron
Magnolia
Saururus
Piper
Aristolochia
P. panguanensis
P. americana
P. bonacinae
Spirodela
Butomus
Stratiotes
Phoenix
Zea
Asparagus
Phalaenopsis
Gastrodia
Nelumbo
Vitis
Lagerstroemia
Medinilla
Eucalyptus
Francoa
Acacia
Vigna
Morus
Cannabis
Epirixanthes
Cucurbita
Ziziphus
Malus
Prunus
Arabidopsis
Carica
Citrus
Acer
Spondias
Euonymous
Ricinus
Bruguiera
Passiflora
Salix
Heuchera
Camellia
Aegiceras
Mitrastemon
Ombrophytum
Erythropalum
Malania
Santalum
Amyema
Platycodon
Lactuca
Helianthus
Chrysanthemum
Ilex
Geranium
Nepenthes
Fallopia
Beta
Mirabilis
Hesperelaea
Boea
Salvia
Castilleja
Cuscuta
Nicotiana
Rhazya
Gardenia
Morinda
Tree scale: 0.1

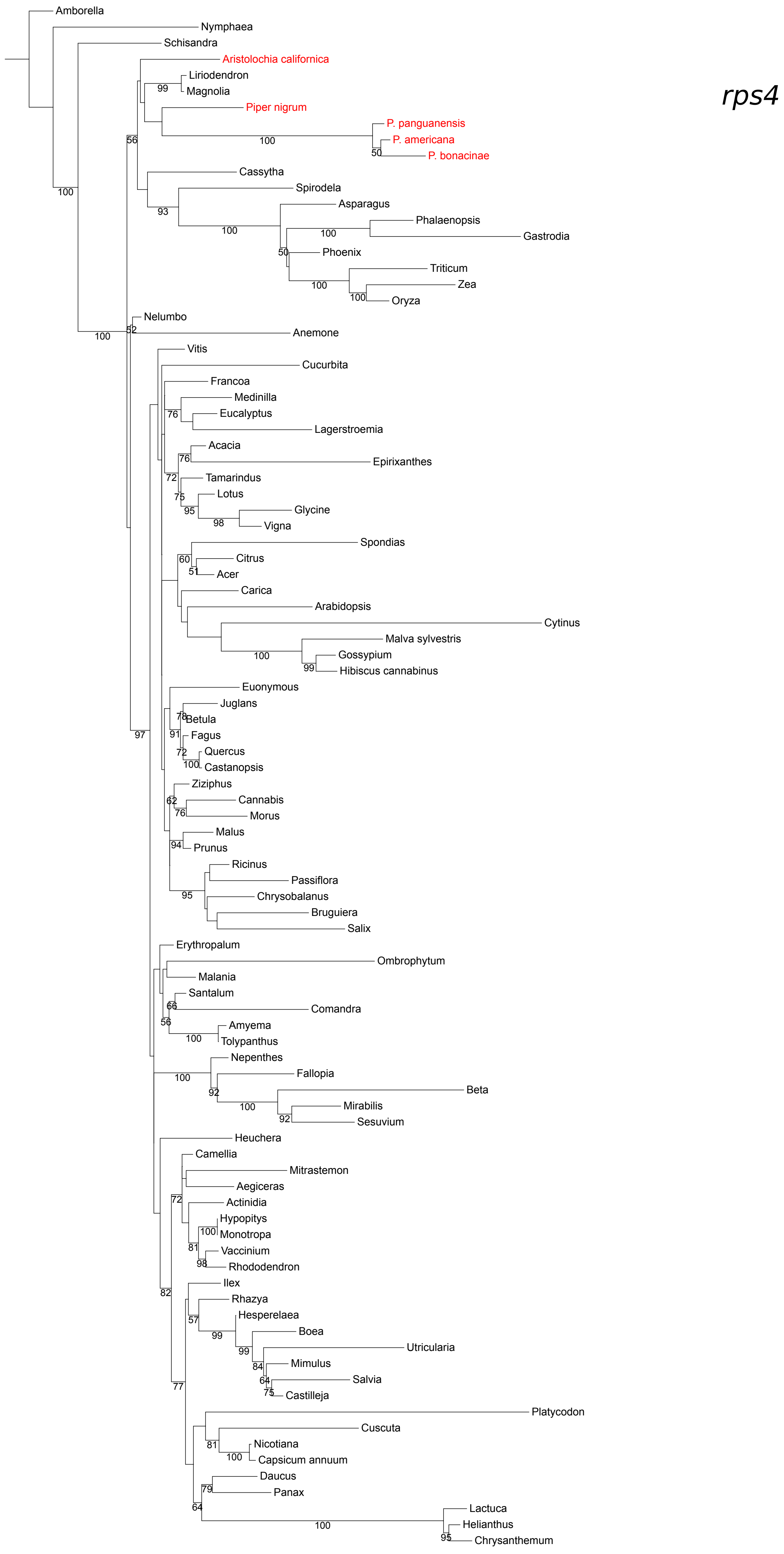

rps4
Amborella
Nymphaea
Schisandra
Aristolochia californica
Liriodendron
Magnolia
Piper nigrum
P. panguanensis
P. americana
P. bonacinae
Cassytha
Spirodela
Asparagus
Phalaenopsis
Gastrodia
Phoenix
Triticum
Zea
Oryza
Nelumbo
Anemone
Vitis
Cucurbita
Francoa
Medinilla
Eucalyptus
Lagerstroemia
Acacia
Epirixanthes
Tamarindus
Lotus
Glycine
Vigna
Spondias
Citrus
Acer
Carica
Arabidopsis
Cytinus
Malva sylvestris
Gossypium
Hibiscus cannabinus
Euonymous
Juglans
Betula
Fagus
Quercus
Castanopsis
Ziziphus
Cannabis
Morus
Malus
Prunus
Ricinus
Passiflora
Chrysobalanus
Bruguiera
Salix
Erythropalum
Ombrophytum
Malania
Santalum
Comandra
Amyema
Tolypanthus
Nepenthes
Fallopia
Beta
Mirabilis
Sesuvium
Heuchera
Camellia
Mitrastemon
Aegiceras
Actinidia
Hypopitys
Monotropa
Vaccinium
Rhododendron
Ilex
Rhazya
Hesperelaea
Boea
Utricularia
Mimulus
Salvia
Castilleja
Platycodon
Cuscuta
Nicotiana
Capsicum annuum
Daucus
Panax
Lactuca
Helianthus
Chrysanthemum

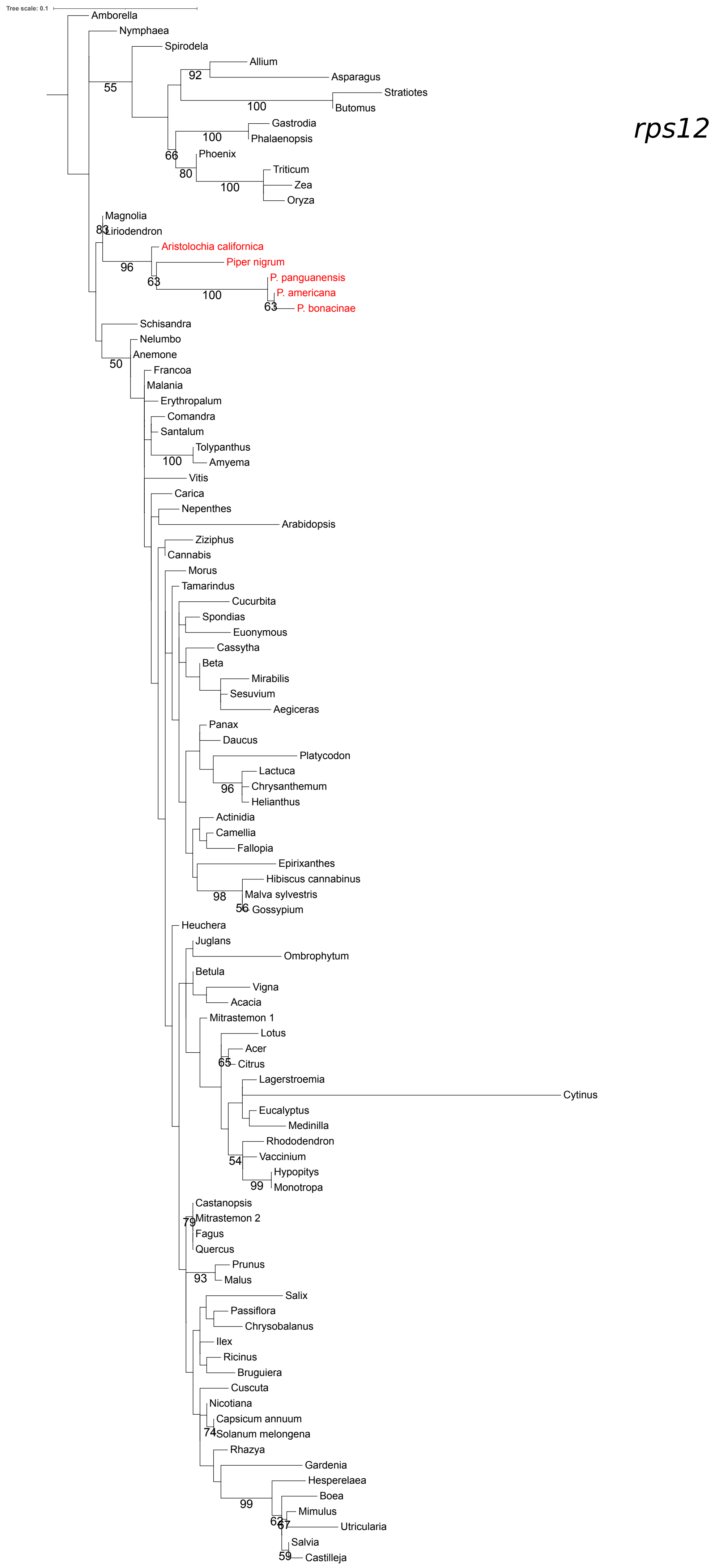
Tree scale: 0.1
rps12
Amborella
Nymphaea
Spirodela
Allium
Asparagus
Stratiotes
Butomus
Gastrodia
Phalaenopsis
Phoenix
Triticum
Zea
Oryza
Magnolia
Liriodendron
Aristolochia californica
Piper nigrum
P. panguanensis
P. americana
P. bonacinae
Schisandra
Nelumbo
Anemone
Francoa
Malania
Erythropalum
Comandra
Santalum
Tolypanthus
Amyema
Vitis
Carica
Nepenthes
Arabidopsis
Ziziphus
Cannabis
Morus
Tamarindus
Cucurbita
Spondias
Euonymous
Cassytha
Beta
Mirabilis
Sesuvium
Aegiceras
Panax
Daucus
Platycodon
Lactuca
Chrysanthemum
Helianthus
Actinidia
Camellia
Fallopia
Epirixanthes
Hibiscus cannabinus
Malva sylvestris
Gossypium
Heuchera
Juglans
Ombrophytum
Betula
Vigna
Acacia
Mitrastemon 1
Lotus
Acer
Citrus
Lagerstroemia
Cytinus
Eucalyptus
Medinilla
Rhododendron
Vaccinium
Hypopitys
Monotropa
Castanopsis
Mitrastemon 2
Fagus
Quercus
Prunus
Malus
Salix
Passiflora
Chrysobalanus
Ilex
Ricinus
Bruguiera
Cuscuta
Nicotiana
Capsicum annuum
Solanum melongena
Rhazya
Gardenia
Hesperelaea
Boea
Mimulus
Utricularia
Salvia
Castilleja

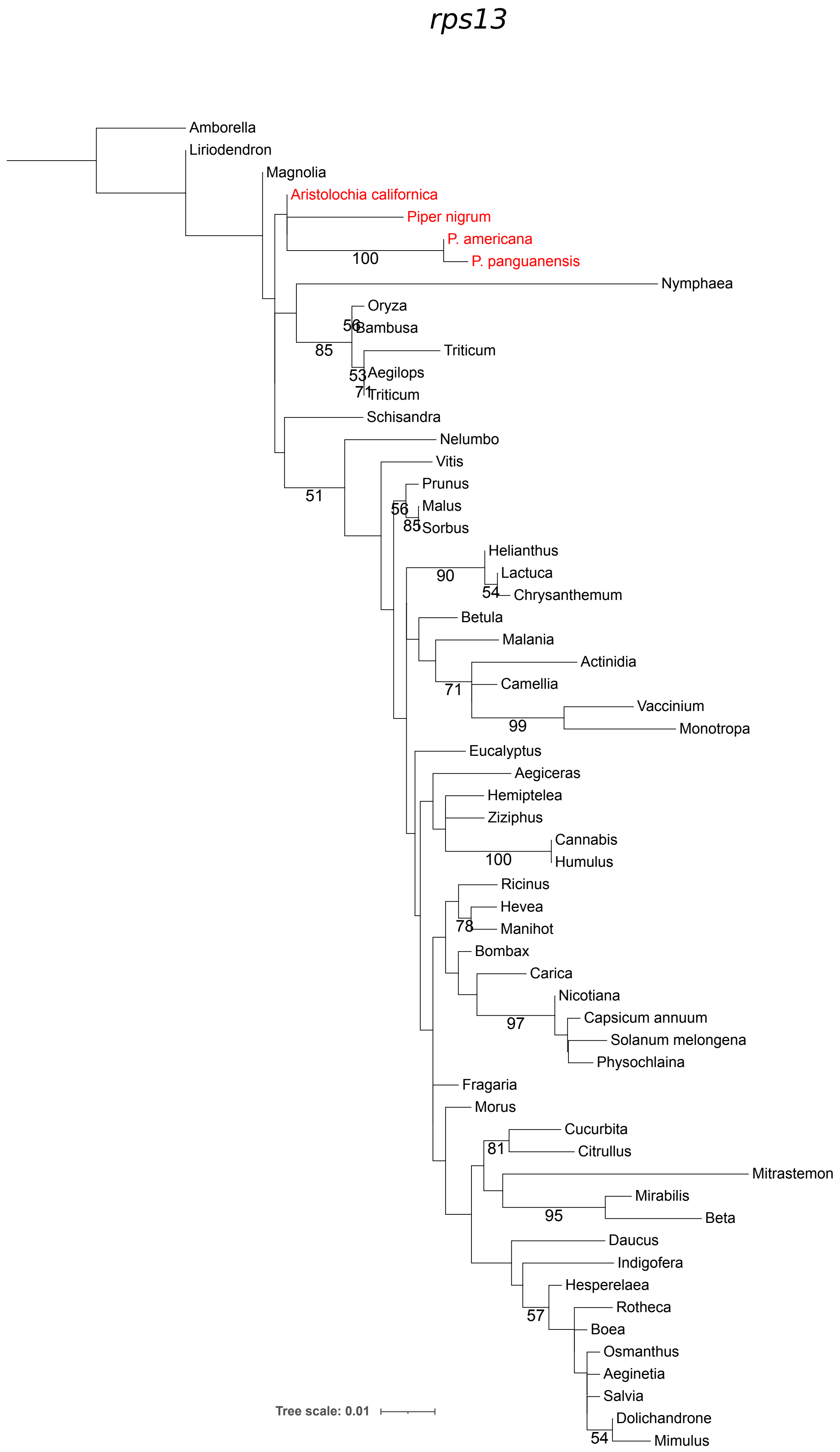
rps13
Amborella
Liriodendron
Magnolia
Aristolochia californica
Piper nigrum
P. americana
100
P. panguanensis
Nymphaea
Oryza
56
Bambusa
85
Triticum
53
Aegilops
71
Triticum
Schisandra
Nelumbo
Vitis
51
Prunus
56
Malus
85
Sorbus
Helianthus
90
Lactuca
54
Chrysanthemum
Betula
Malania
Actinidia
71
Camellia
Vaccinium
99
Monotropa
Eucalyptus
Aegiceras
Hemiptelea
Ziziphus
Cannabis
100
Humulus
Ricinus
Hevea
78
Manihot
Bombax
Carica
Nicotiana
Capsicum annuum
97
Solanum melongena
Physochlaina
Fragaria
Morus
Cucurbita
81
Citrullus
Mitrastemon
Mirabilis
95
Beta
Daucus
Indigofera
Hesperelaea
Rotheca
57
Boea
Osmanthus
Aeginetia
Salvia
Dolichandrone
54
Mimulus
Tree scale: 0.01

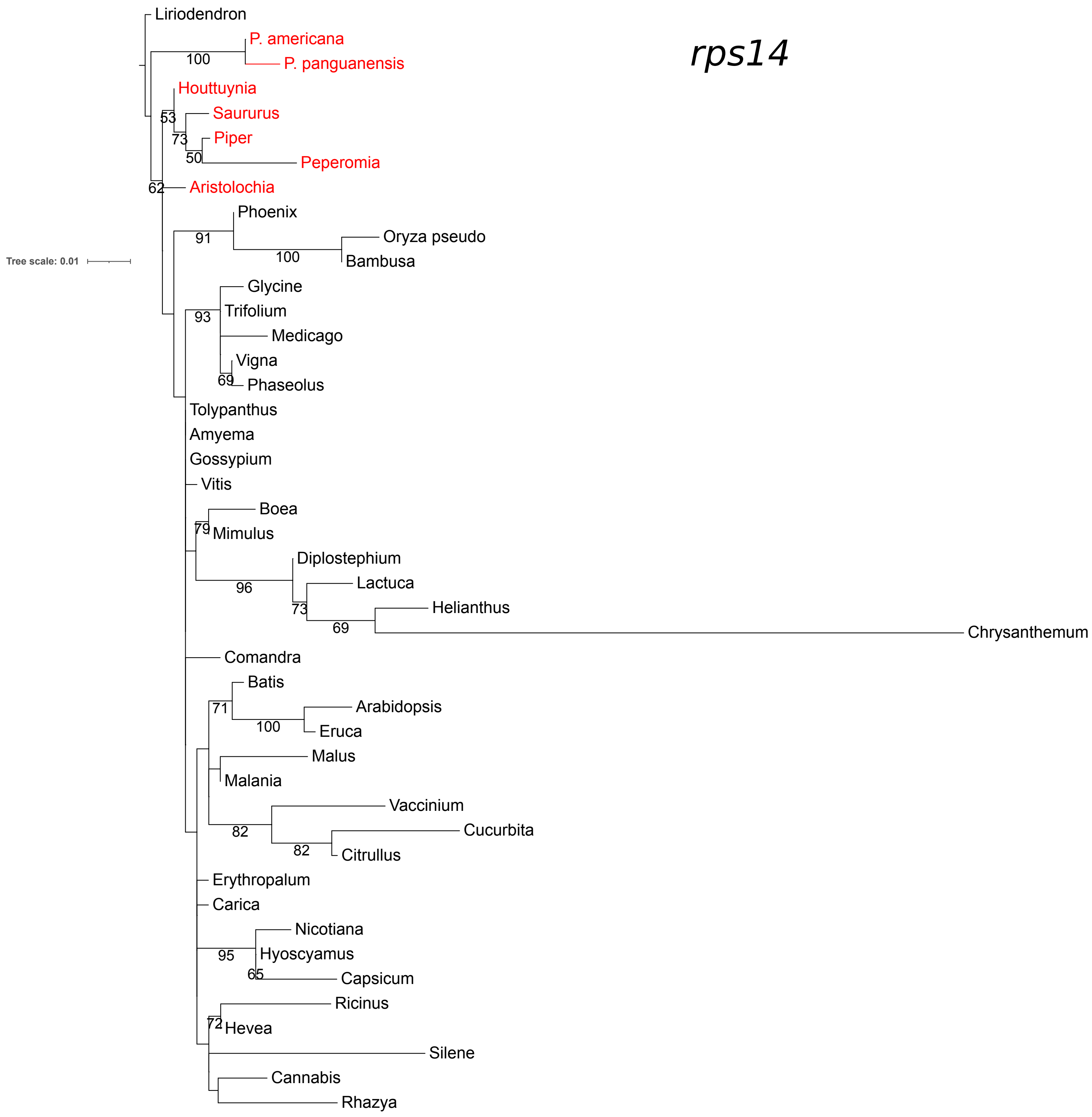
rps14
Tree scale: 0.01
Liriodendron
P. americana
P. panguanensis
100
Houttuynia
Saururus
Piper
Peperomia
53
73
50
62
Aristolochia
Phoenix
Oryza pseudo
Bambusa
91
100
Glycine
Trifolium
Medicago
Vigna
Phaseolus
93
69
Tolypanthus
Amyema
Gossypium
Vitis
Boea
Mimulus
79
Diplostephium
Lactuca
Helianthus
Chrysanthemum
96
73
69
Comandra
Batis
Arabidopsis
Eruca
71
100
Malus
Malania
Vaccinium
Cucurbita
Citrullus
82
82
Erythropalum
Carica
Nicotiana
Hyoscyamus
Capsicum
95
65
Ricinus
Hevea
72
Silene
Cannabis
Rhazya

sdh3

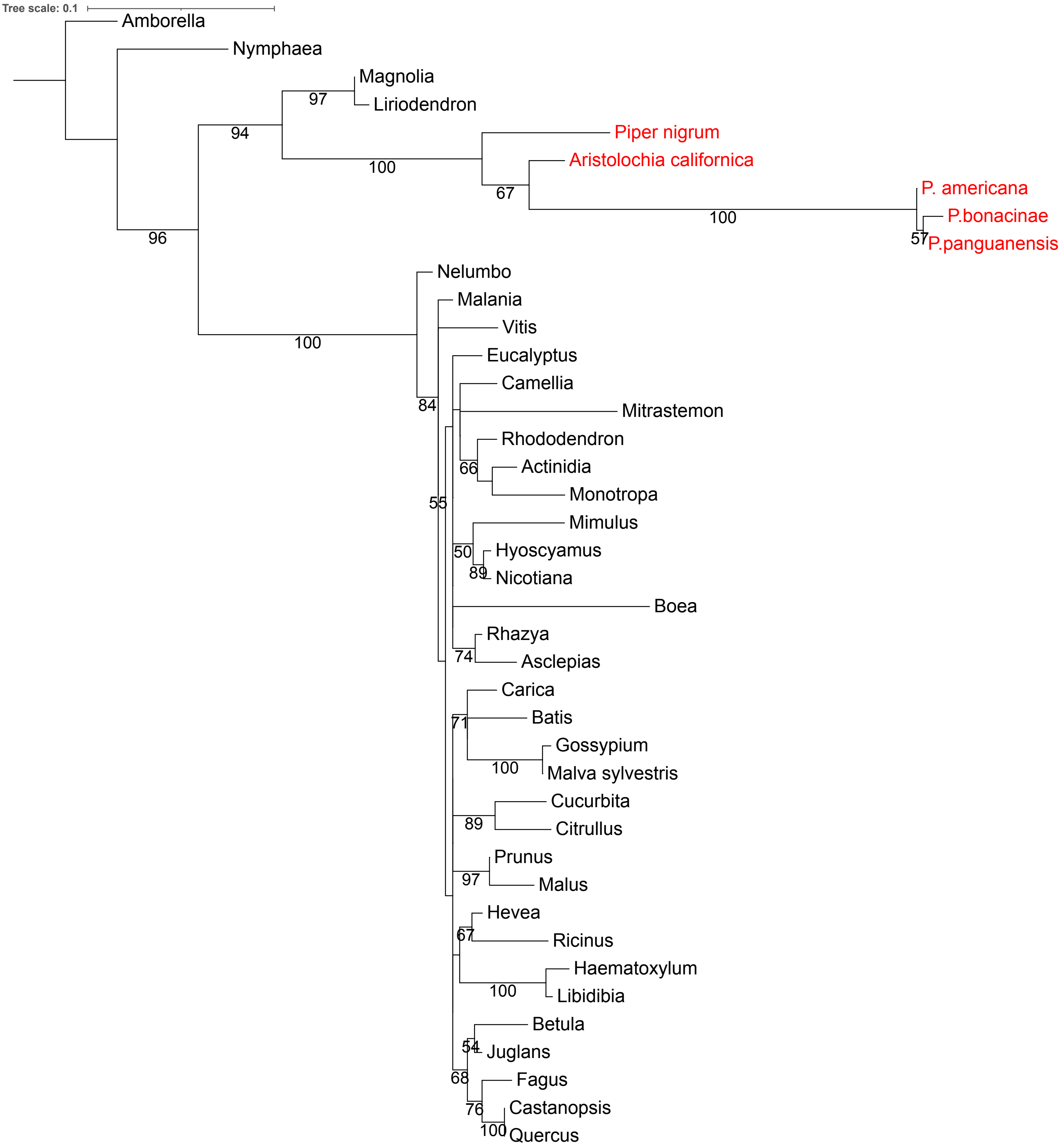
Tree scale: 0.1
Amborella
Nymphaea
Magnolia
97
Liriodendron
Piper nigrum
94
100
Aristolochia californica
67
P. americana
100
P.bonacinae
96
57
P.panguanensis
Nelumbo
Malania
100
Vitis
Eucalyptus
Camellia
84
Mitrastemon
Rhododendron
Actinidia
66
55
Monotropa
Mimulus
50
Hyoscyamus
89
Nicotiana
Boea
Rhazya
74
Asclepias
Carica
71
Batis
Gossypium
100
Malva sylvestris
Cucurbita
89
Citrullus
Prunus
97
Malus
Hevea
67
Ricinus
Haematoxylum
100
Libidibia
Betula
54
Juglans
68
Fagus
76
Castanopsis
100
Quercus

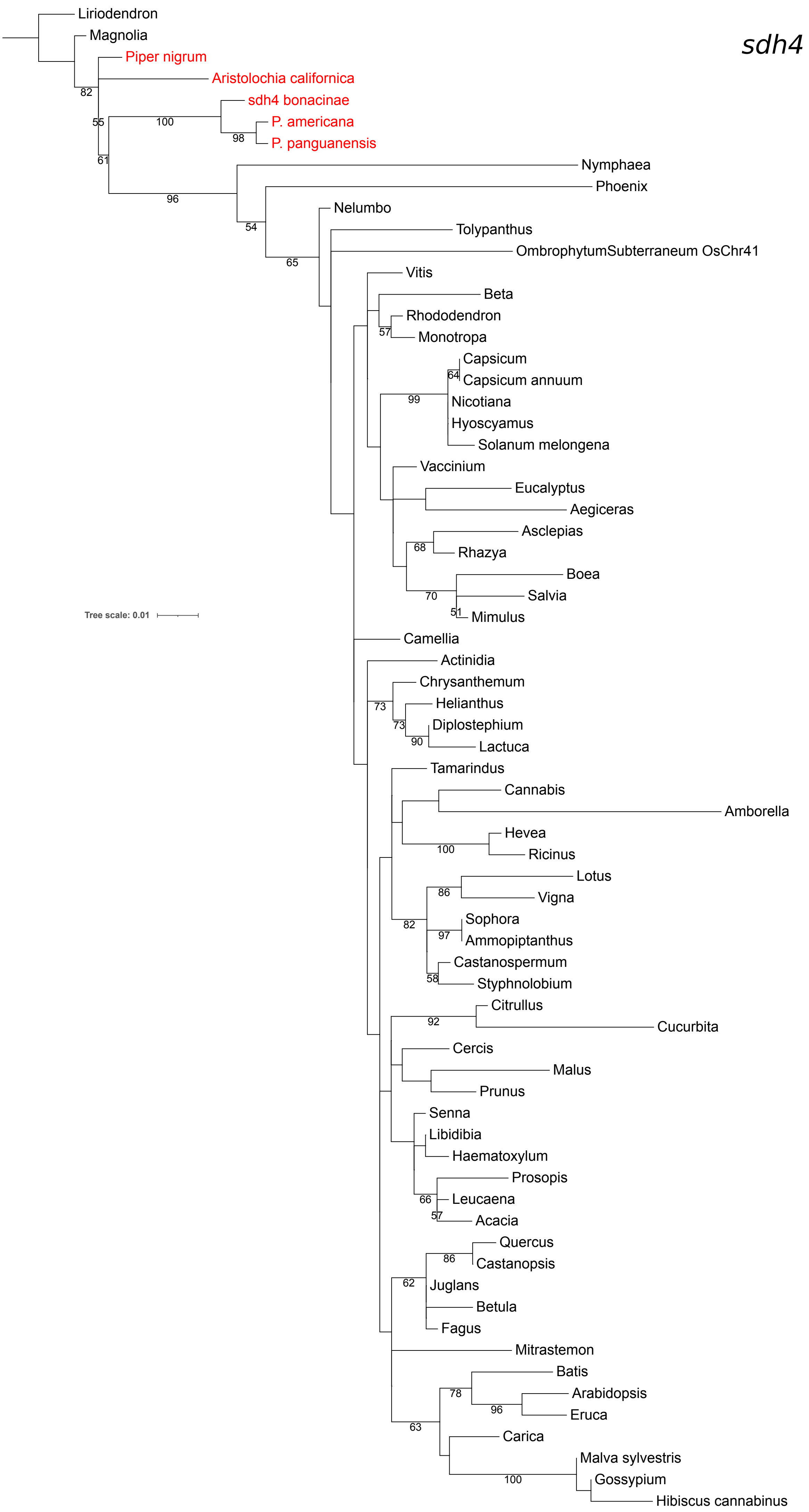

sdh4
Liriodendron
Magnolia
Piper nigrum
Aristolochia californica
sdh4 bonacinae
P. americana
P. panguanensis
Nymphaea
Phoenix
Nelumbo
Tolypanthus
OmbrophytumSubterraneum OsChr41
Vitis
Beta
Rhododendron
Monotropa
Capsicum
Capsicum annuum
Nicotiana
Hyoscyamus
Solanum melongena
Vaccinium
Eucalyptus
Aegiceras
Asclepias
Rhazya
Boea
Salvia
Mimulus
Camellia
Actinidia
Chrysanthemum
Helianthus
Diplostephium
Lactuca
Tamarindus
Cannabis
Amborella
Hevea
Ricinus
Lotus
Vigna
Sophora
Ammopiptanthus
Castanospermum
Styphnolobium
Citrullus
Cucurbita
Cercis
Malus
Prunus
Senna
Libidibia
Haematoxylum
Prosopis
Leucaena
Acacia
Quercus
Castanopsis
Juglans
Betula
Fagus
Mitrastemon
Batis
Arabidopsis
Eruca
Carica
Malva sylvestris
Gossypium
Hibiscus cannabinus
Tree scale: 0.01
