# Supplementary material for: Genomic Footprints of Multiple Host Lineages in the Mitochondrial and Nuclear Genomes of the Holoparasite Prosopanche americana
Source: Plants (Basel). 2026 Apr 7;15(7):1121. doi: 10.3390/plants15071121 (PMC13074556; doi:10.3390/plants15071121)

Figure S6. Functional landscape of horizontally transferred genes in the *Prosopanche americana* nuclear genome. HGT candidates were annotated using the Mercator pipeline and grouped by biological pathway (Y-axis) across the three main donor lineages (X-axis). The size of each circle is proportional to the number of transcripts assigned to each category. Note the specific enrichment of photosynthesis-related genes (e.g., Photosystem subunits, Light harvesting) derived exclusively from Solanaceae, in contrast to their absence in the current host lineage (Fabaceae).

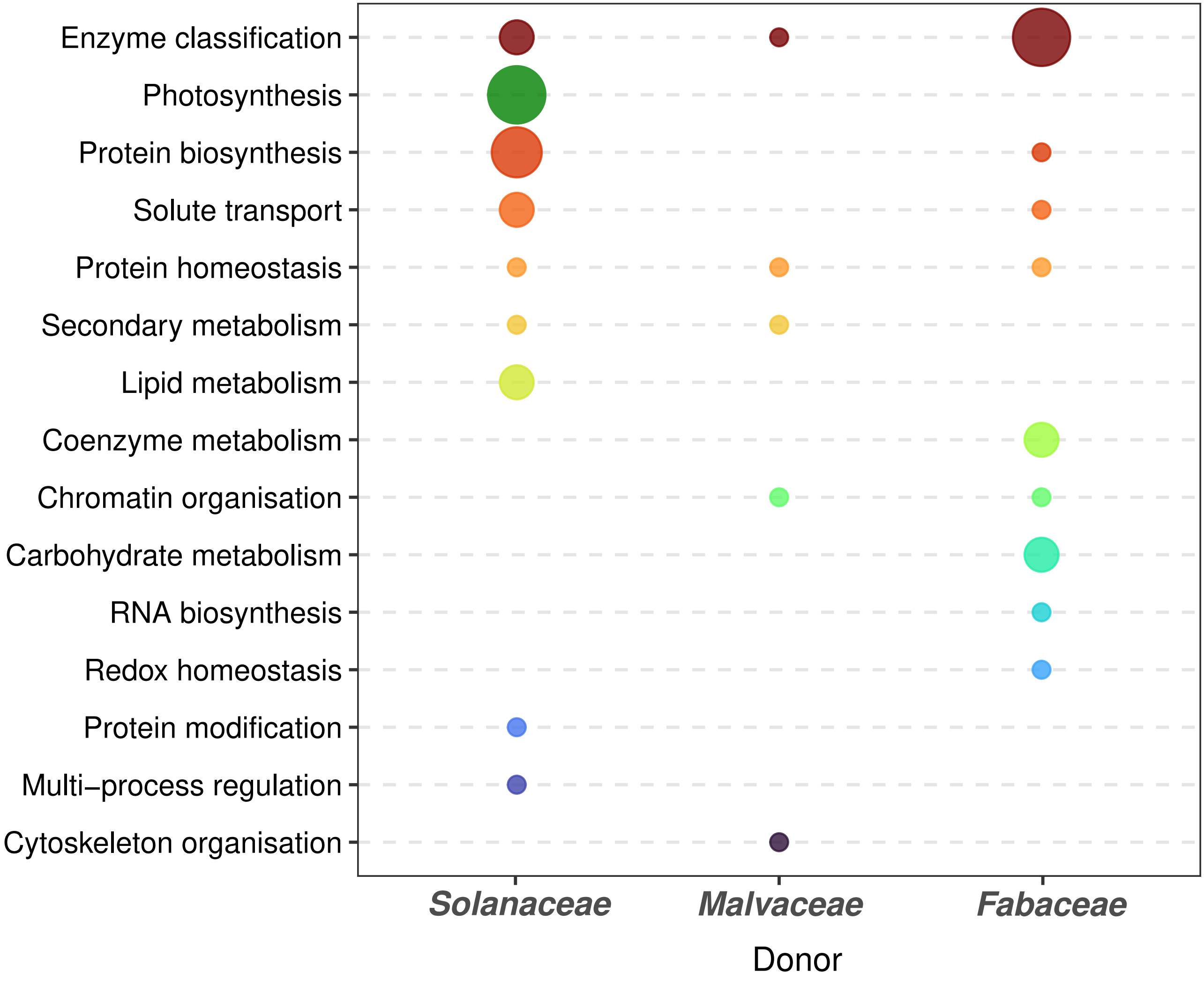

Supplement: Supplementary file 1 [file plants-15-01121-s001.zip › FigureS6.pdf]
